# Punctaporonins H–M: Caryophyllene-Type Sesquiterpenoids from the Sponge-Associated Fungus *Hansfordia sinuosae*

**DOI:** 10.3390/md12073904

**Published:** 2014-07-30

**Authors:** Zehong Wu, Dong Liu, Peter Proksch, Peng Guo, Wenhan Lin

**Affiliations:** 1 State Key Laboratory of Natural and Biomimetic Drugs, Peking University, Beijing 100191, China; E-Mails: wuzehong922@126.com (Z.W.); liudong_1982@126.com (D.L.); 2 Institute für Pharmazeutische Biologie und Biotechnologie, Heinrich-Heine-Universität Düsseldorf, Universitätsstr. 1, Geb.26.23, 40225 Düsseldorf, Germany; E-Mail: Proksch@uni-duesseldorf.de; 3 Pharmacology and Toxicology Research Center, Institute of Medicinal Plant Development, Chinese Academy of Medical Sciences, Peking Union Medical College, Beijing 100193, China

**Keywords:** sponge-associated fungus, *Hansfordia sinuosae*, punctaporonins H–M, structural elucidation, lowering lipid accumulation

## Abstract

Six new caryophyllene-based sesquiterpenoids named punctaporonins H–M (**1**–**6**), together with punctaporonin B (**7**) and humulane (**8**) were isolated from the fermentation broth of the sponge-derived fungus *Hansfordia sinuosae*. Their structures were determined by the extensive HRESIMS and NMR spectroscopic analysis, including the X-ray crystallographic data for the assignment of the absolute configurations of punctaporonins H–I (**1**–**2**). The isolated compounds were evaluated for antihyperlipidemic, cytotoxic and antimicrobial activities, and punctaporonin K (**4**) exhibited potent effects to reduce the triglycerides and total cholesterol in the intracellular levels.

## 1. Introduction

**Figure 1:**
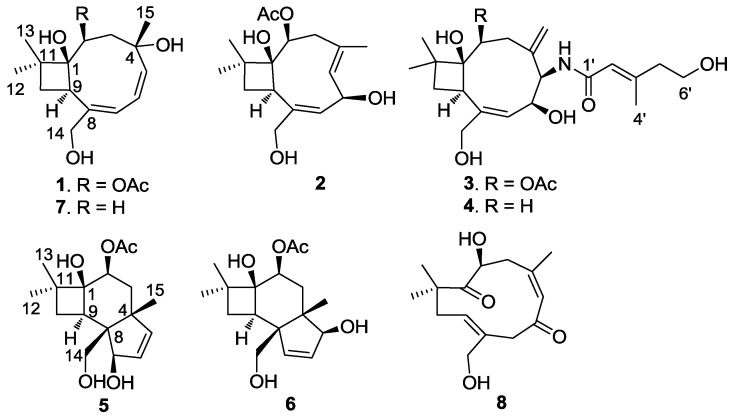
Structures of punctaporonins H–M (**1**–**6**), punctaporonin B (**7**) and humulane (**8**).

Caryophyllene-based sesquiterpenoids are a group of structurally unique natural products characterized by the presence of a bicyclo[2.7.0]undecane skeleton, in addition to the backbone-rearranged polycyclic derivatives [1]. The majority of caryophyllene-related analogues have been isolated from terrestrial plants [2,3,4] and the plant-associated fungal species [5,6,7,8,9,10,11,12]. Marine-derived caryophyllane-type compounds were found from the soft coral *Sinularia nanolobata* [13,14] and the gorgonian coral *Subergorgia suberosa* [15], while fuscoatrol A is the only caryophyllene sesquiterpene isolated from the marine fungus *Humicola fuscoatra* [16]. Some caryophyllene derivatives exhibited immunosuppressive [9], cytotoxic [15], and antibiotic activities [17,18]. As part of our ongoing search for the chemical diversity from marine-derived microorganisms, the sponge (*Niphates* sp.) associated fungus *Hansfordia* *sinuosae* (WGCA-25-3A) was isolated. Chemical examination of the ethyl acetate extract of the solid fermented *H.* *sinuosae* resulted in the isolation of six new sesquiterpenoids (**1**–**6**) together with two known analogues (Figure 1).

## 2. Results and Discussion

### 2.1. Structure Elucidation

**Figure 2:**
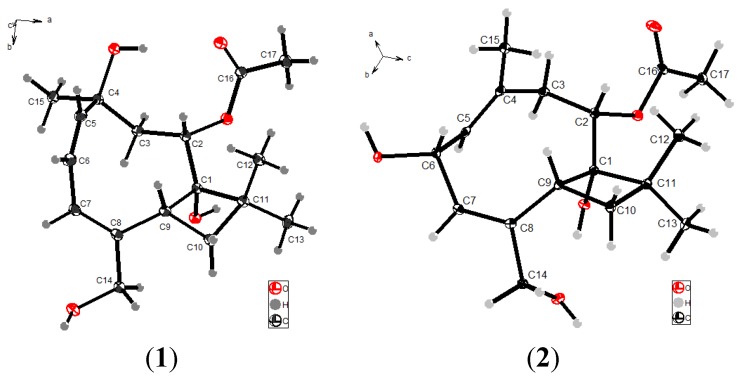
X-ray crystal structures of punctaporonins H (**1**) and I (**2**).

The molecular formula of punctaporonin H (**1**) was determined as C_17_H_26_O_5_ on the basis of its HRESIMS and NMR data, requiring five degrees of unsaturation. The IR absorptions at 3350 and 1708 cm^−1^ suggested the presence of hydroxy and carbonyl groups. Inspection of the ^13^C NMR and DEPT spectra revealed seventeen carbon resonances characterized by four olefinic carbons for two double bonds and a carbonyl carbon, while the ^1^H NMR spectrum displayed four methyl singlets, three olefinic protons, three methylene, and an oxymethine. Thus, the remaining degrees of the molecular unsaturation accounted for a bicyclic skeleton. A cyclobutane ring was evident from the COSY correlation between H-9 (δ_H_ 3.26, dd, *J* = 8.4, 11.0 Hz) and H_2_-10 (δ_H_ 1.42, 2.02) and their HMBC interactions with C-1 (δ_C_ 80.9) and C-11 (δ_C_ 40.6). Additional COSY relationships established the segments from CH-5 to CH-7 and CH-2 to CH_2_-3. The connection of the segments across a quaternary carbon C-4 (δ_C_ 72.1) was deduced by the HMBC correlations from H_3_-15 (δ_H_ 1.08 s) to C-3 (δ_C_ 44.9), C-4 (δ_C_ 72.1) and C-5 (δ_C_ 143.2), while C-4 was co-positioned by a methyl and a hydroxy group. The linkage of C-2 (δ_C_ 74.6) to C-1 and C-8 (δ_C_ 139.0) to C-9 (δ_C_ 39.0) to form a cyclononene ring was ascribed to the HMBC relationships from H-9 to C-7 (δ_C_ 123.6), C-8 and C-2, in addition to the correlations from H-7 (δ_H_ 5.81, brs) to C-9 and H-2 (δ_H_ 4.90, brd, *J* = 3.6 Hz) to C-1 and C-9. The location of a hydroxymethylene at C-8 was confirmed through the HMBC correlations from H_2_-14 (δ_H_ 3.87, 4.13, d, *J* = 13.2 Hz) to C-7, C-8 and C-9, whereas two methyl groups resonated at δ_H_ 1.03 (s) and 0.98 (s) were co-positioned at C-11 on the basis of their protons correlated to C-1, C-10 (δ_C_ 33.5) and C-11 in the HMBC spectrum. In addition, the HMBC correlation between H-2 and the acetyl carbonyl carbon (δ_C_ 170.8) clarified an acetoxy unit to be substituted at C-2. Thus, the gross structure was established as a caryophyllene-type sesquiterpene. The *J*_H-5/H-6_ (13.2 Hz) value and the NOE interaction between H-7 and H_2_-14 were in agreement with 5*Z* and 7*E* geometries. Additional NOE interactions from H_3_-12 to H-9 and H-2 indicated the same orientation of H-9 and H-2. The orientation of OH-1 was supposed to be opposite to H-9 on the basis of the NOE interaction between H-9 and H-2. The NOE correlations observed from H-15 to H-3b (δ_H_ 2.75) as well as from H-2 to H-3a (δ_H_ 1.36) indicated the opposite face of H_3_-15 toward H-2. Thus, the structure of **1** was determined as a C-2 acetoxylated punctaporonin B [6]. The absolute configuration of **1** was determined as 1*S*, 2*S*, 4*S*, and 9*R* by the results of the X-ray single-crystal diffraction using Flack parameters (Supplementary Information, Tables S1–S7 and Figure 2) [19].

**Figure 3:**
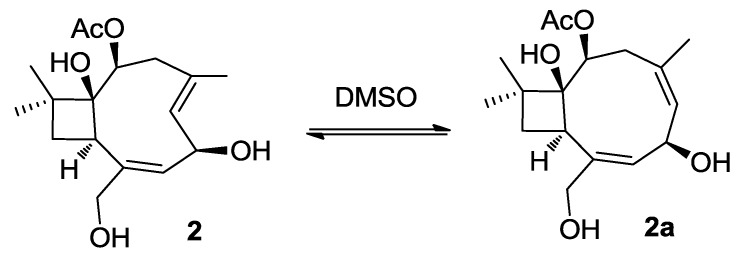
Auto-conversion of **2** to **2a** in DMSO.

The NMR spectroscopic data of punctaporonin I (**2**) were closely related to those of **1**, in fact both the compounds had the same molecular composition. However, C-6 (δ_C_ 66.1) was found to be a hydroxymethine group based on the HSQC correlation, and was located in the allylic position of the two double bonds according to the COSY and HMBC correlations observed. The latter displayed long range contacts from H_3_-15 (δ_H_ 1.83, s) to C-3 (δ_C_ 40.3), C-4 (δ_C_ 127.4) and C-5 (δ_C_ 133.4), revealing one of the double bonds to be between C-4/C-5. The comparable NOE interactions of **1** and **2** in addition to the NOE correlations from H-6 (δ_H_ 4.70) to H-9 (δ_H_ 3.04) and H_3_-15 and between H-5 (δ_H_ 5.17) and H-3b (δ_H_ 2.42, dd, *J* = 10.9, 10.8 Hz) confirmed a syn orientation for H-6 and H-9 and 4*E* geometry. Analysis of the X-ray single-crystal diffraction data (Supplementary Information, Tables S8–S14) revealed the absolute configurations of **2** to be 1*S*, 2*S*, 6*R*, and 9*R.* It is noteworthy that **2** is an unstable compound, which was partly converted to 4*Z* isomer **2a** with an ratio of 5:4 (**2**/**2a**) during the measurement of 2D NMR spectra in DMSO-*d*_6_ (Figure 3). This assignment was supported by the NOE interactions between H_3_-15/H-5 and H-7/H_2_-14 for **2a**.

**Figure 4:**
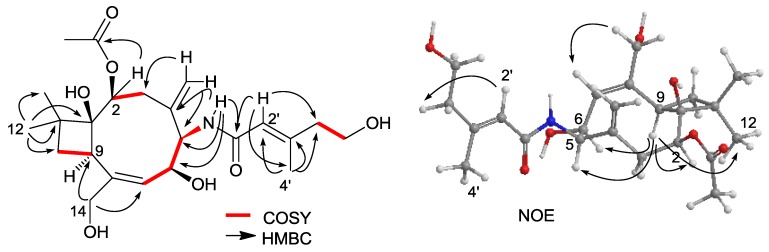
Key HMBC, COSY and NOE correlations of **3** (ChemBioDraw Ultra 12.0, Cambridgesoft, Cambridge, MA, USA).

The molecular formula of punctaporonin J (**3**) was established as C_23_H_35_NO_7_ by the HRESIMS data (*m*/*z* 438.2481 [M + H]^+^). The difference between **2** and **3** found in the NMR spectra was the presence of resonances of an exo-methylene at δ_H_ 4.79, 5.02/δ_C_ 119.6 and δ_C_ 143.5, an exchangeable proton at δ_H_ 7.79, and a side chain identified as (2*E*)-5-hydroxy-3-methylpent-2-enoyl group. The position of the exo-methylene group at C-4 was evident from its protons correlated to C-3 (δ_C_ 35.1), C-4 (δ_C_ 143.5) and C-5 (δ_C_ 62.5) in the HMBC spectrum. The side chain consisted of two olefinic carbons at δ_C_ 120.6 (C-2′) and 149.5 (C-3′), a carbonyl carbon at δ_C_ 166.4 (C-1′), two methylenes and a methyl carbon. The COSY relationship between H_2_-5′ (δ_H_ 2.21, t) and H_2_-6′ (δ_H_ 3.54, t), in association with the HMBC interactions from H_3_-4′ (δ_H_ 2.05, s ) to C-2′, C-3′ and C-5′ (δ_C_ 44.1) and from H-2′ (δ_H_ 5.76, s) to C-1′, C-4′ (δ_C_ 18.2) and C-5′ and the NOE interaction between H-2′ and H_2_-5′, established a (2*E*)-5-hydroxy-3-methylpent-2-enamide. The amide proton δ_H_ 7.79 (d) showing the COSY relationship with H-5 (δ_H_ 4.10, t) and the HMBC interactions with C-4, C-5 and C-6 confirmed the linkage of the amide moiety to C-5. The similar NOE interactions for the backbone of **3** and **2** in addition to the NOE correlations from H-9 (δ_H_ 3.26) to H-5 and H-6 (δ_H_ 4.36) assigned the same orientation of both H-5 and H-6 as H-9 (Figure 4).

The structure of punctaporonin K (**4**) was determined as 2-deacetoxy analogue of **3** on the basis of the similar NMR data of both compounds with the exception of the absence of acetoxy group and the presence of a methylene at C-2 (δ_C_ 32.9, δ_H_ 1.57, 1.94, m).

The molecular formula of punctaporonin L (**5**) was established as C_17_H_26_O_5_ by its HRESIMS (*m*/*z* 333.1677 [M + Na]^+^) and NMR data. Comparison of the NMR data revealed the structure of **5** closely related to 6-hydroxypunctaporonin E [6]. The distinction was attributed to C-2 (δ_C_ 71.4) of **5** being substituted by an acetoxy group (δ_C_ 170.2, 21.4; δ_H_ 1.94, s) instead of a hydroxy group, as evident from the HMBC interaction between H-2 (δ_H_ 4.53, dd, *J* = 4.8, 11.2 Hz) and the acetyl carbonyl carbon (δ_C_ 170.2). The relative configurations of **5** were in accordance with those of 6-hydroxypunctaporonin E on the basis of the similar NOE interactions of both compounds. Since the absolute configurations of 6-hydroxypunctaporonin E were determined through X-ray diffraction, the same sign and similar magnitude of the specific optical rotations for both compounds led us to assign **5** as possessing the same absolute configurations as the known analogue.

**Figure 5:**
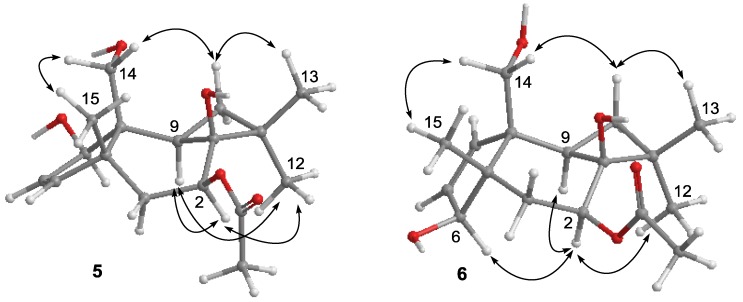
Key NOE interactions of **5** and **6** (ChemBioDraw Ultra 12.0).

The NMR data for punctaporonin M (**6**) were closely similar to those of **5**, except that H_3_-15 (δ_H_ 0.85, s) of **6** showed an HMBC correlation with an oxygenated methine carbon (δ_C_ 80.0, C-5) instead of an olefinic carbon, and H_2_-14 (δ_H_ 3.46, 3.53) exhibited the correlation with an olefinic carbon C-7 (δ_C_ 132.2), indicating that C-5 of **6** was substituted by a hydroxy group and an olefinic bond resided at C-6/C-7. The similar ROE correlations of **5** and **6** (Figure 5), in association with the NOE interaction of H-2 (δ_H_ 4.94, dd, *J* = 5.0, 10.8 Hz) showing with H-5(δ_H_ 4.77, brs), H-9 (δ_H_ 1.98, m), and H_3_-12 (δ_H_ 1.08, s), confirmed the same face of these protons. Additional NOE interaction between H_2_-14 and H_3_-15 was in agreement with the *cis*-fusion of the bicyclic ring (Figure 5).

Two known analogues were identical to punctaporonin B (**7**) [11] and humulane (**8**) [10] on the basis of the comparison of their NMR and specific optical rotations with those reported in literature.

### 2.2. Bioassay Results

All compounds showed weak cytotoxic activity against a panel of tumor cell lines including human colon carcinoma HCT-8, human hepatoma Bel7402, human gastric carcinoma BGC823, human lung adenocarcinoma A549, and human ovarian carcinoma A2780 with IC_50_ values >10 μM. These compounds also showed weak inhibitory effects against the bacterial strains of *Escherichia* *coli*, *Staphylococcus* *aureus*, *Bacillus* *thuringensis*, and *Bacillus* *subtilis* with the MIC values more than 125 μM.

**Figure 6:**
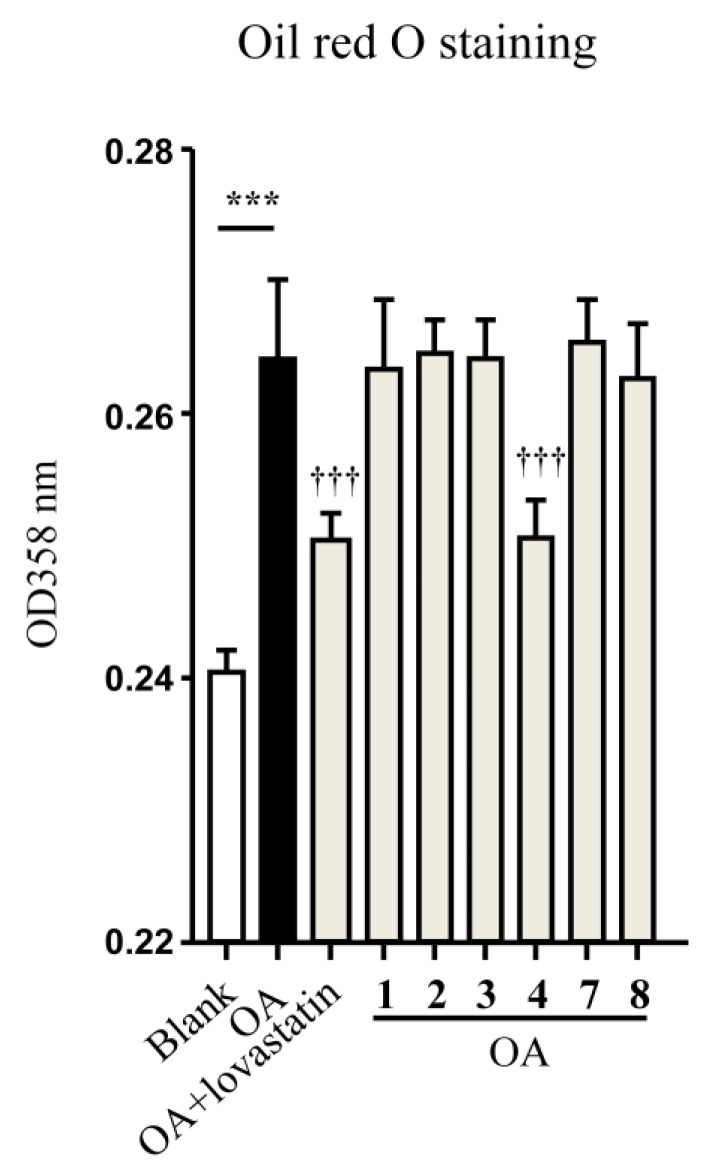
Effects of compounds on oleic acid-elicited intracellular lipid accumulation. Cells were incubated with DMEM (Dulbecco’s Modified Eagle Medium) + oleic acid (OA, 100 μM) for 12 h, and then treated with 10 μM of each compound with lovastatin as a positive control. The blank group was tested in DMEM alone, while DMEM + 100 μM OA was used as a negative control. Neutral lipids were determined by spectrophotometry at 358 nm after oil-red O staining. Bars depict the means ± SEM (standard error of mean) of at least three experiments. *** *p* < 0.001, OA *vs.* Blank; **^†††^** *p* < 0.001, test group *vs.* OA group.

**Figure 7:**
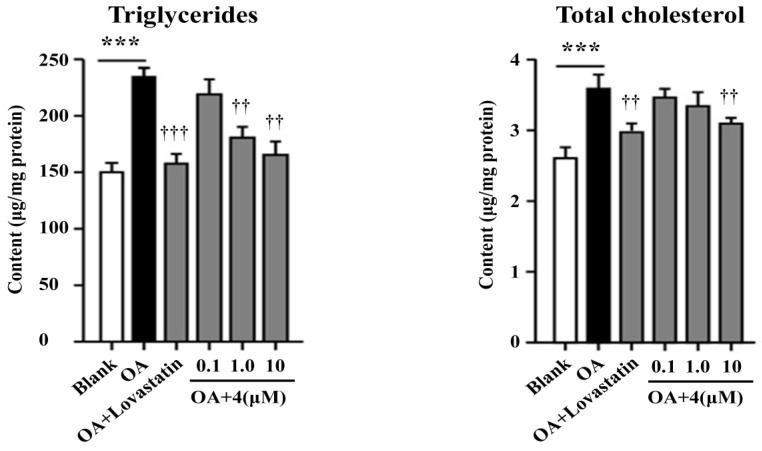
Inhibitory effects of **4** toward triglycerides and total cholesterol. Intracellular levels of triglycerides and total cholesterol were measured by kits according to the manufacturer’s instructions. Bars depict the means ± SEM of at least three experiments. *** *p* < 0.001, OA *vs.* Blank; **^††^** *p* < 0.01, **^†††^** *p* < 0.001, test group *vs.* OA group. OA: oleic acid.

Compounds **1**–**4** and **7**–**8** were tested for the lowering effects against oleic acid (OA)-elicited lipid accumulation in HepG2 liver cells. Compound **4** significantly reduced the OA-elicited lipid accumulation as measured by the oil-red O staining (Figure 6) [20], while the lowering effects of the intracellular total cholesterol (TC) and triglyceride (TG) quantification of **4** (Figure 7) were comparable to those induced by the positive control lovastatin and were in a dose dependent manner.

High cholesterol and/or triglyceride problems are known to increase risk for hypertension, diabetes mellitus, obesity and others that impact on coronary artery disease [21]. Lipid overaccumulation in liver is also a key cause for the development of insulin resistance [22,23]. High triglyceride levels are also a risk factor for acute pancreatitis. Decreasing lipid accumulation in liver is therefore beneficial for the prevention and treatment of diabetes. Modulating the dysregulation of lipid metabolism and decreasing the elevated levels of serum TC and TG are helpful for the treatment and prevention of cardiovascular disease [24]. Natural molecules such as resveratrol, cordycepin and chlorogenic acid have been investigated to be the capability of suppressing the lipogenesis and fat accumulation in liver tissues that induced obesity and diabetes [25,26]. However, regulation of lipid accumulation in cell level by caryophyllene-based derivatives has not been reported. In the present work, we reported caryophyllene-type analogue **4** as a new natural scaffold which could potently induce the reduction of TC and TG in liver cells for the first time.

## 3. Experimental Section

### 3.1. General Experimental Procedures

Optical rotations were measured using an Autopol III automatic polarimeter (Rudolph Research Co., Hackettstown, NJ, USA). Melting points were measured on JGW-360A melting point apparatus (Xmchongda, Tech. Co., Xiamen, China). IR spectra were recorded on a Thermo Nicolet Nexus 470 FT-IR spectrometer (Thermo Fisher Scientific Inc., New York, NY, USA). NMR spectra were measured on a Bruker Avance-500 FT NMR spectrometer (Bruker Co., Bremen, Germany) (500 MHz) using TMS as the internal standard. HRESIMS spectra were obtained on a FT-MS-Bruker APEX IV (7.0 T) (Bruker Co., Bremen, Germany). X-ray diffraction was obtained on Bruker D8 Advance single crystal X-ray Diffractometer (Bruker Co., Karlsruhe, Germany). TLC detection was carried out using precoated silica gel GF_254_ plates (Yantai Chem. Ind., Yantai, China). Column chromatography was performed on Silica gel (200–300 mesh, Qingdao Marine Chemical Plant, Qingdao, China). Sephadex LH-20 (18–110 μm) was obtained from Pharmacia Co. (Peapack, NJ, USA), and ODS (50 μm) was provided by YMC Co. (Kyoto, Japan) High-performance liquid chromatography (HPLC) was performed on a Waters e2695 Separation Module (Waters, Milford, USA) coupled with a Waters 2998 photodiode array detector (Waters, Milford, CT, USA). A Kromasil C_18_ semipreparative HPLC column (250 × 10 mm, 5 μm) (EKS Chemicals, Bohus, Sweden) was used for compound purification. All solvents were of analytical grade.

### 3.2. Fungal Material and Fermentation

The fungal strain *Hansfordia sinuosae* was isolated from the sponge of *Niphates* sp. collected from Southern China Sea. The fungus was identified by morphological observation and analysis of the ITS region of the rDNA, whose sequence data have been deposited at GenBank with the accession number KF877718. The strain (WGCA-23-3A) was deposited at the State Key Laboratory of Natural and Biomimetic Drugs, Peking University, China. The fungal strain was cultured on slants of potato dextrose agar (PDA) at 25 °C for 10 days. Spore inoculum was prepared by suspension in distilled H_2_O to give a final spore/cell suspension of 1 × 10^6^/mL. A large scale fermentation was carried out in fifty 500 mL Fernbach flasks each containing 80 g of rice. Distilled H_2_O with sterilization brine (100 mL) was added to each flask, and the contents were soaked overnight before autoclaving at 15 lb/in^2^ (pound per square inch) for 30 min. After cooling to room temperature, each flask was inoculated with 5.0 mL of the spore inoculum and incubated at 25 °C for 35 days.

### 3.3. Extraction and Isolation

The fermented material was extracted with EtOAc (3 × 10 L), and the organic layer was evaporated to dryness under vacuum to afford a crude extract (99.0 g). The extract was fractionated by a silica gel packed vacuum liquid chromatography (VLC) using petroleum ether–EtOAc gradient (5:1 to 1:1) as the eluent to afford six fractions (F1–F6). F4 (2.1 g) was subjected to an ODS column (10 µm) eluting with a MeOH–H_2_O gradient (from 30% to 100%) to yield five subfractions (F4A–F4E). F4A (150 mg) was subjected to a Sephadex LH-20 column eluting with MeOH to yield **8** (3.2 mg) and **7** (11.9 mg). F4E (300 mg) was separated on semipreparative HPLC (ODS) with 45% Acetonitrile-H_2_O as a mobile phase to afford **1** (3.0 mg, Rt 38.8 min) and **2** (32.3 mg, Rt 32.0 min). F5 (557 mg) was subjected to Sephadex LH-20 column eluting with MeOH to give **3** (13.0 mg) and **4** (5.8 mg), while **5** (1.8 mg, Rt 45.4 min) and **6** (3.0 mg, Rt 29.4 min) were separated by semipreparative HPLC with 36% Acetonitrile-H_2_O as a mobile phase.

**Punctaporonin H (1).** Colorless crystal. Mp. 152–154 °C, 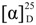 −83 (*c* 0.3, CH_3_OH). UV (CH_3_OH) λ_max_ 208, 198 nm; IR (KBr) ν_max_ 3350, 2961, 2934, 2870, 1708, 1530, 1383, 1263, 1122, 1024 cm^−1^; ^1^H and ^13^C NMR data, see Table 1; HRESIMS *m*/*z* 333.1675 [M + Na]^+^ (calcd for C_17_H_26_NaO_5_, 333.1672).

**Punctaporonin I (2).** Colorless crystal. Mp. 198–199 °C, 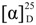 −139 (*c* 0.22, CH_3_OH). UV (CH_3_OH) λ_max_ 216, 198 nm; IR (KBr) ν_max_ 3343, 2932, 2870, 1714, 1648, 1620, 1463, 1373, 1256, 1122 cm^−1^; ^1^H and ^13^C NMR data, see Table 1; HRESIMS *m*/*z* 333.1669 [M + Na]^+^ (calcd for C_17_H_26_NaO_5_, 333.1672).

**Punctaporonin J (3).** Colorless oil, 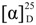 −37 (*c* 0.65, CH_3_OH). UV (CH_3_OH) λ_max_ 221.1 196.5 nm; IR (KBr) ν_max_ 3326, 2939, 2873, 1715, 1664, 1633, 1537, 1438, 1373, 1260, 1047, 1026 cm^−1^; ^1^H and ^13^C NMR data, see Table 1; HRESIMS *m*/*z* 438.2481 [M + H]^+^ (calcd for C_23_H_36_NO_7_, 438.2486).

**Punctaporonin K (4).** Colorless oil. 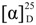 −64 (*c* 0.58, CH_3_OH). UV (CH_3_OH) λ_max_ 222, 195 nm; IR (KBr) ν_max_ 3295 (br), 2936, 2870, 1723, 1663, 1629, 1533, 1439, 1365, 1202, 1060 cm^−1^; ^1^H and ^13^C NMR data, see Table 1; HRESIMS *m*/*z* 380.2429 [M + H]^+^ (calcd for C_21_H_34_NO_5_, 380.2431).

**Table 1:** ^13^C and ^1^H NMR data for **1**–**6** in DMSO-*d*_6_.

No	1		2		3		4		5		6	
	δ_C_	δ_H_ (*J* in Hz)		δ_H_ (*J* in Hz)	δ_C_	δ_H_ (*J* in Hz)	δ_C_	δ_H_ (*J* in Hz)	δ_C_	δ_H_ (*J* in Hz)	δ_C_	δ_H_ (*J* in Hz)
1	80.9		81.2		83.4		82.7		77.8		77.5	
2	74.6	4.90, brd (3.6)	76.1	5.11, dd (10.9, 4.0)	77.4	5.08, dd (9.6, 5.2)	32.9	1.57, m; 1.94, m	71.4	4.53, dd (11.2, 4.8)	70.8	4.94, dd (10.8, 5.0)
3	44.9	1.36, d (15.8) 2.75, dd (15.8, 3.6)	40.3	2.13, dd (10.8, 4.0) 2.42, dd (10.9, 10.8)	35.1	2.19, dd (11.0, 9.6) 2.22, dd (11.0, 5.2)	28.7	1.95, brd (12.2) 2.05, m	39.0	1.66, dd (12.3, 4.8) 1.80, dd (12.3, 11.2)	36.5	1.62, dd (13.0, 10.8) 1.81, dd (13.0, 5.0)
4	72.1		127.4		143.5		148.3		52.6		54.4	
5	143.2	5.75, d (13.2)	133.4	5.17, d (9.8)	62.5	4.10, t (8.0)	62.3	4.24, t (8.5)	145.6	5.71, d (5.6)	80.0	4.77, brs
6	124.3	5.63, brd (13.2)	66.1	4.70, dd, (9.8, 4.2)	69.5	4.36, dd (9.0, 8.0)	69.6	4.43, dd (8.5,9.2)	130.1	5.61, dd (5.6, 2.6)	136.1	5.52, brd (6.0)
7	123.6	5.81, brs	141.8	5.83, s	133.3	5.38, d (9.0)	131.5	5.36, d (9.2)	80.3	3.94, d (2.6)	132.2	5.48, d (6.0)
8	139.0		136.1		136.1		137.2		49.4		53.5	
9	39.0	3.26, dd (11.0, 8.4)	40.6	3.04, dd (10.2, 7.9)	40.8	3.26, t (10.0)	43.9	3.16, dd (9.0, 8.7)	42.7	2.00, m	45.3	1.98, m
10	33.5	1.42, dd (9.1, 8.4) 2.02, dd (11.0, 9.1)	33.3	1.57, dd (12.0, 10.2) 1.92, dd (12.0, 7.9)	33.8	1.43, t (10.0) 2.04, t (10.0)	33.7	1.46, dd (9.0, 8.7) 2.02, t (9.0)	35.4	1.50, m 2.00, m	35.4	1.39, dd (5.0, 4.5) 2.00, m
11	40.6		41.6		41.0		40.9		41.6		42.5	
12	24.5	1.03, s	24.5	0.94, s	24.7	1.08, s	24.3	1.07, s	23.6	1.02, s	23.7	1.08, s
13	24.1	0.98, s	25.5	1.02, s	24.0	0.98, s	22.5	0.86, s	24.3	0.98, s	24.3	0.97, s
14	63.4	3.87, d (13.2) 4.13, d (13.2)	65.4	3.78, d (11.2) 4.11, d (11.2)	64.3	3.76, d (11.0) 3.94, d (11.0)	64.3	3.74, d (12.4) 3.90, d (12.4)	60.3	3.50, d (11.0) 3.93, d (11.0)	61.9	3.46, d (11.0) 3.53, d (11.0)
15	31.9	1.08, s	17.5	1.83, s	119.6	4.79, s; 5.02, s	117.9	4.66, s; 4.86, s	27.3	1.15, s	17.8	0.85, s
1′					166.4		166.4					
2′					120.6	5.76, s	120.6	5.76, s				
3′					149.5		149.4					
4′					18.2	2.05, s	18.2	2.07, s				
5′					44.1	2.21, t (6.2)	44.1	2.20, t (6.2)				
6′					59.6	3.54, t (6.5)	59.5	3.54, t (6.5)				
Ac	170.8		170.1		169.8				170.2		170.1	
	21.6	1.99, s	21.7	1.96, s	21.5	1.97, s			21.4	1.94, s	21.4	1.97, s
NH						7.79, d (8.0)		7.92, d (8.5)				

**Punctaporonin L (5).** White powder. 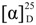 −48 (*c* 0.10, CH_3_OH); UV (CH_3_OH) λ_max_ 199 nm; IR (KBr) ν_max_ 3336, 2952, 2933, 2870, 1729, 1682, 1529, 1454, 1350, 1251, 1028 cm^−1^; ^1^H and ^13^C NMR data, see Table 1; HRESIMS *m*/*z* 333.1677 [M + Na]^+^ (calcd for C_17_H_26_O_5_Na, 333.1672).

**Punctaporonin M (6).** White powder. 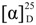 +49 (*c* 0.12, CH_3_OH). UV (CH_3_OH) λ_max_ 199 nm; IR (KBr) ν_max_ 3265, 2957, 2933, 2865, 1718 (br), 1463, 1377, 1249, 1017 cm^−1^; ^1^H and ^13^C NMR data, see Table 1; HRESIMS *m*/*z* 333.1677 [M + Na]^+^ (calcd for C_17_H_26_O_5_Na, 333.1672).

### 3.4. X-Ray Single Crystallographic Analyses

Punctaporonin H (**1**) and punctaporonin I (**2**) were crystallized from MeOH-CH_2_Cl_2_ (1:1) at room temperature. The X-ray crystallographic data of both compounds (see Supplementary information) were obtained on a Bruker SMART CCD detector employing graphite monochromated Cu-Kα radiation (operated in the φ-ω scan mode). The structures were solved by direct method using SHELXS-97 [27] and refined with full-matrix least-squares calculations on F2 using SHELXL-97 [27].

Crystal data of punctaporonin H: C_17_H_26_O_5_, *M* = 310.38, monoclinic, *a* = 9.3925(11) Å, *b* = 9.3490(9) Å, *c* = 9.8735(9) Å, *β* = 107.503(12)°, *U* = 826.86(15) Å^3^, *T* = 98.5, space group P2_1_ (No. 4), Z = 2, μ(Cu Kα) = 0.741, 5653 reflections measured, 2970 unique (*R*_int_ = 0.0315) which were used in all calculations. The final *wR*(*F*_2_) was 0.1288 (all data).

Crystal data of punctaporonin I: C_17_H_26_O_5_, *M* = 310.38, orthorhombic, *a* = 11.2742(10) Å, *b* = 11.7414(11) Å, *c* = 12.020(2) Å, *U* = 1591.2(4) Å^3^, *T* = 99.7, space group P2_1_2_1_2_1_ (No. 19), Z = 4, μ(Cu Kα) = 0.771, 5650 reflections measured, 2964 unique (*R*_int_ = 0.0301) which were used in all calculations. The final *wR*(*F*_2)_ was 0.0970 (all data).

The crystallographic data for the structures of punctaporonins H (**1**) and I (**2**) have been deposited in the Cambridge Crystallographic Data Centre (deposition numbers: CCDC 974950 for **1** and CCDC 974951 for **2**). Copies of the data can be obtained free of charge, on application to the director, CCDC [28], 12 Union Road, Cambridge CB21EZ, UK (Fax: +44-(0)1223-336033, or E-Mail: deposit@ccdc.cam.ac.uk). 

### 3.5. Cell-Based Lipid Accumulation Assay

HepG2 cells were maintained in DMEM medium supplemented with 10% fetal bovine serum and penicillin/streptomycin (100 μg/mL). The cells with 70%–80% confluence were incubated in DMEM + oleic acid (100 μM) for 12 h, then were treated with the compounds (each, 10 μM) and a positive control lovastatin in DMEM/100 μM oleic acid with DMEM/100 μM oleic acid as a blank for additional 6 h. Subsequently, the cells were subjected to oil-red O staining or TC and TG determination as described previously [20]. Each experiment (*n* = 8 for oil-red O staining or *n* = 3 for TC and TG determination) was repeated in triplication.

### 3.6. Cytotoxic and Antibacterial Assays

The cytotoxic assay was performed by a standard MTT method. Antimicrobial activities were measured against the bacterial strains of *Escherichia* *coli*, *Staphylococcus* *aureus*, *Bacillus* *thuringensis*, and *Bacillus* *subtilis*, by the broth microdilution method. The bacteria were grown for 16 h on a rotary shaker at 37 °C. Cultures were diluted with sterile medium to achieve an optical absorbance of 0.4–0.06 at 600 nm, then further diluted 10-fold before transferring into 96-well microtiter plates. Three replicates of each compound were tested in 125 μg/mL. The optical absorbance at 600 nm was measured after cultivation for 18 h. The lowest concentrations that completely inhibited visible growth of the tested strains were recorded from three independent experiments.

## 4. Conclusions

In biogenetic consideration, the tricyclic **5** was depicted to be derived from **1** through a transannular cyclization [29] by C-4/C-8 cycloaddition, whereas **6** was suggested to be derived from **5** via olefinic rearrangement. The 5-hydroxy-3-methyl-2-pentenamide unit in **3** and **4** was found from nature for the first time, while this unit was biogenetically considered to be generated through the mevalonate pathway. However, the mechanism for introducing nitrogen to form an amide instead of an ester is unknown. The significant effects of compound **4** to reduce the triglycerides and total cholesterol in the intracellular levels suggested it could be a potent lead compound for the development of a drug candidate for lowering lipids.

## Supplementary Files

Supplementary File 1 Supplementary Information (PDF, 2564 KB)
