# Supplementary material for: Punctaporonins H–M: Caryophyllene-Type Sesquiterpenoids from the Sponge-Associated Fungus Hansfordia sinuosae
Source: Mar Drugs. 2014 Jul 30;12(7):3904–16. doi: 10.3390/md12073904 (PMC4113805; doi:10.3390/md12073904)
Supplement: Supplementary File 1 — Supplementary Information (PDF, 2564 KB) [file marinedrugs-12-03904-s001.pdf]

Supplementary Information

1. X-ray crystallographic Data Punctaporonin H (1)

Figure S1. ORTEP view of punctaporonin H.

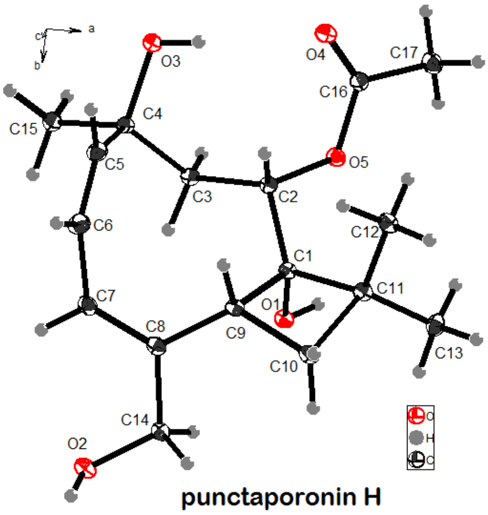

Table S1. Crystal data and structure refinement for punctaporonin H.

**Identification Code**	**Punctaporonin H**
Empirical formula	$C_{17}H_{26}O_5$
Formula weight	310.38
Temperature/K	98.5
Crystal system	monoclinic
Space group	$P2_1$
a/Å, b/Å, c/Å	9.3925(11), 9.3490(9), 9.8735(9)
α/°, β/°, γ/ °	90.00, 107.503(12), 90.00
Volume/$Å^3$	826.86(15)
Z	2
ρ_{calc}/mg mm^{-3}	1.247
μ/mm^{-1}	0.741
F(000)	336
Crystal size/mm^3	0.40 × 0.16 × 0.04
2θ range for data collection	9.88 to 139.68 °
Index ranges	$-11 \le h \le 11$, $-10 \le k \le 11$, $-12 \le l \le 11$
Reflections collected	5653
Independent reflections	2970[R(int) = 0.0315 (inf-0.9 Å)]
Data/restraints/parameters	2970/1/206
Goodness-of-fit on F^2	1.060
Final R indexes [I > 2σ (I) *i.e.*, $F_o > 4\sigma(F_o)$]	R_1 = 0.0480, wR_2 = 0.1259
Final R indexes [all data]	R_1 = 0.0495, wR_2 = 0.1288
Largest diff. peak/hole/e $Å^{-3}$	0.307/−0.192
Flack Parameters	−0.1(2)
Completeness	0.989

Table S2. Fractional Atomic Coordinates (×10^4) and Equivalent Isotropic Displacement Parameters ($Å^2$ × 10^3) for punctaporonin H. U_{eq} is defined as 1/3 of the trace of the orthogonalised U_{TT} tensor.

Atom	*x*	*y*	*z*	U(eq)
O3	−7597.3(16)	−3851.2(17)	−3404.7(16)	24.0(3)
O1	−4254.8(16)	306.5(16)	−743.5(15)	21.0(3)
O4	−4443.2(18)	−4398.2(18)	−3003.3(18)	29.1(4)
O5	−3803.9(16)	−2572.7(16)	−1463.3(15)	22.4(3)
O2	−7567.3(18)	3642.4(18)	−1712.0(17)	27.3(4)
C2	−5035(2)	−1708(2)	−2335(2)	18.9(4)
C4	−7839(2)	−2415(2)	−2964(2)	21.1(4)
C3	−6429(2)	−1885(2)	−1840(2)	20.3(4)
C10	−3969(2)	1653(2)	−3269(2)	23.8(4)
C13	−1599(2)	430(3)	−1561(2)	27.9(5)
C9	−5430(2)	892(2)	−3239(2)	20.1(4)
C11	−3098(2)	251(2)	−2703(2)	22.3(4)
C12	−2932(3)	−718(3)	−3899(2)	25.3(5)
C5	−8406(3)	−1502(3)	−4288(2)	27.0(5)
C16	−3622(2)	−3876(2)	−1933(2)	22.8(4)
C8	−6718(2)	1622(2)	−2909(2)	21.9(4)
C15	−9095(2)	−2572(3)	−2290(2)	28.2(5)
C7	−8103(3)	1119(3)	−3418(2)	25.2(4)
C17	−2263(3)	−4597(3)	−974(3)	32.8(5)
C6	−8566(3)	−85(3)	−4449(3)	31.0(5)
C14	−6333(2)	2922(2)	−1966(2)	23.6(5)
C1	−4450(2)	−186(2)	−2150(2)	19.2(4)

Table S3. Anisotropic Displacement Parameters ($Å^2 \times 10^3$) for punctaporonin H. The Anisotropic displacement factor exponent takes the form: $-2\pi^2[h^2a^{*2}U_{11}+k^2b^{*2}U_{22}+1^2c^{*2}U_{33}+2hka^*b^*U_{12}]$.

Atom	U11	U22	U33	U23	U13	U12
O3	22.5(7)	21.5(8)	28.1(8)	−3.7(6)	7.9(6)	0.4(6)
O1	23.5(7)	21.0(8)	17.8(7)	−0.1(6)	5.2(5)	2.1(6)
O4	29.0(8)	23.6(9)	33.7(8)	−4.4(7)	8.1(7)	1.3(6)
O5	23.3(7)	20.8(8)	21.6(7)	1.5(6)	4.6(6)	2.7(6)
O2	31.9(8)	24.3(8)	27.1(8)	0.4(6)	11.1(6)	6.4(7)
C2	20.5(9)	19.4(11)	15.4(9)	0.9(7)	3.3(7)	1.7(8)
C4	21.9(10)	18(1)	22.5(10)	−2.3(8)	5.5(8)	0.3(8)
C3	24.5(10)	18.2(10)	18.1(9)	1.6(8)	6.3(8)	0.5(8)
C10	29.2(11)	20.6(11)	24.6(10)	4.1(8)	12.7(8)	2.9(9)
C13	23.3(10)	34.0(12)	28.3(11)	−0.7(9)	10.7(9)	−2.4(9)
C9	24.7(10)	18.7(11)	17.3(9)	0.2(8)	7.1(7)	1.3(8)
C11	25.3(10)	21.0(11)	22.4(10)	1.7(8)	9.9(8)	1.1(8)
C12	28.2(10)	26.8(12)	23.9(10)	3.6(9)	12.3(8)	4.2(9)
C5	26.7(11)	27.1(12)	21.8(10)	−3.6(8)	−0.9(8)	−1.4(9)
C16	22.8(10)	19.5(10)	28.6(10)	6.1(8)	11.6(8)	1.2(8)
C8	26.2(10)	19.3(10)	18.7(10)	5.8(8)	4.2(8)	5.2(8)
C15	24.3(11)	29.2(12)	31.2(11)	−6.7(10)	8.7(9)	−0.7(9)
C7	25.8(10)	20.8(11)	27.2(10)	6.1(9)	5.3(8)	6.8(8)
C17	28.8(11)	26.2(12)	41.6(13)	7.6(10)	7.9(10)	6.6(10)
C6	29.8(12)	29.0(12)	26.6(11)	4.1(9)	−2.7(9)	0.2(9)
C14	24.3(11)	22.4(11)	24(1)	0.1(8)	7.0(8)	3.6(8)
C1	20.1(10)	18.6(10)	19.2(9)	0.4(8)	6.4(8)	0.9(8)

Table S4. Bond Lengths for punctaporonin H.

Atom	Atom	Length/Å	Atom	Atom	Length/Å
O3	C4	1.450(3)	C10	C11	1.557(3)
O1	C1	1.422(3)	C13	C11	1.525(3)
O4	C16	1.208(3)	C9	C8	1.508(3)
O5	C2	1.460(2)	C9	C1	1.557(3)
O5	C16	1.333(3)	C11	C12	1.533(3)
O2	C14	1.427(3)	C11	C1	1.579(3)
C2	C3	1.537(3)	C5	C6	1.337(4)
C2	C1	1.516(3)	C16	C17	1.502(3)
C4	C3	1.532(3)	C8	C7	1.331(3)
C4	C5	1.517(3)	C8	C14	1.507(3)
C4	C15	1.526(3)	C7	C6	1.492(4)
C10	C9	1.554(3)			

Table S5. Bond Angles for punctaporonin H.

Atom	Atom	Atom	Angle/°	Atom	Atom	Atom	Angle/°
C16	O5	C2	117.54(16)	C12	C11	C10	112.65(18)
O5	C2	C3	110.61(15)	C12	C11	C1	113.06(18)
O5	C2	C1	104.74(15)	C6	C5	C4	131.1(2)
C1	C2	C3	112.06(17)	O4	C16	O5	124.0(2)
O3	C4	C3	109.71(17)	O4	C16	C17	124.6(2)
O3	C4	C5	107.98(17)	O5	C16	C17	111.39(19)
O3	C4	C15	104.66(17)	C7	C8	C9	121.0(2)
C5	C4	C3	116.44(18)	C7	C8	C14	122.9(2)
C5	C4	C15	108.22(18)	C14	C8	C9	116.08(18)
C15	C4	C3	109.18(17)	C8	C7	C6	125.1(2)
C4	C3	C2	115.99(17)	C5	C6	C7	131.8(2)
C9	C10	C11	88.84(16)	O2	C14	C8	115.62(18)
C10	C9	C1	88.12(15)	O1	C1	C2	110.92(17)
C8	C9	C10	124.55(19)	O1	C1	C9	109.90(17)
C8	C9	C1	119.37(17)	O1	C1	C11	111.89(17)
C10	C11	C1	87.21(15)	C2	C1	C9	113.98(16)
C13	C11	C10	116.21(19)	C2	C1	C11	120.11(18)
C13	C11	C12	110.46(19)	C9	C1	C11	87.93(15)
C13	C11	C1	115.60(17)				

Table S6. Torsion Angles for punctaporonin H.

A	B	C	D	Angle/°
O3	C4	C3	C2	64.2(2)
O3	C4	C5	C6	−167.9(3)
O5	C2	C3	C4	−122.53(18)
O5	C2	C1	O1	−68.34(19)
O5	C2	C1	C9	166.99(15)
O5	C2	C1	C11	64.7(2)
C2	O5	C16	O4	−2.8(3)
C2	O5	C16	C17	176.51(17)
C4	C5	C6	C7	7.5(5)
C3	C2	C1	O1	51.6(2)
C3	C2	C1	C9	−73.0(2)
C3	C2	C1	C11	−175.30(17)
C3	C4	C5	C6	−44.0(4)
C10	C9	C8	C7	153.3(2)
C10	C9	C8	C14	−27.5(3)
C10	C9	C1	O1	91.60(18)
C10	C9	C1	C2	−143.19(18)
C10	C9	C1	C11	−20.97(16)
C10	C11	C1	O1	−89.71(18)
C10	C11	C1	C2	137.62(19)
C10	C11	C1	C9	20.93(16)

Table S6. *Cont.*

A	B	C	D	Angle/°
C13	C11	C1	O1	28.1(3)
C13	C11	C1	C2	−104.5(2)
C13	C11	C1	C9	138.8(2)
C9	C10	C11	C13	−138.24(18)
C9	C10	C11	C12	92.90(19)
C9	C10	C110	C1	−20.97(15)
C9	C8	C7	C6	−6.3(3)
C9	C8	C14	O2	177.31(17)
C11	C10	C9	C8	146.24(19)
C11	C10	C9	C1	21.27(15)
C12	C11	C1	O1	156.82(17)
C12	C11	C1	C2	24.1(3)
C12	C11	C1	C9	−92.54(19)
C6	C7	C8	C2	−58.8(2)
C16	O5	C2	C3	94.1(2)
C16	O5	C2	C1	−144.96(17)
C8	C9	C1	O1	−37.6(3)
C8	C9	C1	C2	87.6(2)
C8	C9	C1	C11	−150.21(19)
C8	C7	C6	C5	76.7(4)
C15	C4	C3	C2	178.37(18)
C15	C4	C5	C6	79.3(3)
C7	C8	C14	O2	−3.6(3)
C14	C8	C7	C6	174.6(2)
C1	C2	C3	C4	121.0(2)
C1	C9	C8	C7	−96.7(2)
C1	C9	C8	C14	82.5(2)

Table S7. Hydrogen Atom Coordinates (Å × 10^4) and Isotropic Displacement Parameters (Å^2 × 10^3) for punctaporonin H.

Atom	*x*	*y*	*z*	U(eq)
H3	−6764	−3904	−3507	36
H1	−3619	−181	−182	31
H2	−7803	4331	−2247	41
H9	−5265	−1993	−3333	23
H8A	−6192	−2551	−1051	24
H8B	−6650	−970	−1487	24
H11A	−3692	2454	−2618	29
H11B	−3955	1920	−4214	29
H17A	−1698	1117	−871	42
H17B	−867	759	−1989	42
H17C	−1289	−472	−1102	42
H2A	−5831	387	−4144	24
H16A	−2174	−338	−4269	38
H16B	−3864	−760	−4643	38
H16C	−2652	−1662	−3533	38
H6	−8692	−2016	−5133	32
H13A	−8816	−3268	−1542	42
H13B	−9989	−2878	−2997	42
H13C	−9269	−1668	−1907	42
H4	−8839	1552	−3106	30
H15A	−1468	−4560	−1399	49
H15B	−2492	−5577	−837	49
H15C	−1958	−4116	−74	49
H5	−9057	203	−5373	37
H12A	−5651	2633	−1059	28
H12B	−5808	3596	−2391	28

2. X-ray Crystallographic Data Punctaporonin I (2)

Figure S2. ORTEP view of punctaporonin I.

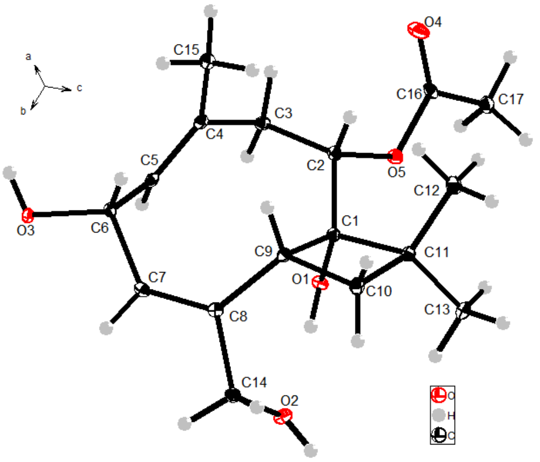

Table S8. Crystal data and structure refinement for punctaporonin I.

**Identification Code**	**Punctaporonin I**
Empirical formula	$C_{17}H_{26}O_5$
Formula weight	310.38
Temperature/K	99.7
Crystal system	orthorhombic
Space group	P212121
a/Å, b/Å, c/Å	11.2742(10), 11.7414(11), 12.020(2)
α/°, β/°, γ/°	90.00, 90.00, 90.00
Volume/$Å^3$	1591.2(4)
Z	4
ρ_{calc}/mg mm^{-3}	1.296
μ/mm^{-1}	0.771
F(000)	672
Crystal size/mm^3	0.20 × 0.10 × 0.08
2Θ range for data collection	10.54 to 139.92 °
Index ranges	$-13 \leq h \leq 13$, $-14 \leq k \leq 8$, $-14 \leq l \leq 14$
Reflections collected	5650
Independent reflections	2964[R(int) = 0.0301 (inf-0.9 Å)]
Data/restraints/parameters	2964/0/206
Goodness-of-fit on F^2	1.047
Final R indexes [I > 2σ (I) *i.e.*, $F_o > 4\sigma (F_o)$]	$R_1 = 0.0372$, $wR_2 = 0.0948$
Final R indexes [all data]	$R_1 = 0.0394$, $wR_2 = 0.0970$
Largest diff. peak/hole/e Å^{-3}	0.309/−0.222
Flack Parameters	0.11(18)
Completeness	0.986

Table S9. Fractional Atomic Coordinates (×10[4]) and Equivalent Isotropic Displacement Parameters (Å[2] × 10[3]) for punctaporonin I. U_{eq} is defined as 1/3 of the trace of the orthogonalised U_{TT} tensor.

Atom	*x*	*y*	*z*	U(eq)
O5	2308.2(11)	245.8(10)	2115.4(11)	15.9(3)
O1	1631.4(11)	2327.6(10)	1688.5(11)	15.2(3)
O2	1193.3(11)	4595.0(11)	2116.0(11)	19.2(3)
O4	3635.0(12)	−1178.8(11)	2239.9(14)	27.0(3)
O3	4972.0(11)	4468.7(11)	−1022.3(11)	17.9(3)
C13	1360.8(17)	2200.2(17)	4039.7(16)	21.9(4)
C2	3240.5(15)	1062.4(15)	1850.4(15)	14.2(4)
C5	4137.0(15)	2791.0(15)	−89.1(14)	14.0(3)
C7	3824.2(16)	4638.1(15)	646.1(15)	15.2(4)
C9	3510.9(15)	3323.1(15)	2302.0(14)	13.7(4)
C1	2716.4(15)	2197.3(15)	2265.5(15)	13.3(4)
C4	4483.7(17)	1820.2(16)	369.8(14)	14.7(4)
C10	3208.0(17)	3447.7(16)	3561.3(15)	17.2(4)
C6	4724.1(15)	3925.6(15)	27.3(16)	14.5(3)
C12	3408.5(19)	1411.2(17)	4213.1(16)	22.4(4)
C8	3245.9(16)	4359.2(15)	1569.9(15)	13.7(4)
C11	2624.2(16)	2253.4(16)	3584.9(14)	15.4(4)
C15	5702.3(17)	1585.5(16)	822.7(16)	18.9(4)
C14	2302.8(16)	5179.1(15)	1971.4(15)	16.7(4)
C3	3539.3(16)	950.9(15)	612.5(15)	16.2(4)
C16	2630.0(16)	−849.5(15)	2256.7(15)	16.3(4)
C17	1548.6(18)	−1569.1(16)	2433.6(16)	20.0(4)

Table S10. Anisotropic Displacement Parameters (Å^2 × 10^3) for punctaporonin I. The Anisotropic displacement factor exponent takes the form: $-2\pi^2[h^2a*^2U_{11} + ... + 2hka \times b \times U_{12}]$.

Atom	U11	U22	U33	U23	U13	U12
O5	14.0(6)	13.1(6)	20.6(6)	1.7(5)	−0.2(5)	−1.0(5)
O1	14.6(6)	12.5(6)	18.4(6)	0.1(5)	−2.6(5)	1.0(5)
O2	15.6(6)	19.6(6)	22.5(7)	−4.8(5)	2.9(5)	0.6(5)
O4	20.3(7)	19.8(7)	41.0(8)	5.8(6)	−3.6(6)	4.7(6)
O3	17.2(6)	21.3(7)	15.3(6)	6.9(5)	5.1(5)	3.0(5)
C13	22.6(10)	25.4(10)	17.8(9)	1.4(8)	5.9(8)	0.6(8)
C2	13.0(8)	13.3(8)	16.3(9)	1.1(7)	−0.8(7)	−2.5(7)
C5	12.8(8)	19.5(9)	9.6(8)	−2.7(7)	0.5(7)	0.7(7)
C7	15.2(9)	12.0(8)	18.5(8)	−0.4(7)	−3.3(7)	−1.7(7)
C9	14.4(9)	14.3(9)	12.4(8)	−1.9(7)	0.4(7)	−0.1(7)
C1	12.5(8)	14.0(8)	13.3(8)	1.6(7)	0.1(7)	−1.2(7)
C4	17.8(9)	15.8(8)	10.5(8)	−4.3(6)	1.6(7)	0.4(7)
C10	20.4(9)	18.1(9)	13.0(8)	−0.5(7)	−1.2(7)	−1.7(7)
C6	14.2(8)	14.9(8)	14.4(8)	4.1(7)	−1.1(7)	−1.7(7)
C12	28.2(11)	25.6(10)	13.3(9)	2.5(7)	−3.1(8)	4.1(8)
C8	12.4(8)	12.8(8)	15.9(8)	−3.8(7)	−2.8(7)	−1.9(7)
C11	16.2(9)	16.9(8)	13.1(8)	−0.1(7)	0.5(7)	−0.3(7)
C15	18.3(9)	18.4(9)	19.8(9)	1.5(7)	1.0(7)	1.5(7)
C14	18.2(9)	13.9(8)	18.0(9)	−1.1(7)	1.2(7)	0.0(7)
C3	18.3(9)	14.0(8)	16.2(8)	−3.9(7)	−0.5(7)	1.9(7)
C16	21.8(9)	14.0(8)	13.1(8)	1.4(7)	−3.8(7)	0.4(7)
C17	26.1(10)	16.3(9)	17.6(9)	1.4(7)	2.5(7)	−2.0(8)

Table S11. Bond Lengths for punctaporonin I.

Atom	Atom	Length/Å	Atom	Atom	Length/Å
O5	C2	1.458(2)	C7	C8	1.329(3)
O5	C16	1.347(2)	C9	C1	1.597(2)
O1	C1	1.414(2)	C9	C10	1.559(2)
O2	C14	1.437(2)	C9	C8	1.531(2)
O4	C16	1.197(2)	C1	C11	1.591(2)
O3	C6	1.441(2)	C4	C15	1.503(3)
C13	C11	1.527(3)	C4	C3	1.504(3)
C2	C1	1.541(2)	C10	C11	1.549(3)
C2	C3	1.531(2)	C12	C11	1.526(3)
C5	C4	1.325(3)	C8	C14	1.513(2)
C5	C6	1.494(2)	C16	C17	1.498(3)
C7	C6	1.511(3)			

Table S12. Bond Angles for punctaporonin I.

Atom	Atom	Atom	Angle/°	Atom	Atom	Atom	Angle/°
C16	O5	C2	117.47(14)	C11	C10	C9	91.48(14)
O5	C2	C1	102.80(13)	O3	C6	C5	113.48(15)
O5	C2	C3	108.35(14)	O3	C6	C7	108.44(14)
C3	C2	C1	118.23(15)	C5	C6	C7	104.02(14)
C4	C5	C6	126.67(17)	C7	C8	C9	125.48(16)
C8	C7	C6	127.19(16)	C7	C8	C14	117.03(16)
C10	C9	C1	88.94(13)	C14	C8	C9	117.35(15)
C8	C9	C1	122.16(14)	C13	C11	C1	114.59(15)
C8	C9	C10	116.16(14)	C13	C11	C10	116.09(16)
O1	C1	C2	105.45(14)	C10	C11	C1	89.51(14)
O1	C1	C9	114.14(14)	C12	C11	C13	109.68(16)
O1	C1	C11	115.33(14)	C12	C11	C1	115.38(15)
C2	C1	C9	120.63(14)	C12	C11	C10	110.44(16)
C2	C1	C11	112.57(15)	O2	C14	C8	110.28(14)
C11	C1	C9	88.57(13)	C4	C3	C2	106.64(14)
C5	C4	C15	125.31(17)	O5	C16	C17	109.70(15)
C5	C4	C3	117.11(17)	O4	C16	O5	124.13(18)
C15	C4	C3	116.91(16)	O4	C16	C17	126.17(17)

Table S13. Torsion Angles for punctaporonin I.

A	B	C	D	Angle/°
O5	C2	C1	O1	−57.55(16)
O5	C2	C1	C9	171.39(13)
O5	C2	C1	C11	69.00(17)
O5	C2	C3	C4	178.92(14)
O1	C1	C11	C13	11.7(2)
O1	C1	C11	C10	−106.99(16)
O1	C1	C11	C12	140.51(16)
C2	O5	C16	O4	4.4(3)
C2	O5	C16	C17	−175.61(13)
C2	C1	C11	C13	−109.38(18)
C2	C1	C11	C10	131.95(15)
C2	C1	C11	C12	19.5(2)
C5	C4	C3	C2	−99.14(18)
C7	C8	C14	O2	125.76(17)
C9	C1	C11	C13	127.84(16)
C9	C1	C11	C10	9.16(13)
C9	C1	C11	C12	−103.34(17)
C9	C10	C11	C13	−126.73(16)
C9	C10	C11	C1	−9.39(14)
C9	C10	C11	C12	107.63(15)
C9	C8	C14	O2	−58.3(2)
C1	C2	C3	C4	62.6(2)

Table S13. *Cont.*

A	B	C	D	Angle/°
C1	C9	C10	C11	9.35(13)
C1	C9	C8	C7	−102.3(2)
C1	C9	C8	C14	82.1(2)
C4	C5	C6	O3	127.69(19)
C4	C5	C6	C7	−114.67(19)
C10	C9	C1	O1	108.14(15)
C10	C9	C1	C2	−124.65(16)
C10	C9	C1	C11	−9.10(13)
C10	C9	C8	C7	151.14(17)
C10	C9	C8	C14	−24.4(2)
C6	C5	C4	C15	−18.5(3)
C6	C5	C4	C3	151.71(17)
C6	C7	C8	C9	9.9(3)
C6	C7	C8	C14	−174.53(16)
C8	C7	C6	O3	170.24(16)
C8	C7	C6	C5	49.2(2)
C8	C9	C1	O1	−12.5(2)
C8	C9	C1	C2	114.74(19)
C8	C9	C1	C11	−129.71(16)
C8	C9	C10	C11	135.09(16)
C15	C4	C3	C2	71.94(19)
C3	C2	C1	O1	61.68(19)
C3	C2	C1	C9	−69.4(2)
C3	C2	C1	C11	−171.77(15)
C16	O5	C2	C1	−155.30(15)
C16	O5	C2	C3	78.81(18)

Table S14. Hydrogen Atom Coordinates (Å × 10^4) and Isotropic Displacement Parameters (Å^2 × 10^3) for punctaporonin I.

Atom	*x*	*y*	*z*	U(eq)
H1	1377	2975	1784	23
H2	828	4882	2635	29
H3	5503	4122	−1343	27
H17A	888	2779	3692	33
H17B	1374	2323	4829	33
H17C	1026	1465	3884	33
H9	3948	881	2290	17
H6	3459	2763	−529	17
H4	3657	5351	347	18
H2A	4348	3114	2222	16
H11A	3904	3506	4033	21
H11B	2657	4062	3716	21
H5	5453	3854	466	17
H16A	3401	1593	4992	34
H16B	4206	1459	3937	34
H16C	3112	653	4106	34
H13A	6242	2160	564	28
H13B	5965	851	573	28
H13C	5678	1596	1621	28
H12A	2547	5514	2673	20
H12B	2207	5789	1434	20
H8A	3827	191	450	19
H8B	2841	1095	162	19
H15A	1154	−1333	3103	30
H15B	1778	−2354	2499	30
H15C	1021	−1482	1813	30

3. The HRESIMS, IR and NMR Data of Punctaporonin H (1)

Figure S3. HRESIMS spectrum of punctaporonin H (**1**).

Peking University Mass Spectrometry Sample Analysis Report

Analysis Info

Analysis Name	13060961_20130626_000008.d	Acquisition Date	6/26/2013 3:49:22 PM
Sample	8	Instrument	Bruker Apex IV FTMS
Comment	ESI Positive	Operator	Peking University

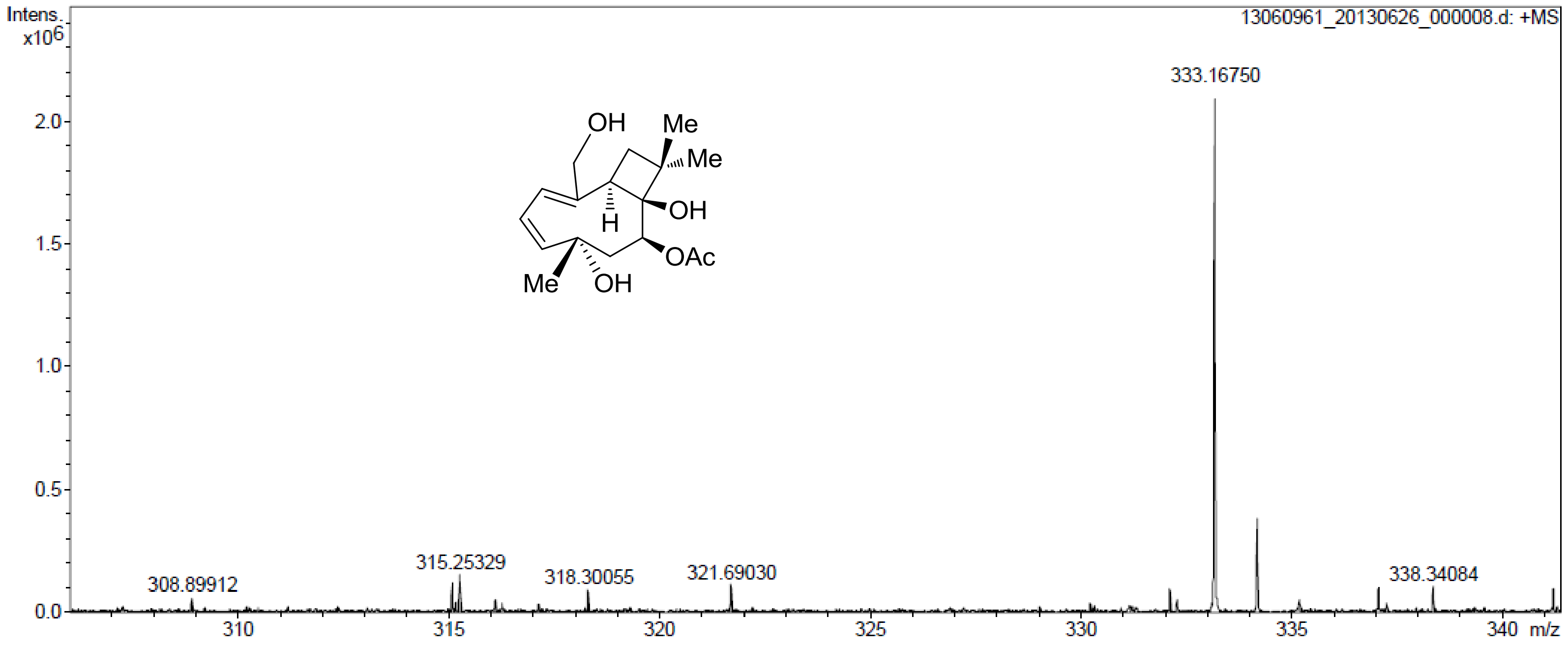

Meas. m/z	#	Formula	Score	m/z	err [mDa]	err [ppm]	mSigma	rdb	e⁻ Conf	N-Rule
333.16750	1	C 17 H 26 Na O 5	100.00	333.16725	-0.3	-0.8	1.0	4.5	even	ok

Figure S4. IR spectrum of punctaporonin H (**1**).

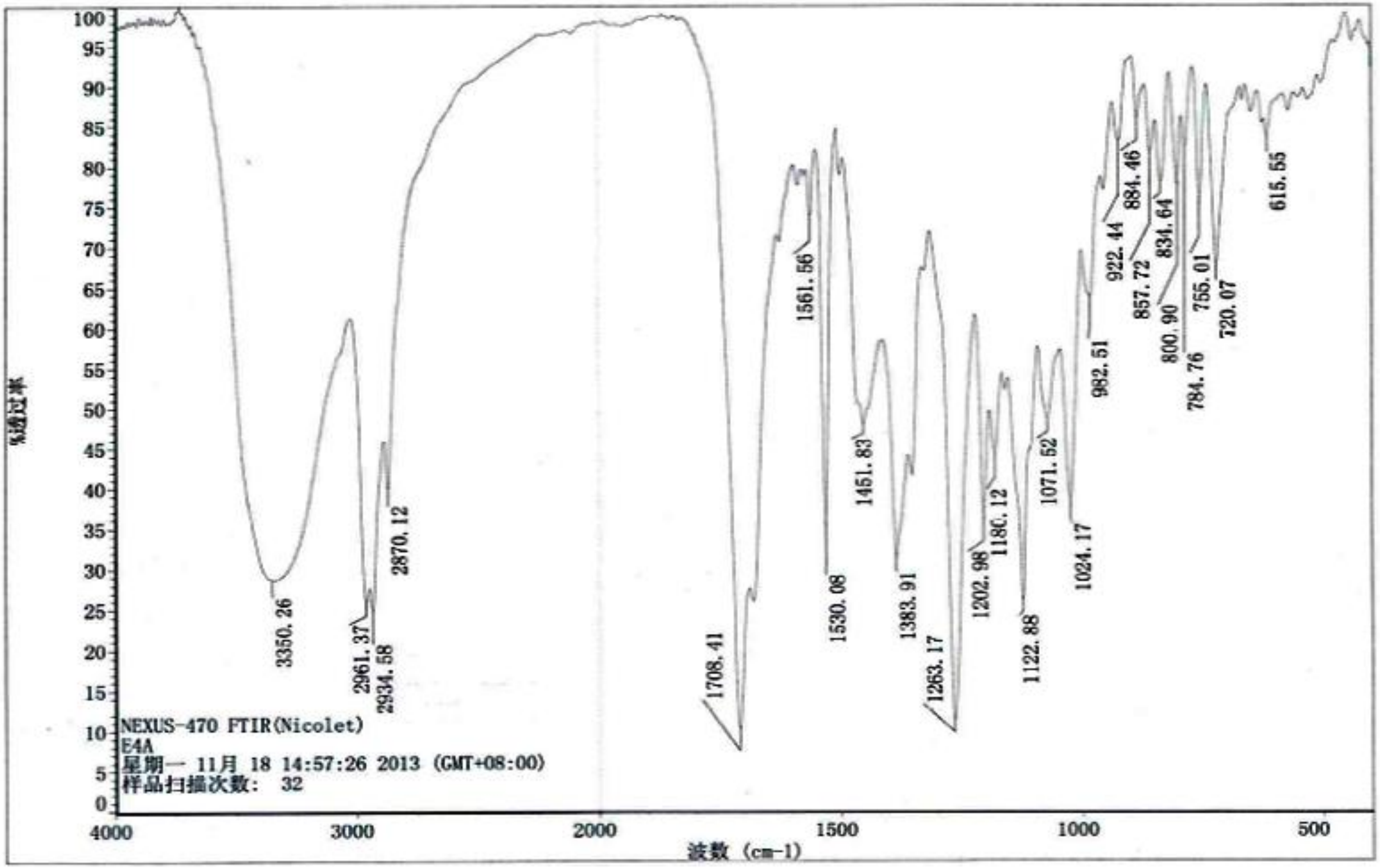

Figure S5. ^{1}H-NMR spectrum of punctaporonin H (**1**).

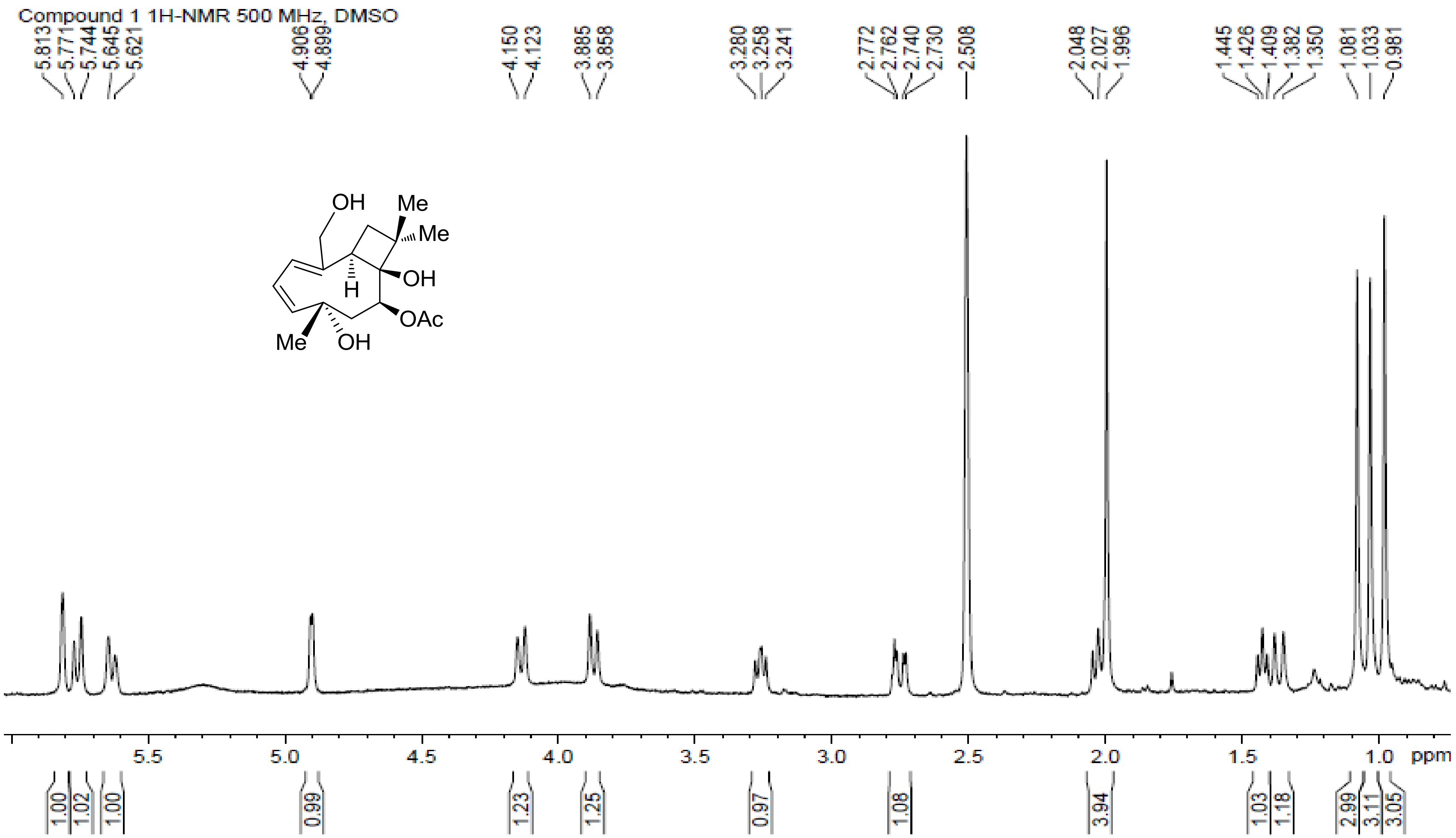

Figure S6. APT spectrum of punctaporonin H (**1**).

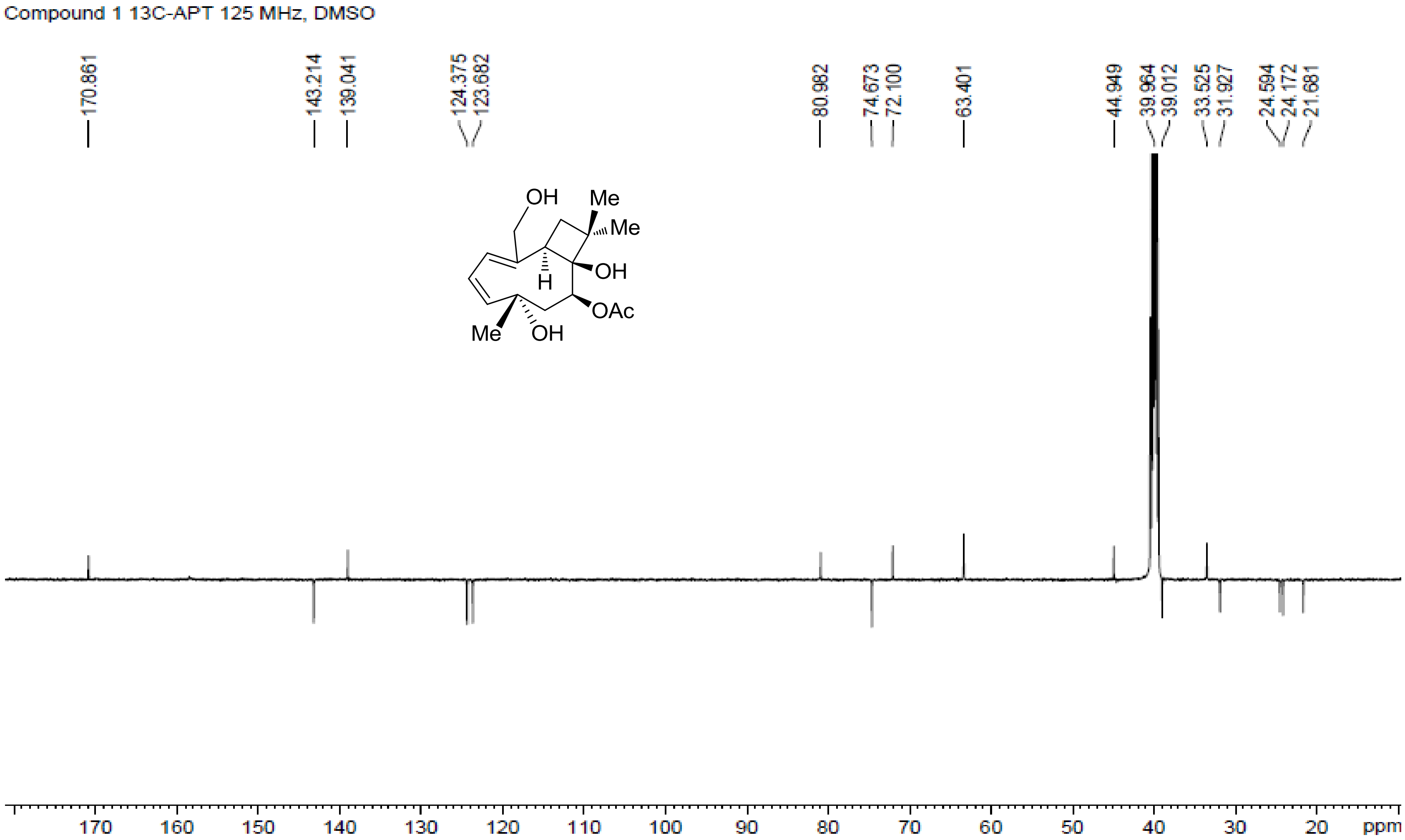

Figure S7. COSY spectrum of punctaporonin H (**1**).

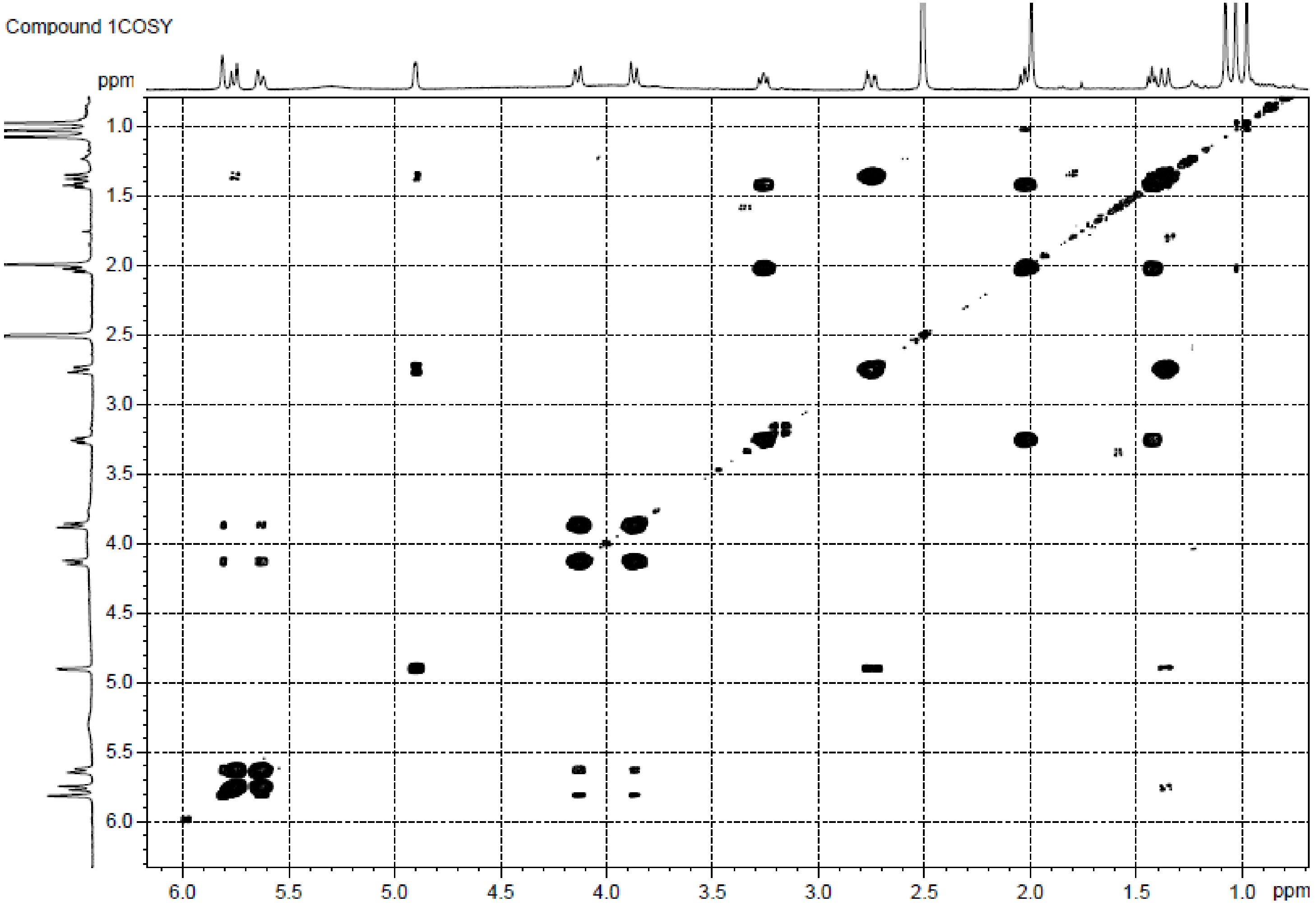

Figure S8. HSQC spectrum of punctaporonin H (**1**).

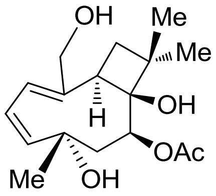

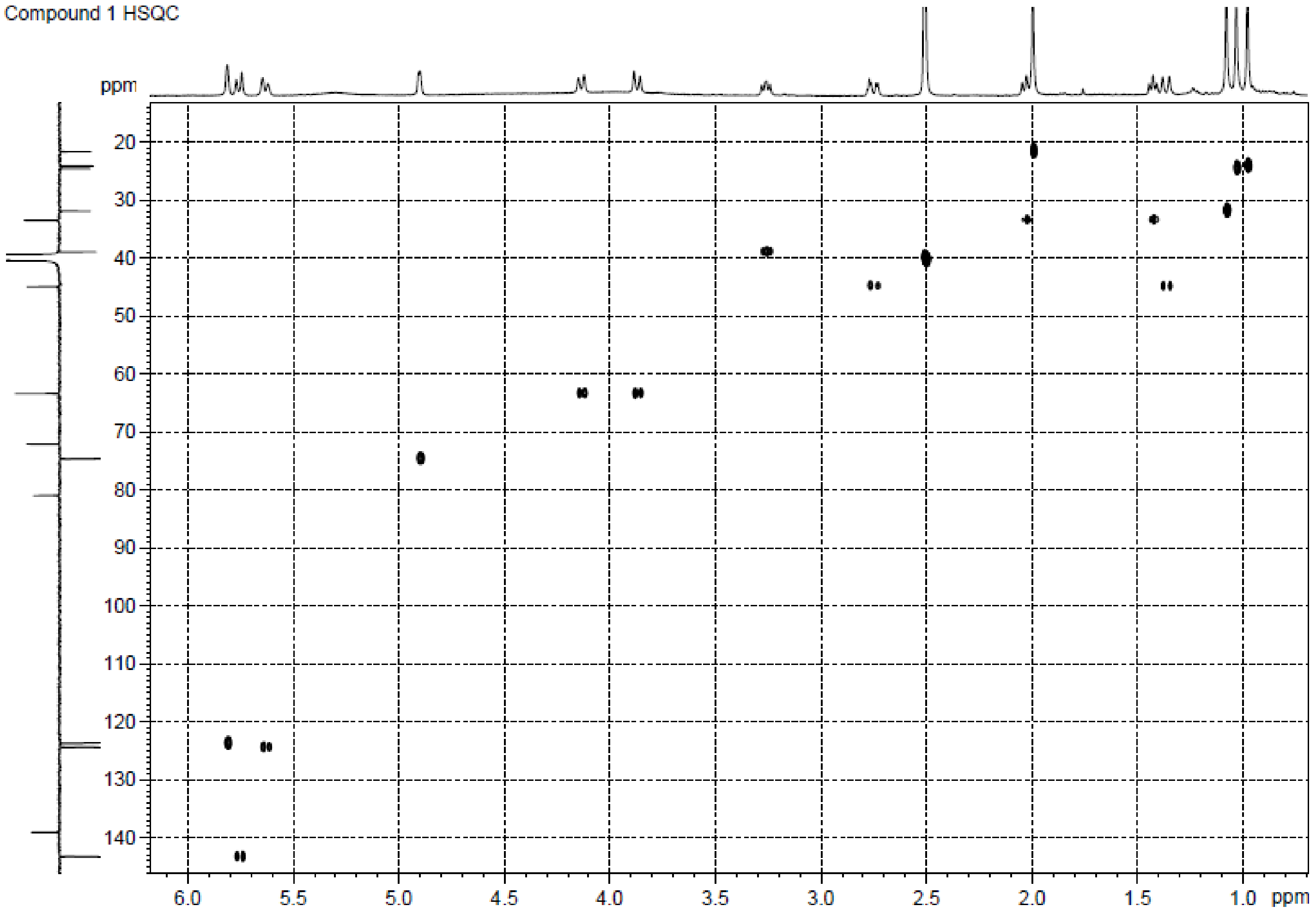

Figure S9. HMBC spectrum of punctaporonin H (**1**).

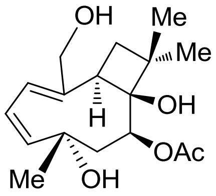

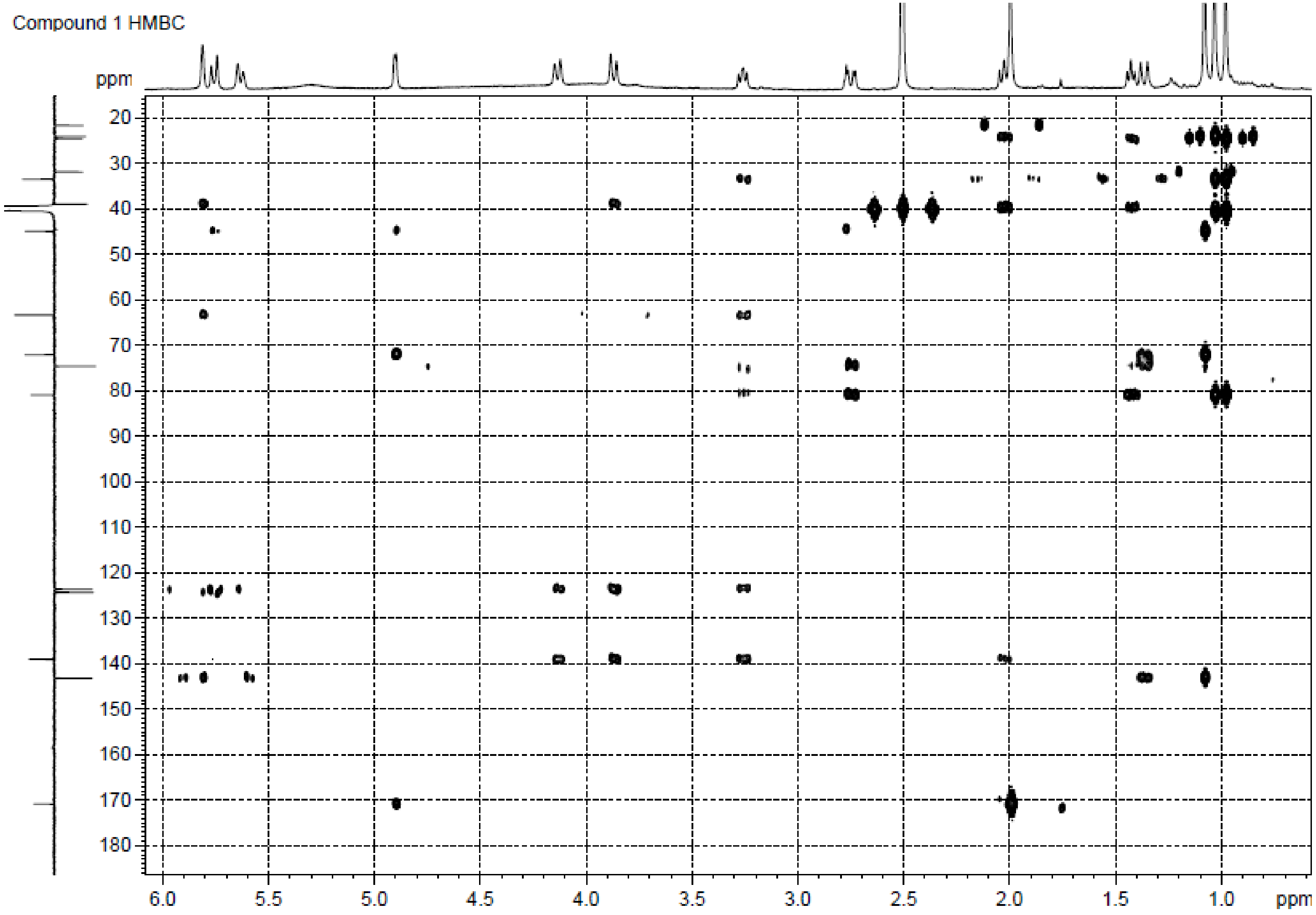

Figure S10. NOESY spectrum of punctaporonin H (**1**).

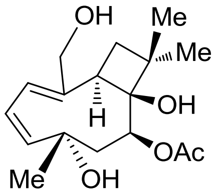

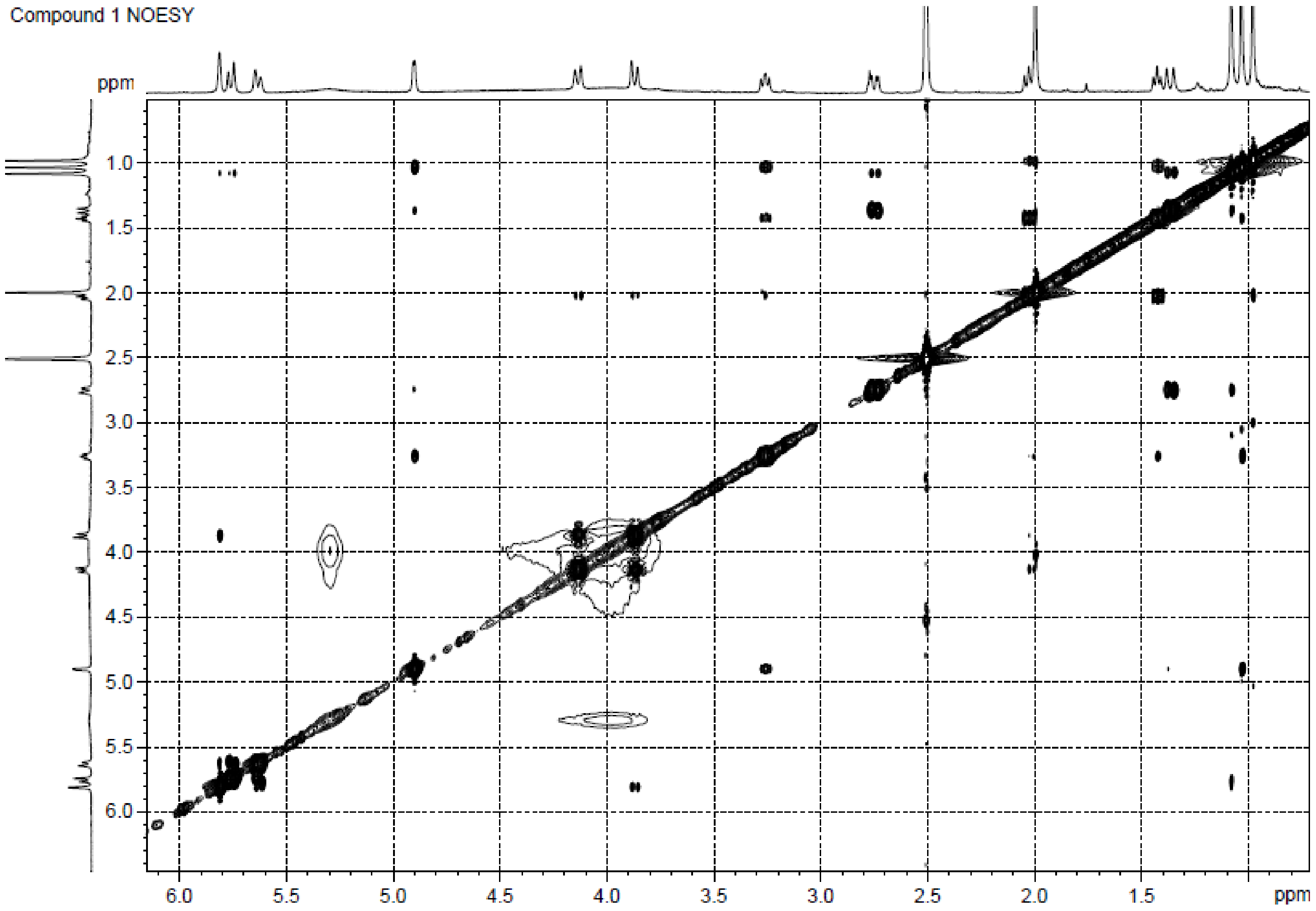

4. The HRESIMS, IR and NMR Data of Punctaporonin I (2)

Figure S11. HRESIMS spectrum of punctaporonin I (**2**).

Peking University Mass Spectrometry Sample Analysis Report

Analysis Info

Analysis Name	13060961_20130626_000010.d	Acquisition Date	6/26/2013 3:55:28 PM
Sample	10	Instrument	Bruker Apex IV FTMS
Comment	ESI Positive	Operator	Peking University

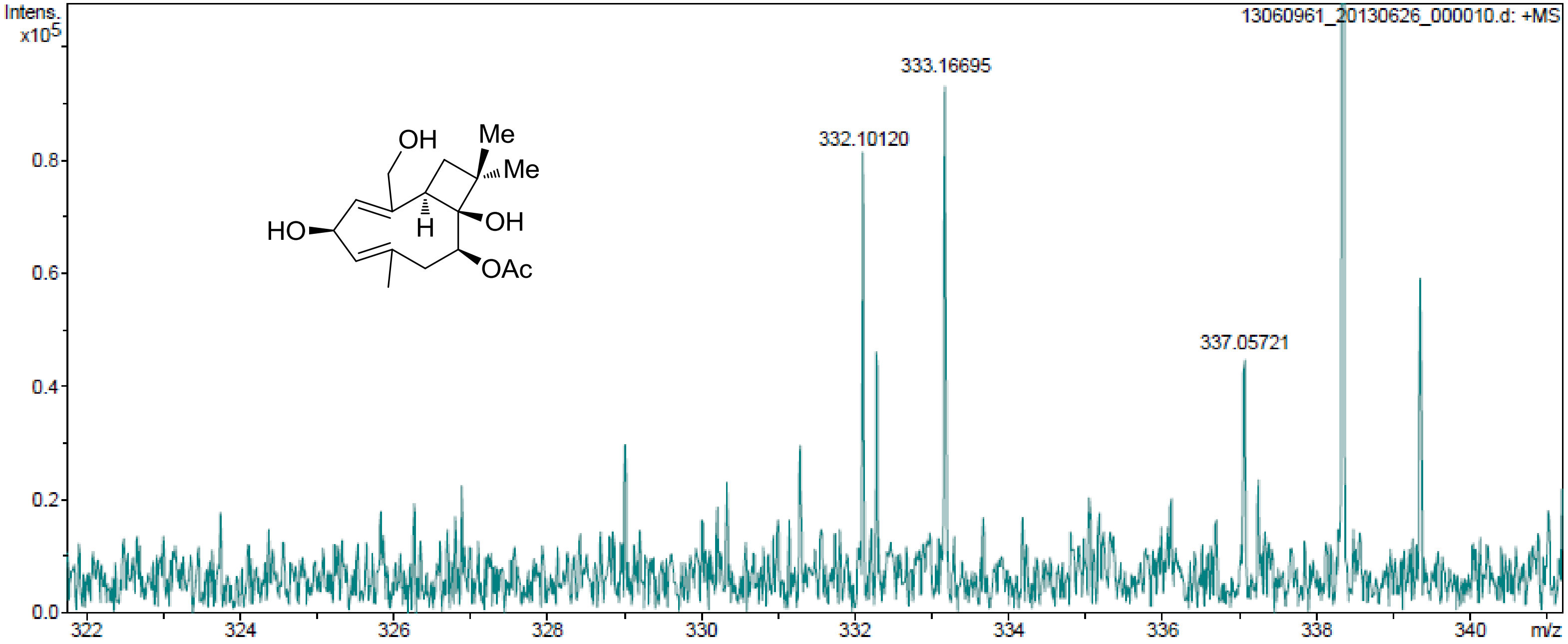

Meas. m/z	#	Formula	Score	m/z	err [mDa]	err [ppm]	mSigma	rdb	e⁻ Conf	N-Rule
333.16695	1	C 17 H 26 Na O 5	100.00	333.16725	0.3	0.9	107.2	4.5	even	ok

Figure S12. IR spectrum of punctaporonin I (**2**).

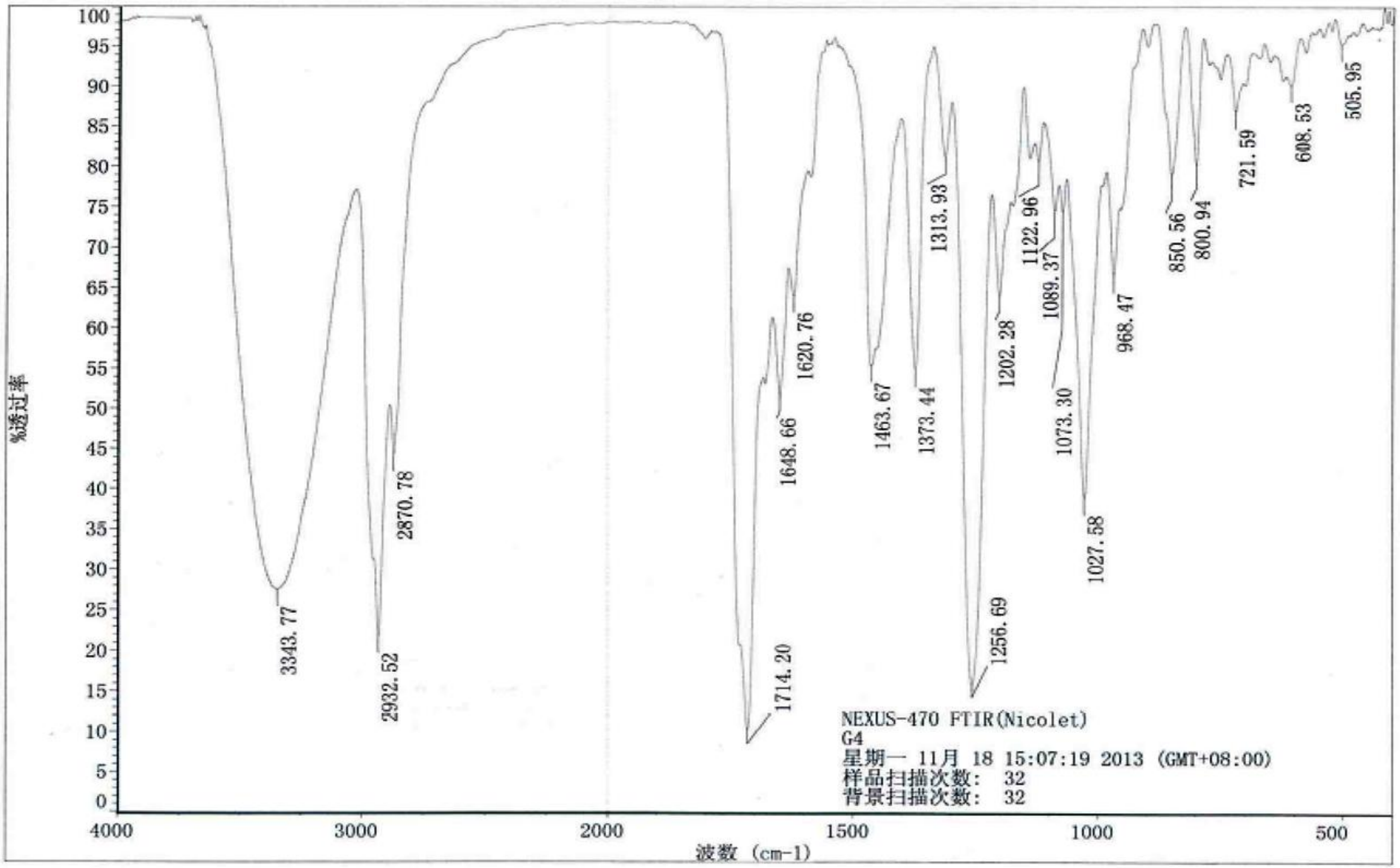

Figure S13. ^{1}H-NMR spectrum of punctaporonin I (**2**).

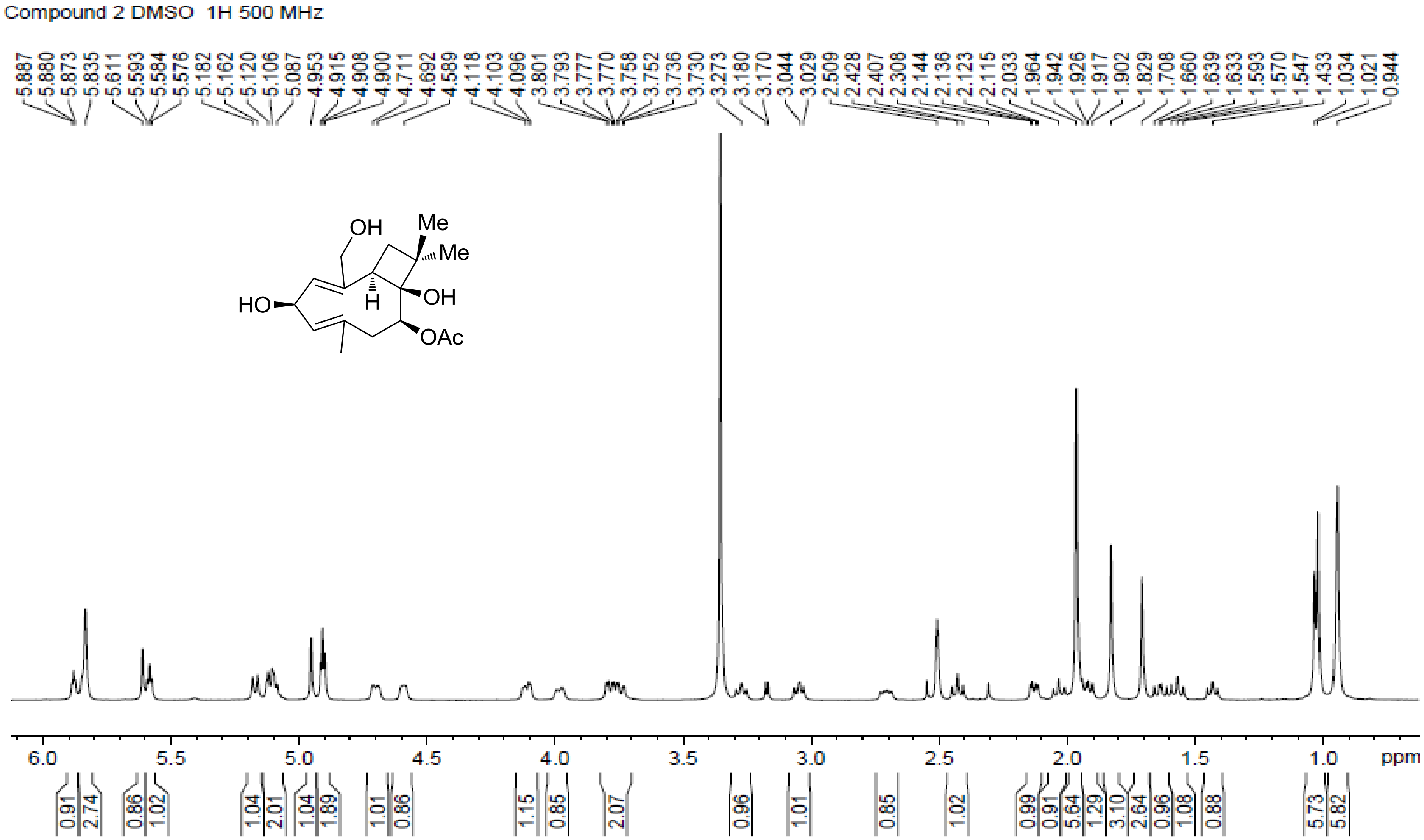

Figure S14. APT spectrum of punctaporonin I (**2**).

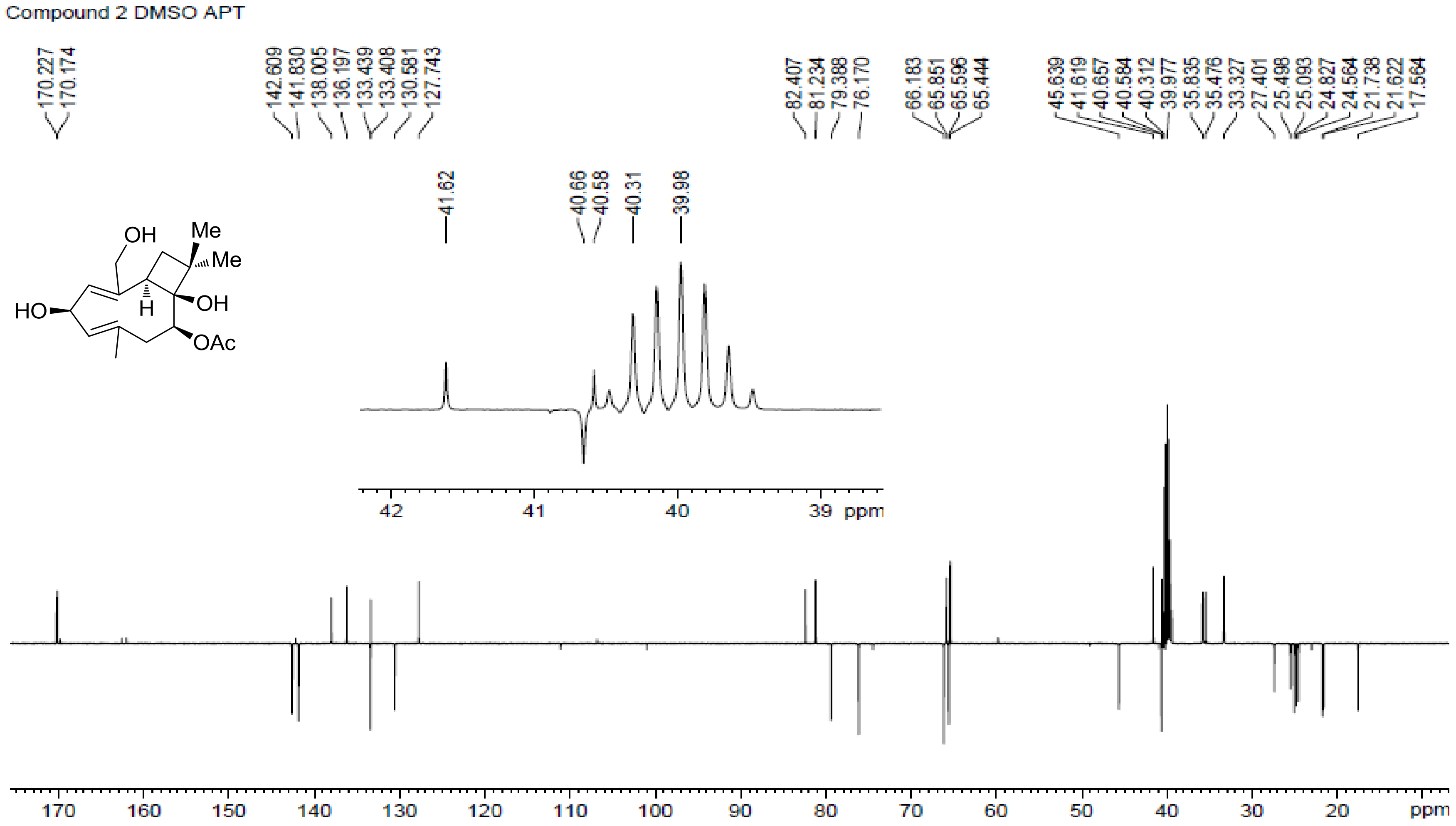

Figure S15. COSY spectrum of punctaporonin I (**2**).

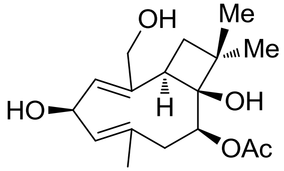

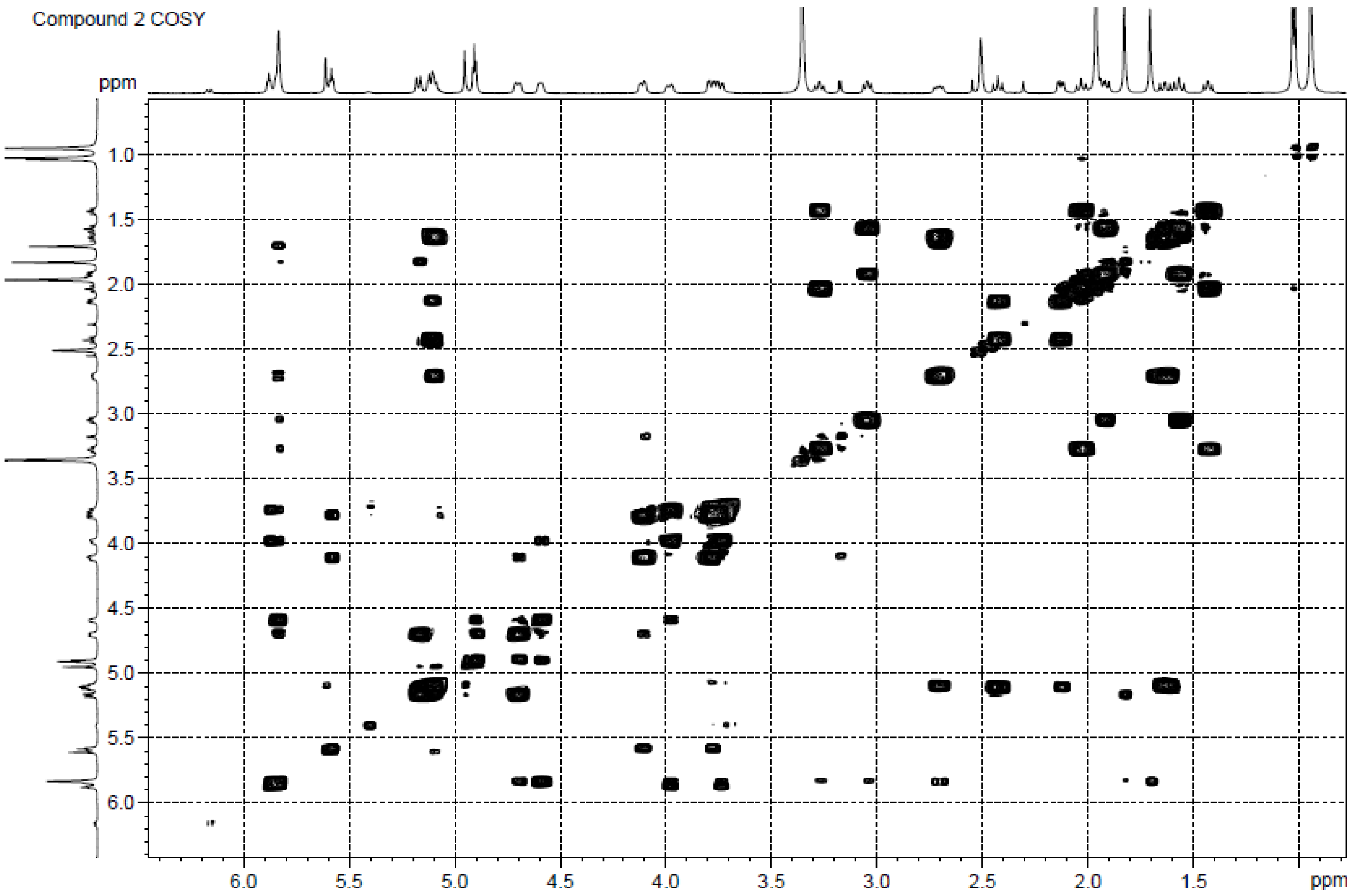

Figure S16. HSQC spectrum of punctaporonin I (**2**).

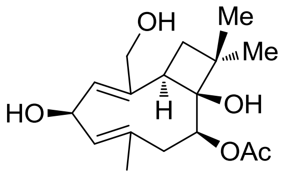

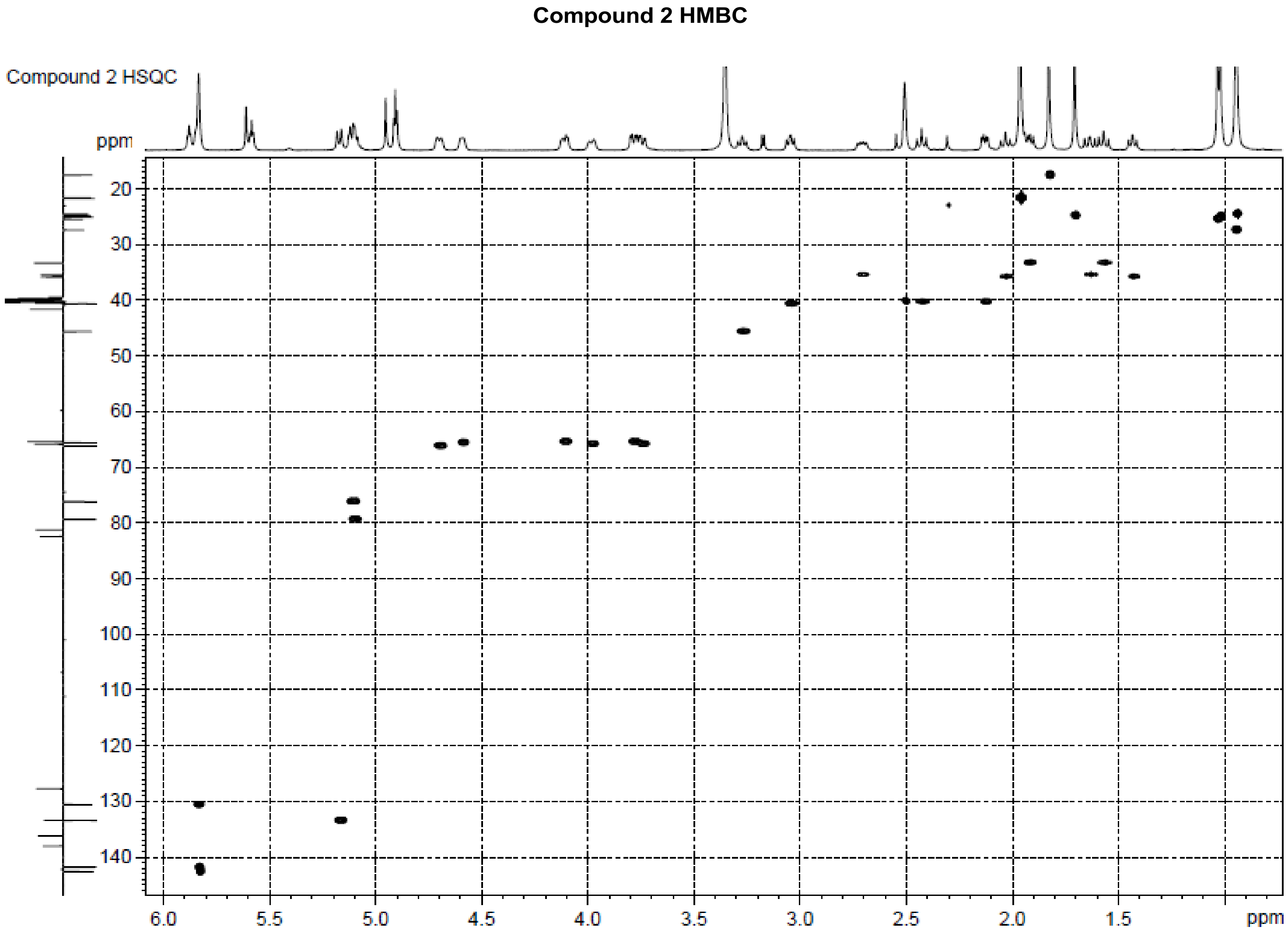

Figure S17. HMBC spectrum of punctaporonin I (**2**).

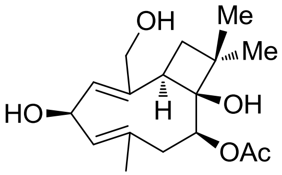

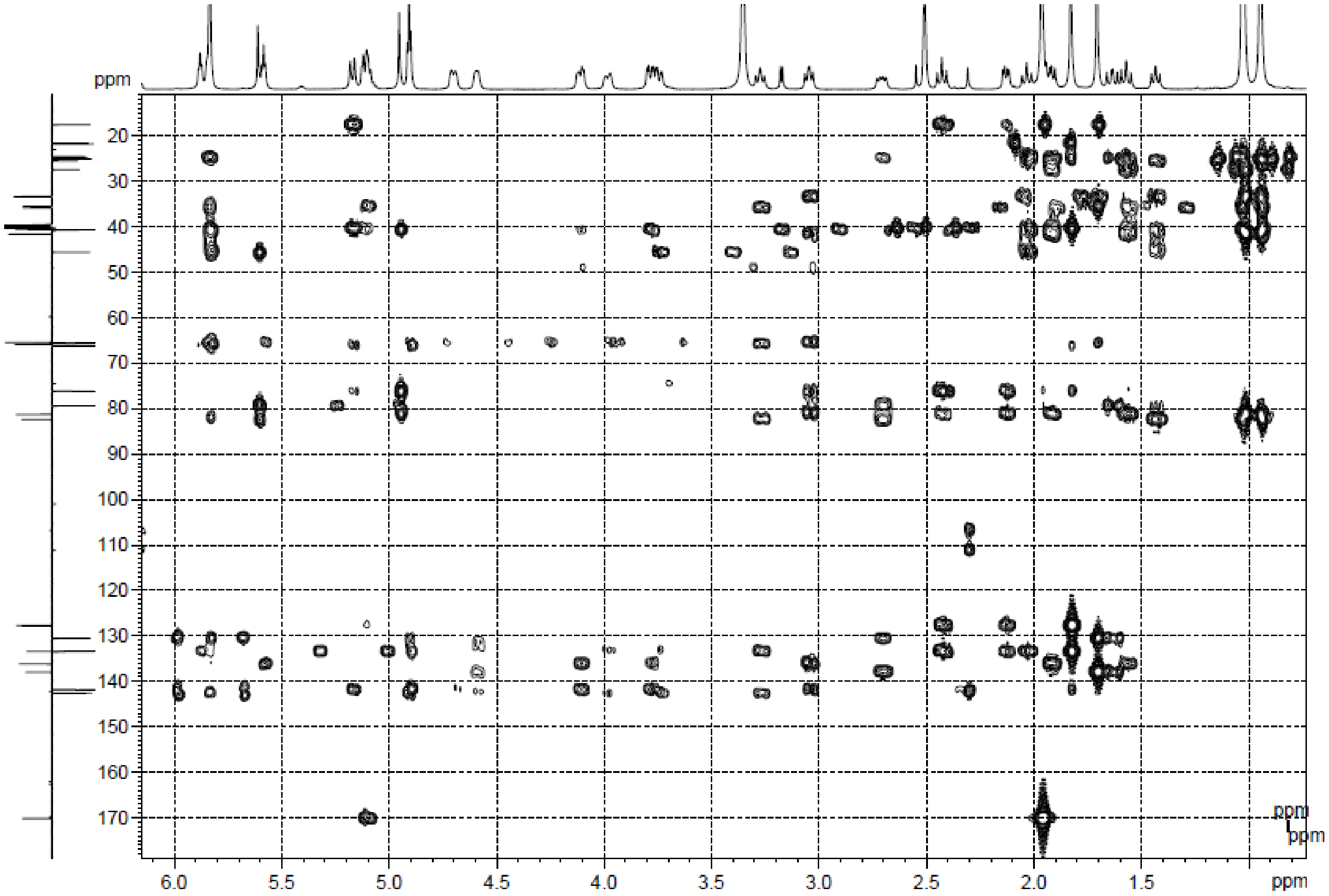

Figure S18. NOESY spectrum of punctaporonin I (**2**).

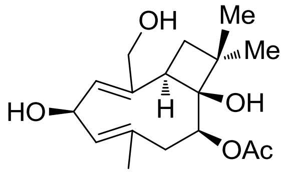

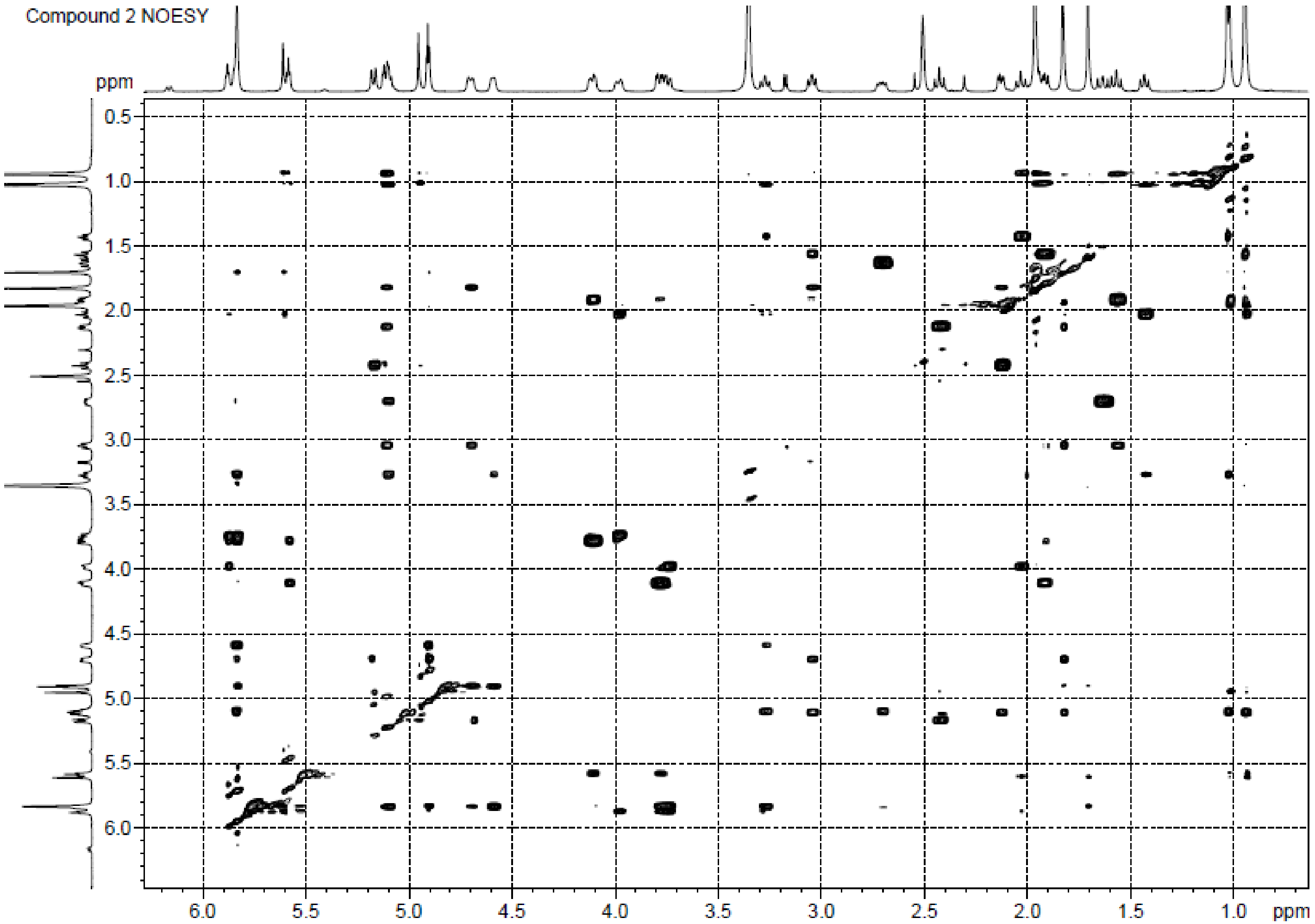

5. The HRESIMS, IR and NMR Data of Punctaporonin J (3)

Figure S19. HRESIMS spectrum of punctaporonin J (**3**).

Peking University Mass Spectrometry Sample Analysis Report

Analysis Info

Analysis Name	13060961_20130626_000011.d	Acquisition Date	6/26/2013 4:28:01 PM
Sample	11	Instrument	Bruker Apex IV FTMS
Comment	ESI Positive	Operator	Peking University

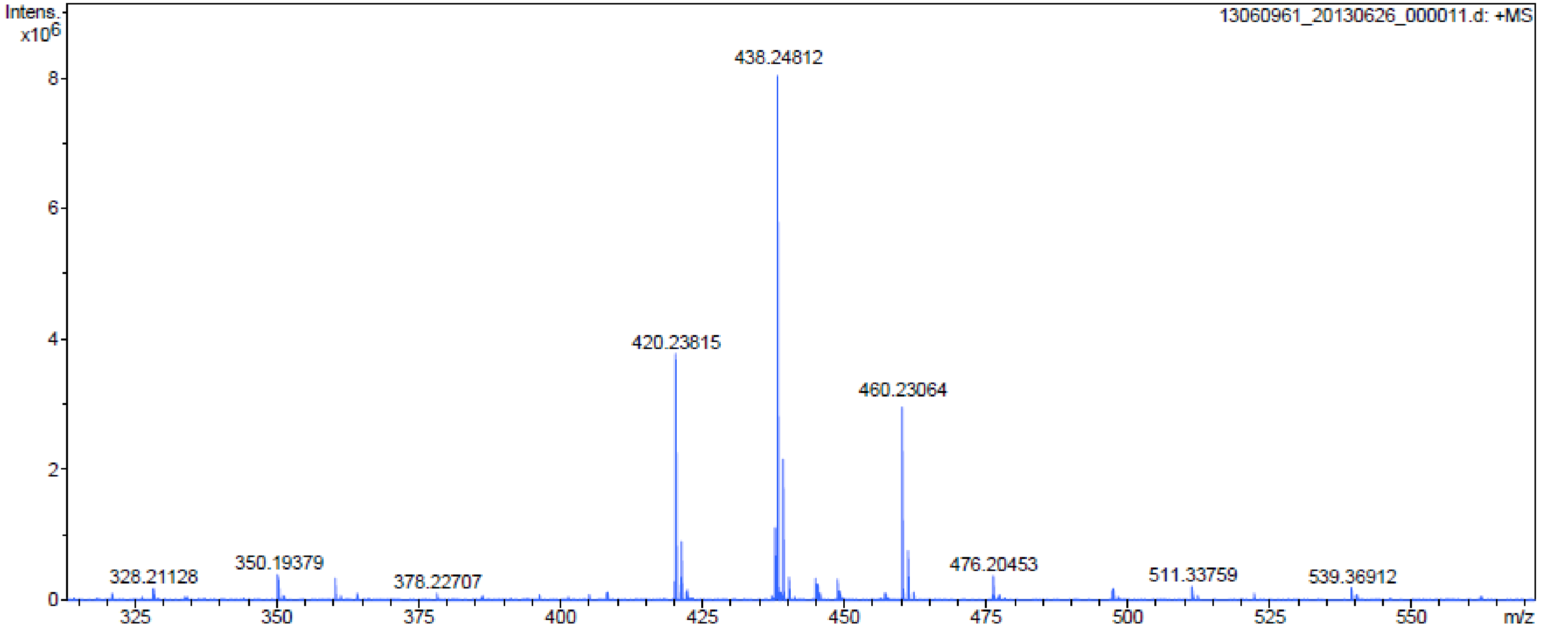

Meas. m/z	#	Formula	Score	m/z	err [mDa]	err [ppm]	mSigma	rdb	e⁻ Conf	N-Rule
438.24812	1	C 23 H 36 N O 7	100.00	438.24863	0.5	1.2	7.6	6.5	even	ok

Figure S20. IR spectrum of punctaporonin J (**3**).

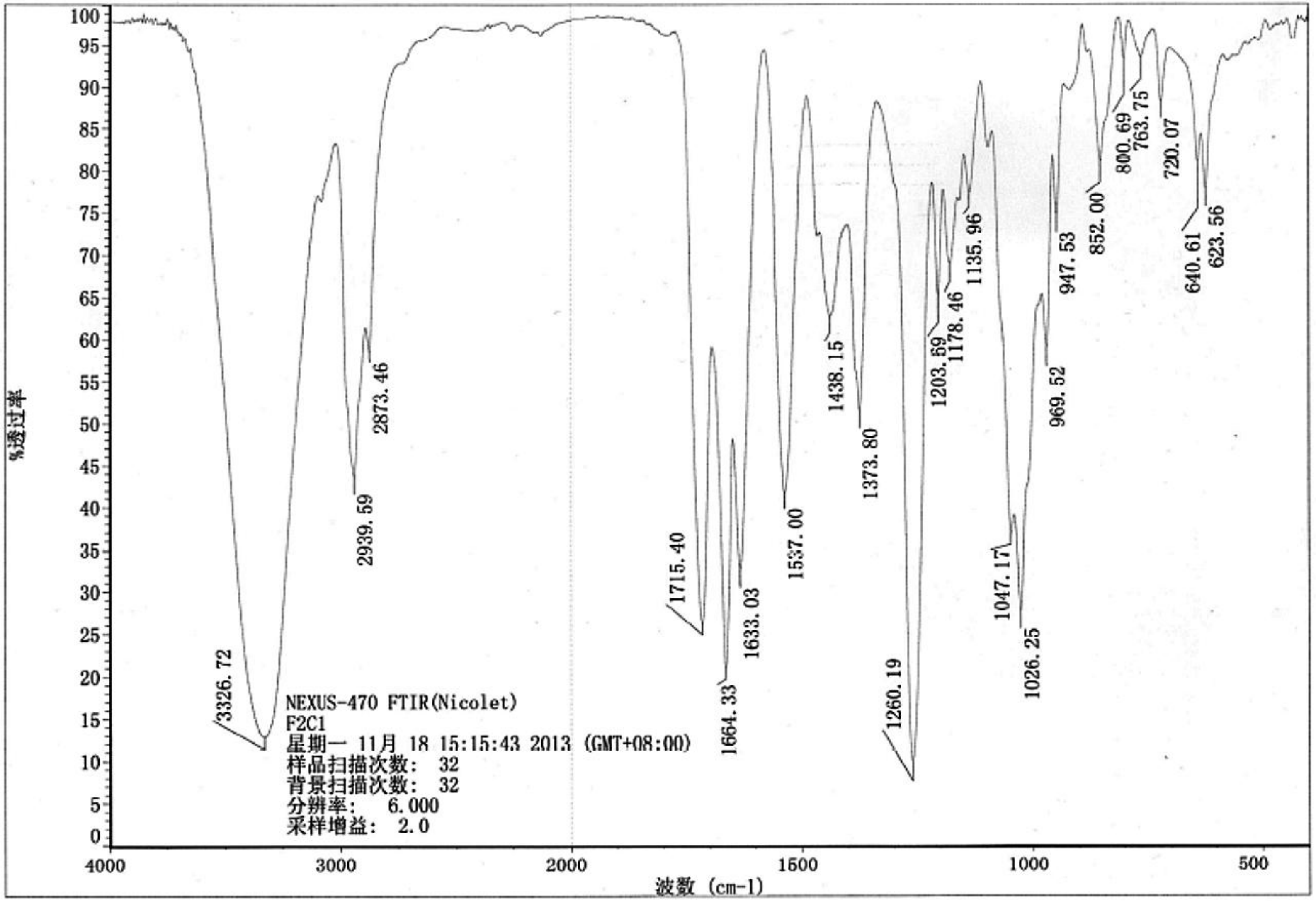

Figure S21. ^{1}H-NMR spectrum of punctaporonin J (**3**).

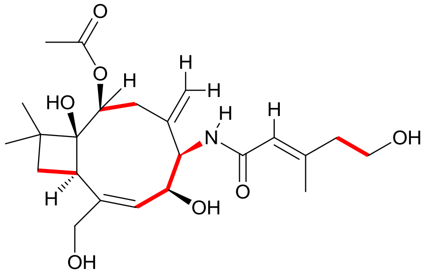

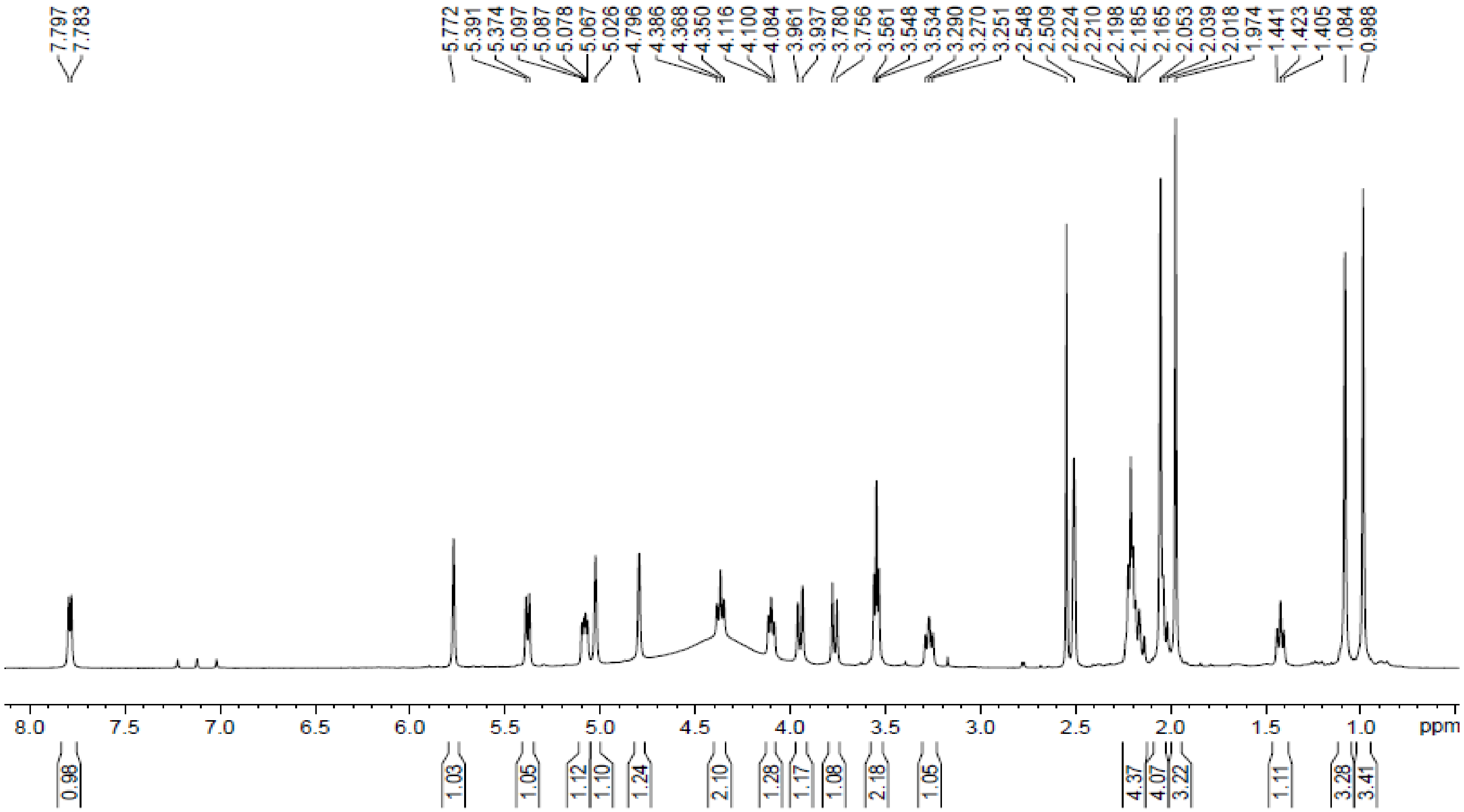

Figure S22. APT spectrum of punctaporonin J (**3**).

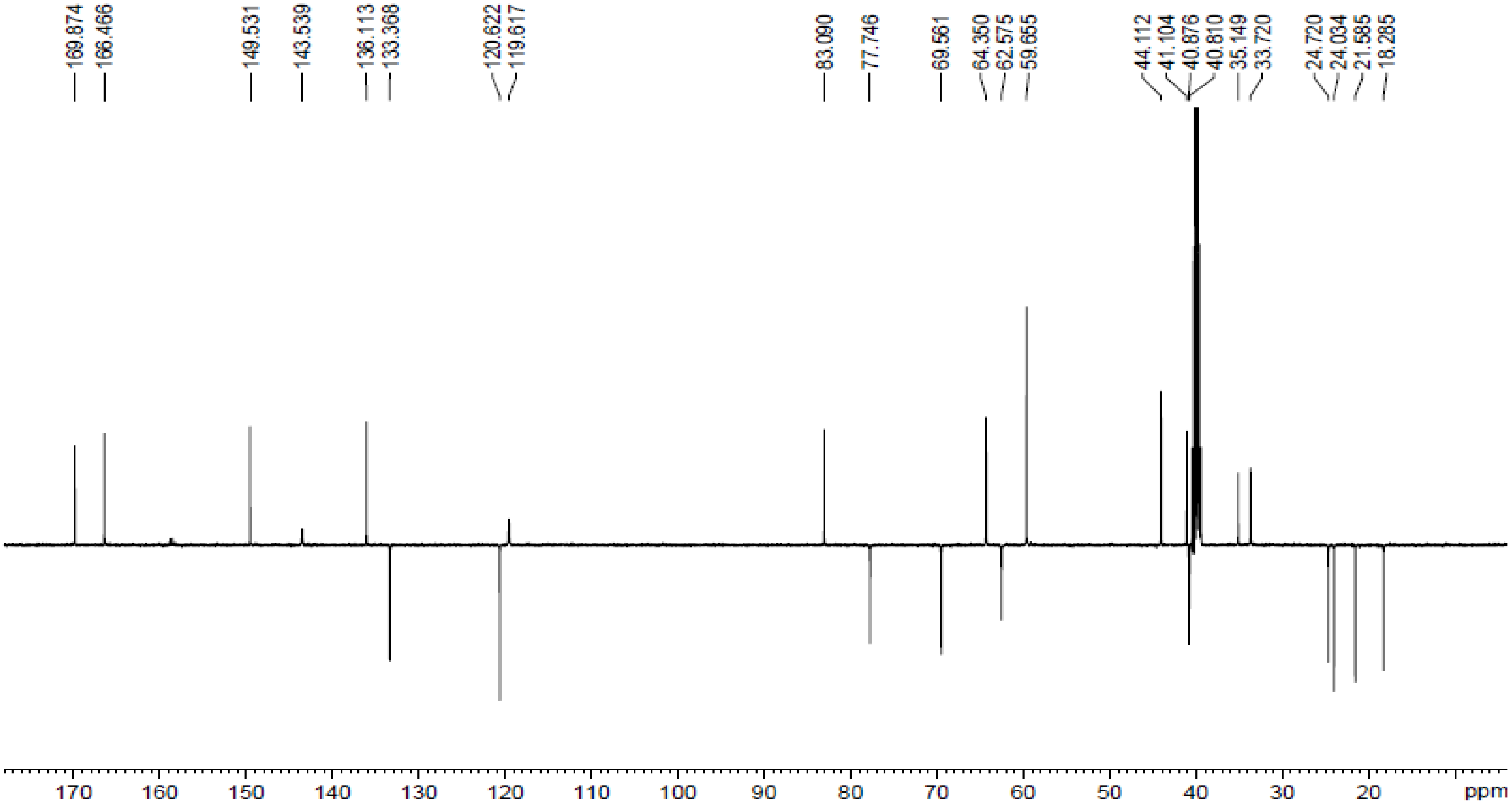

Figure S23. COSY spectrum of punctaporonin J (**3**).

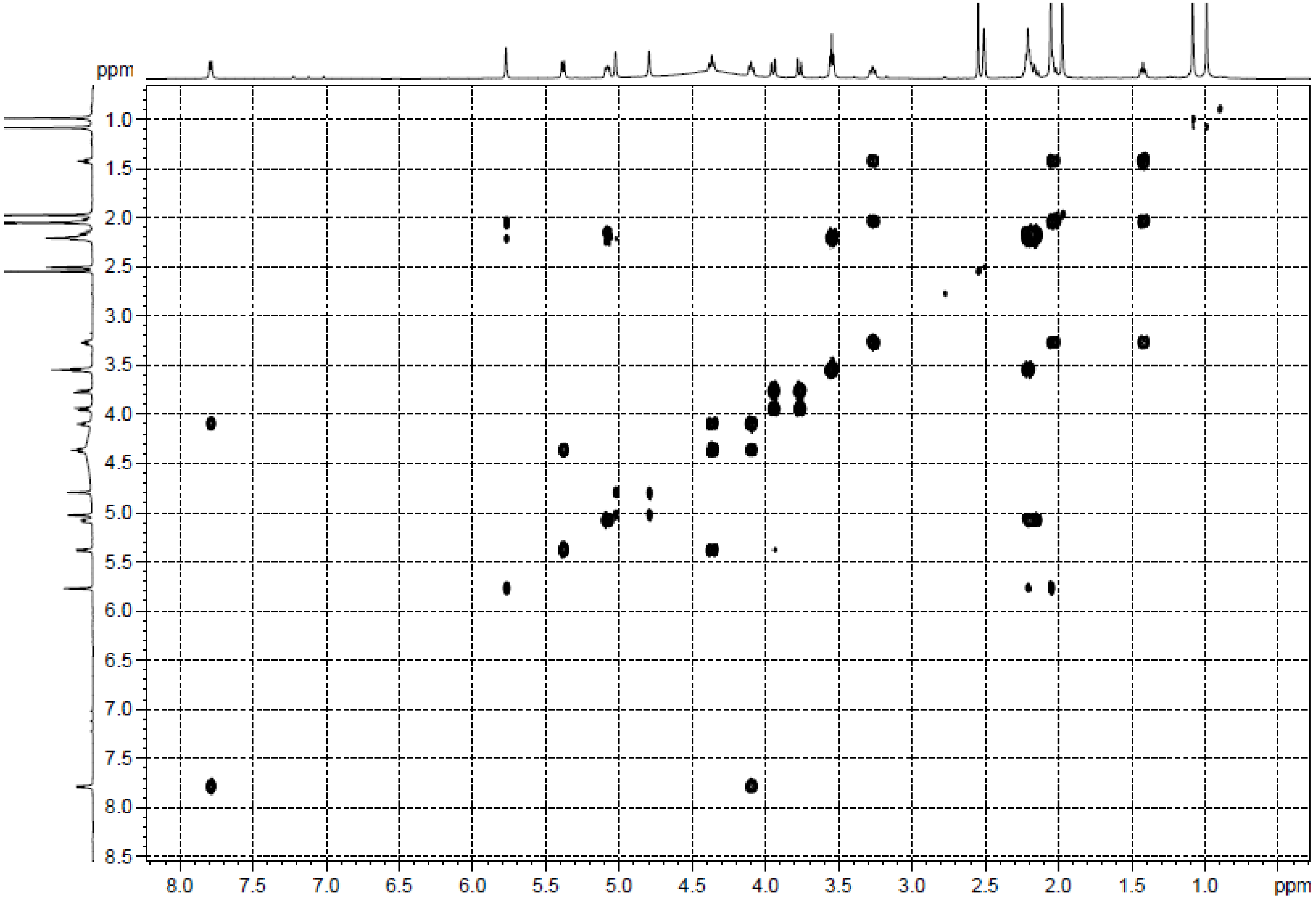

Figure S24. HSQC spectrum of punctaporonin J (**3**).

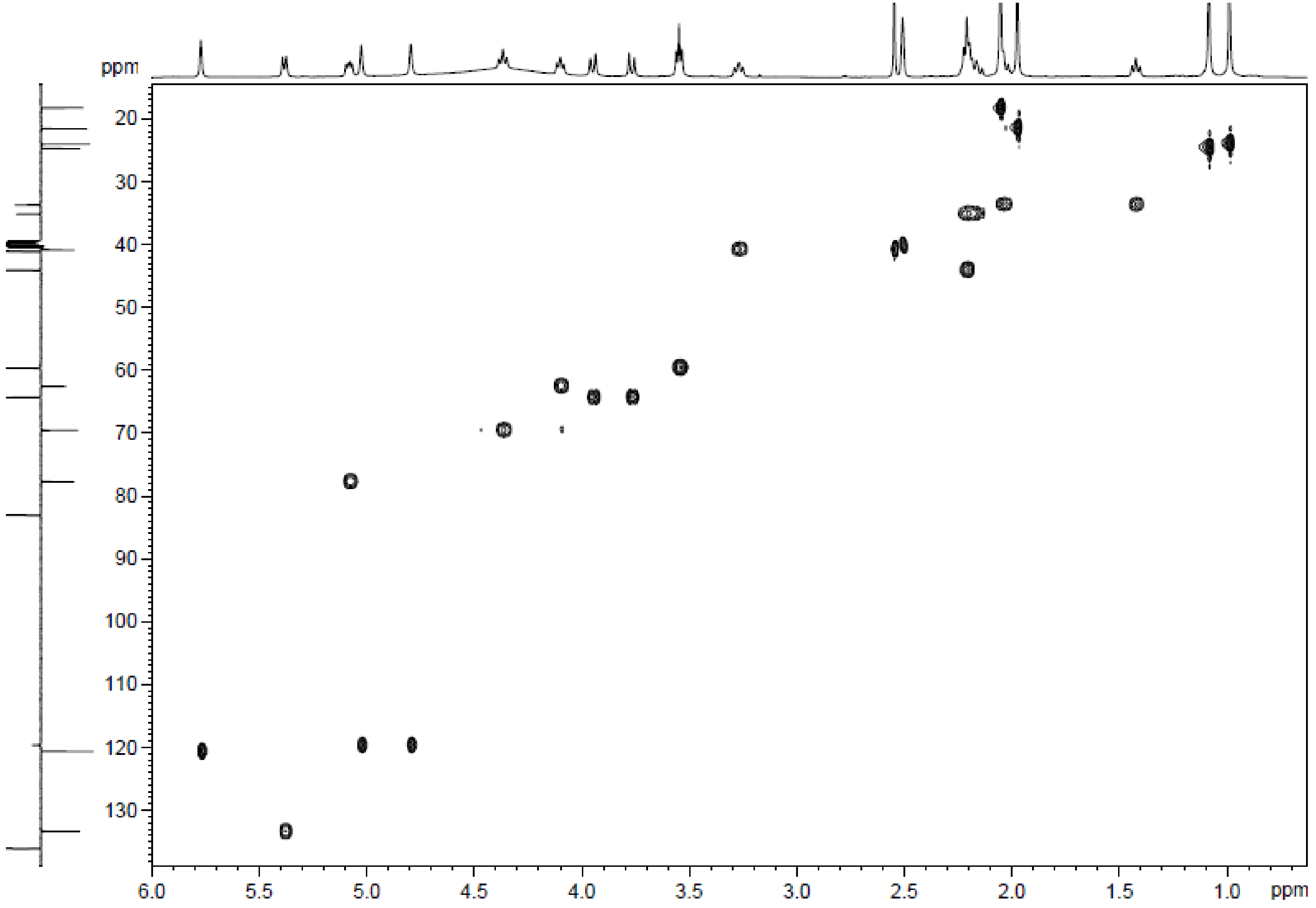

Figure S25. HMBC spectrum of punctaporonin J (**3**).

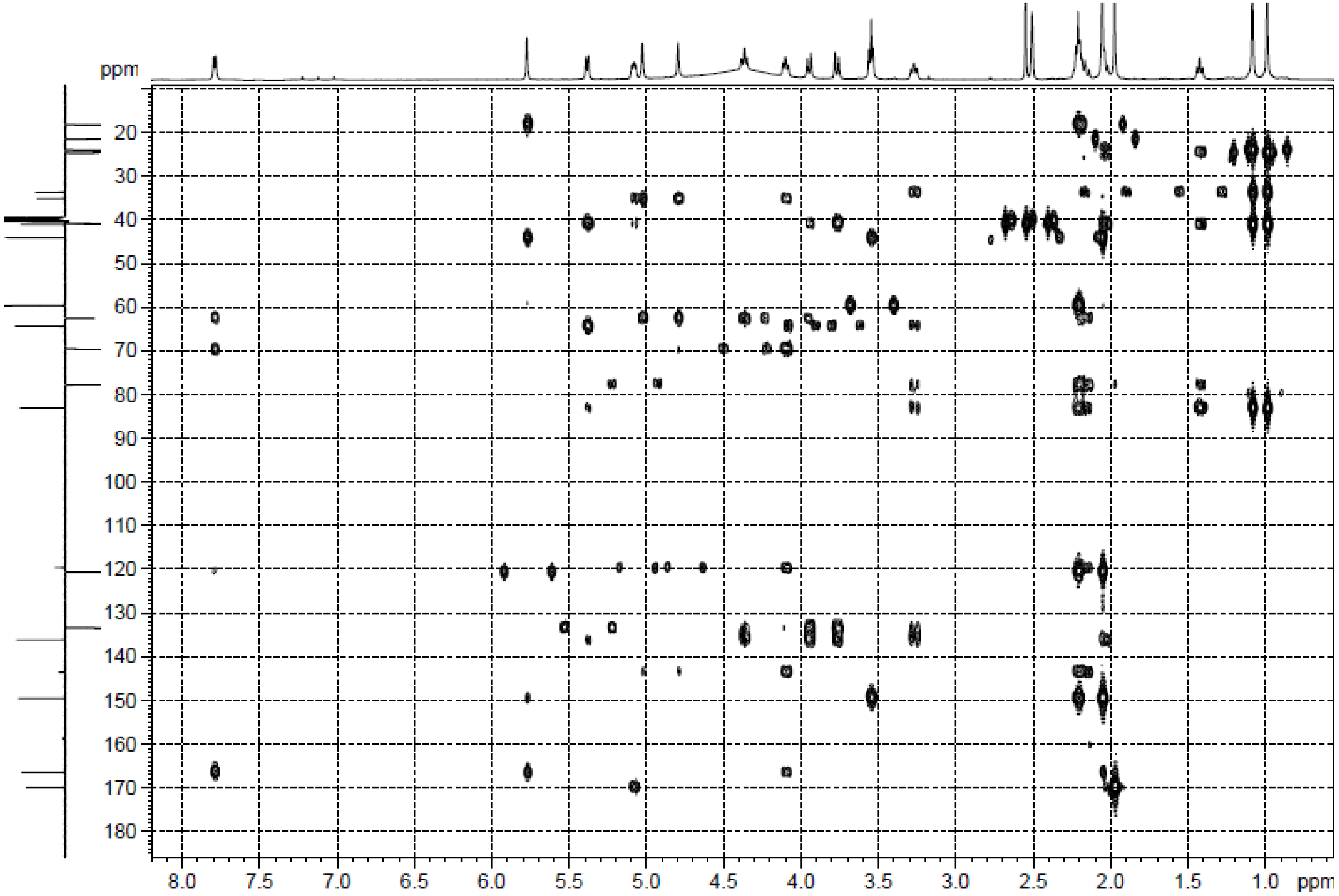

Figure S26. ROESY spectrum of punctaporonin J (**3**).

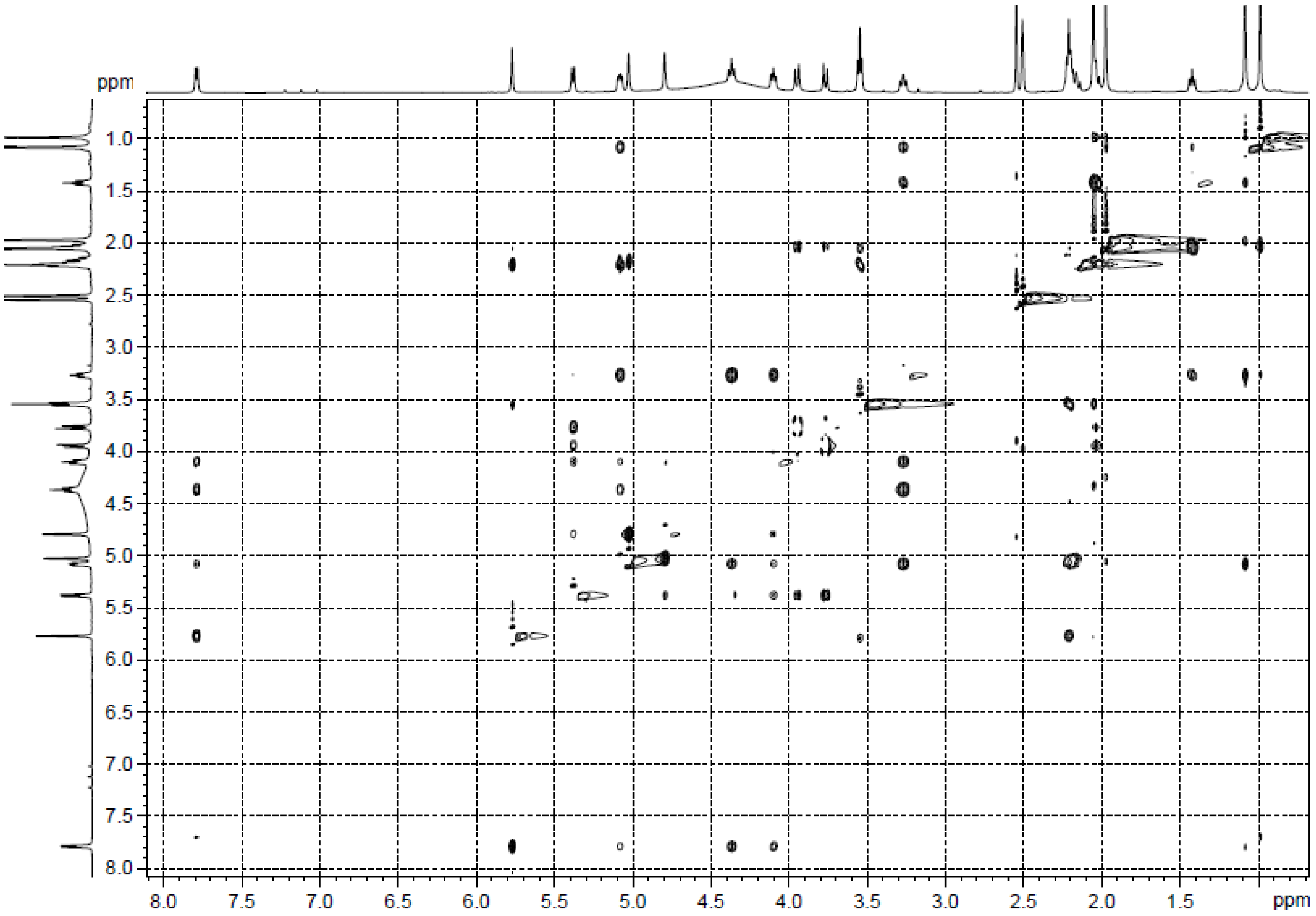

6. The HRESIMS, IR and NMR Data of Punctaporonin K (4)

Figure S27. HRESIMS spectrum of punctaporonin K (**4**).

Peking University Mass Spectrometry Sample Analysis Report

Analysis Info

Analysis Name	13060961_20130626_000012.d	Acquisition Date	6/26/2013 4:30:34 PM
Sample	12	Instrument	Bruker Apex IV FTMS
Comment	ESI Positive	Operator	Peking University

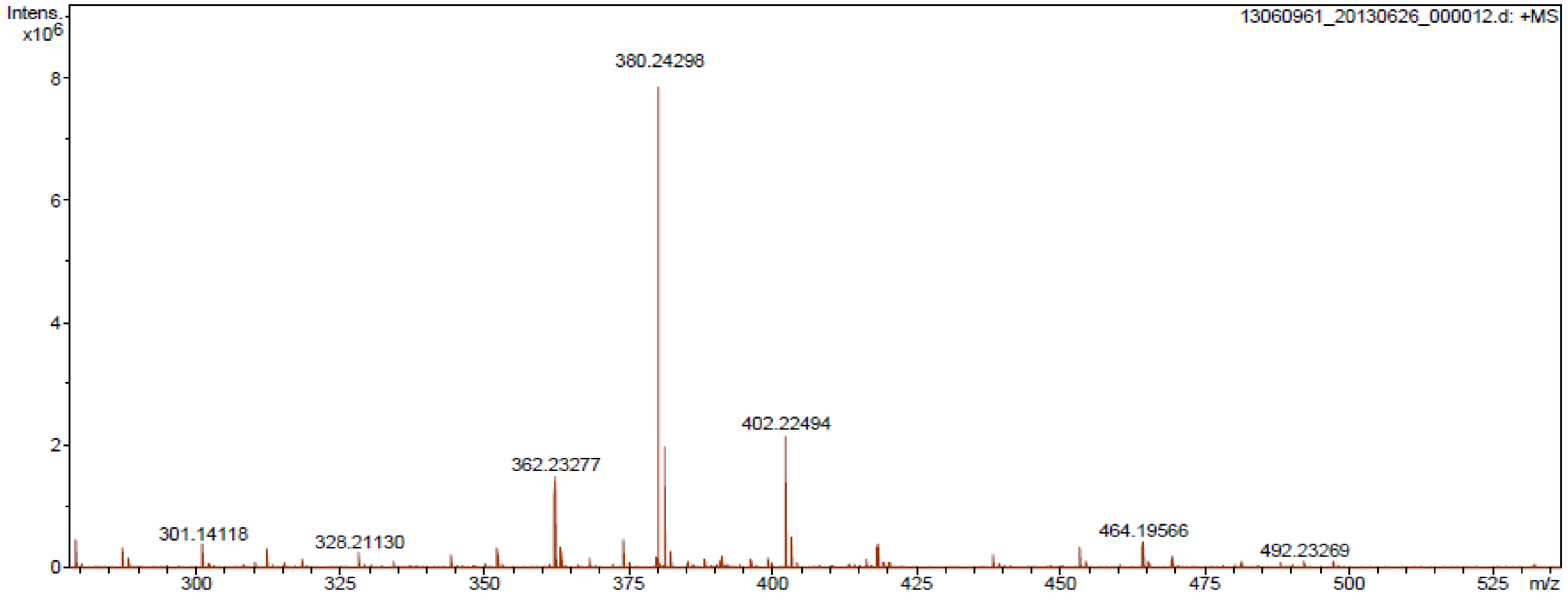

Meas. m/z	#	Formula	Score	m/z	err [mDa]	err [ppm]	mSigma	rdb	e⁻ Conf	N-Rule
380.24298	1	C 21 H 34 N O 5	100.00	380.24315	0.2	0.5	6.1	5.5	even	ok

Figure S28. IR spectrum of punctaporonin K (**4**).

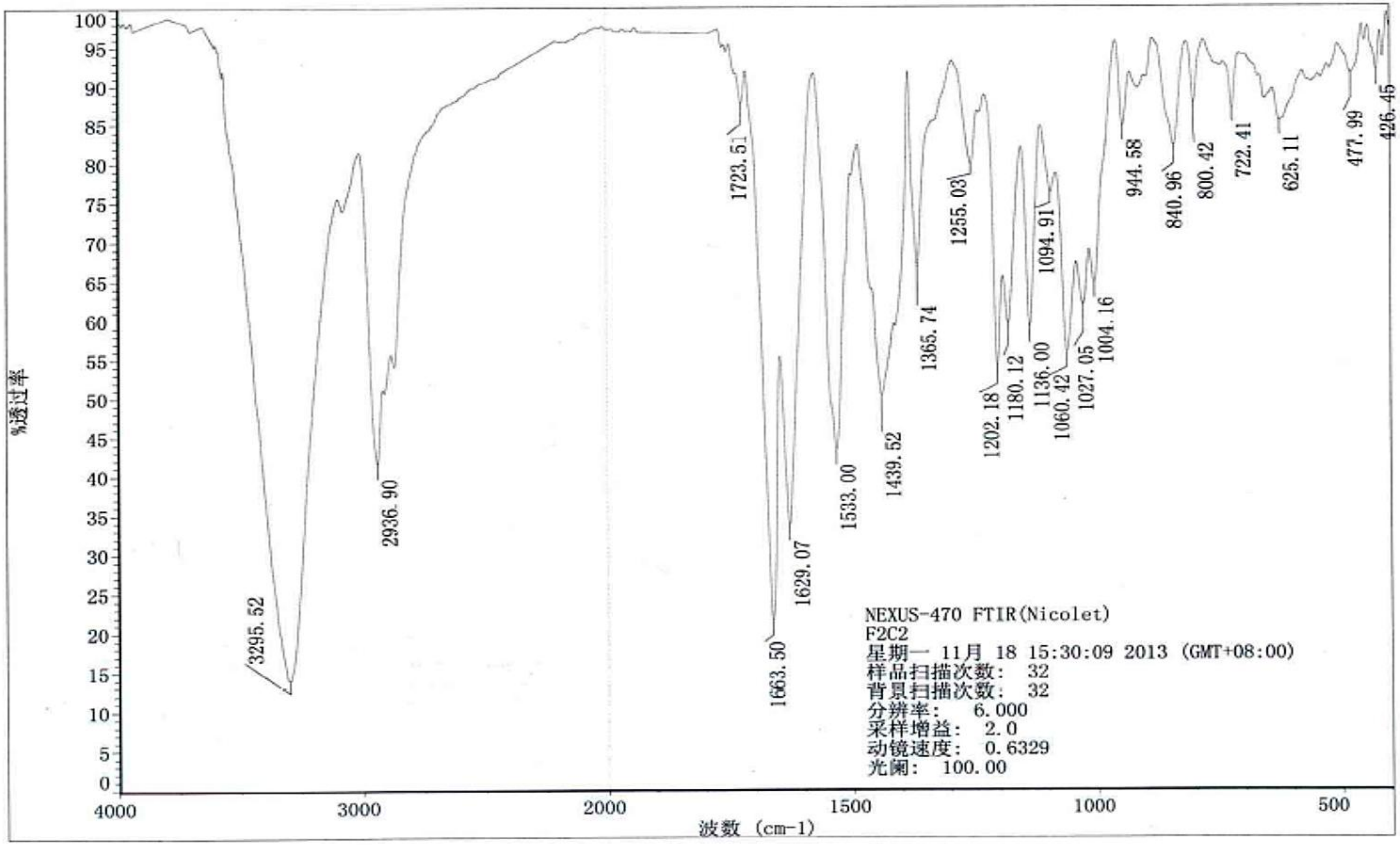

Figure S29. ¹H-NMR spectrum of punctaporonin K (**4**).

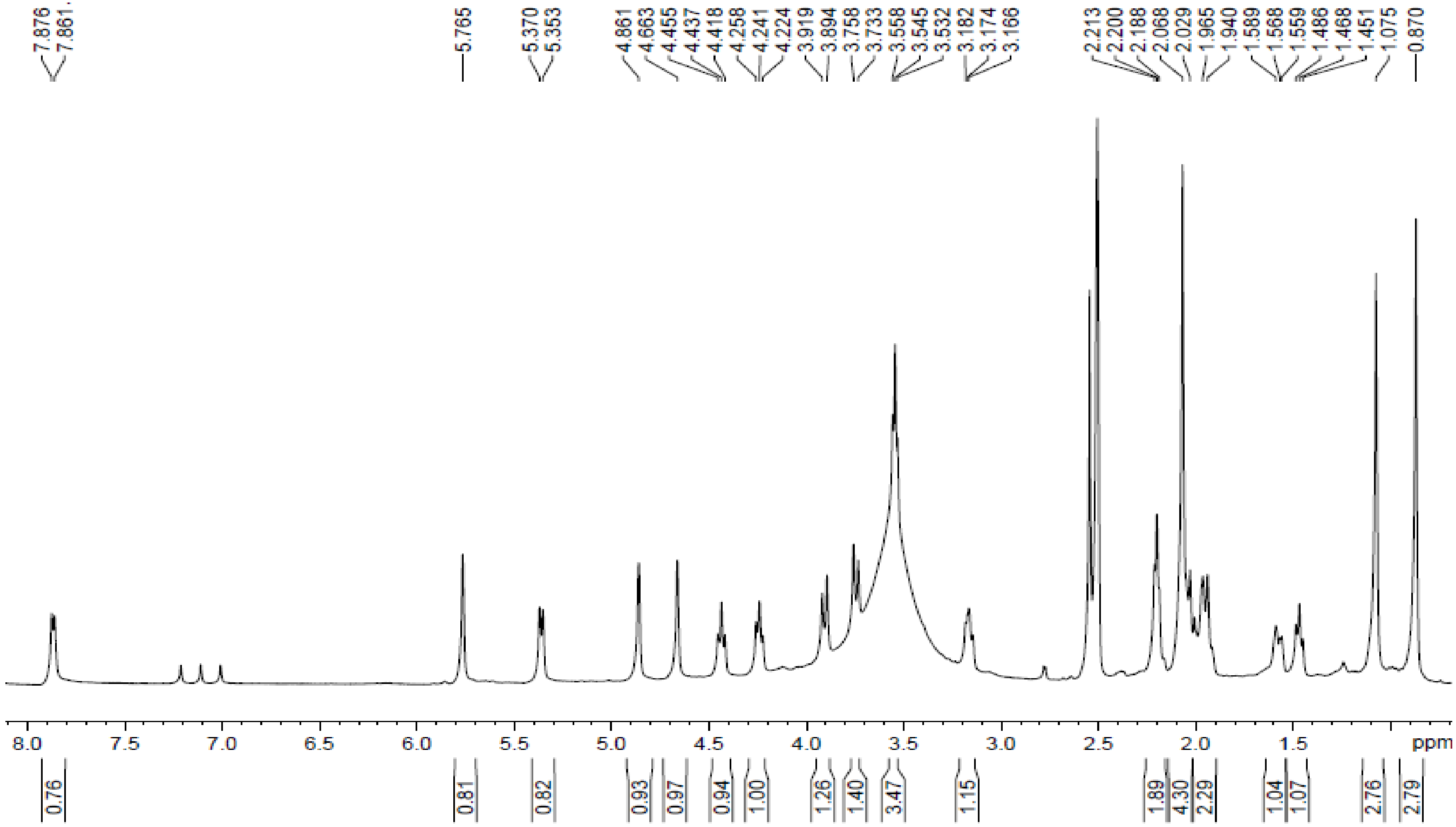

Figure S30. APT spectrum of punctaporonin K (**4**).

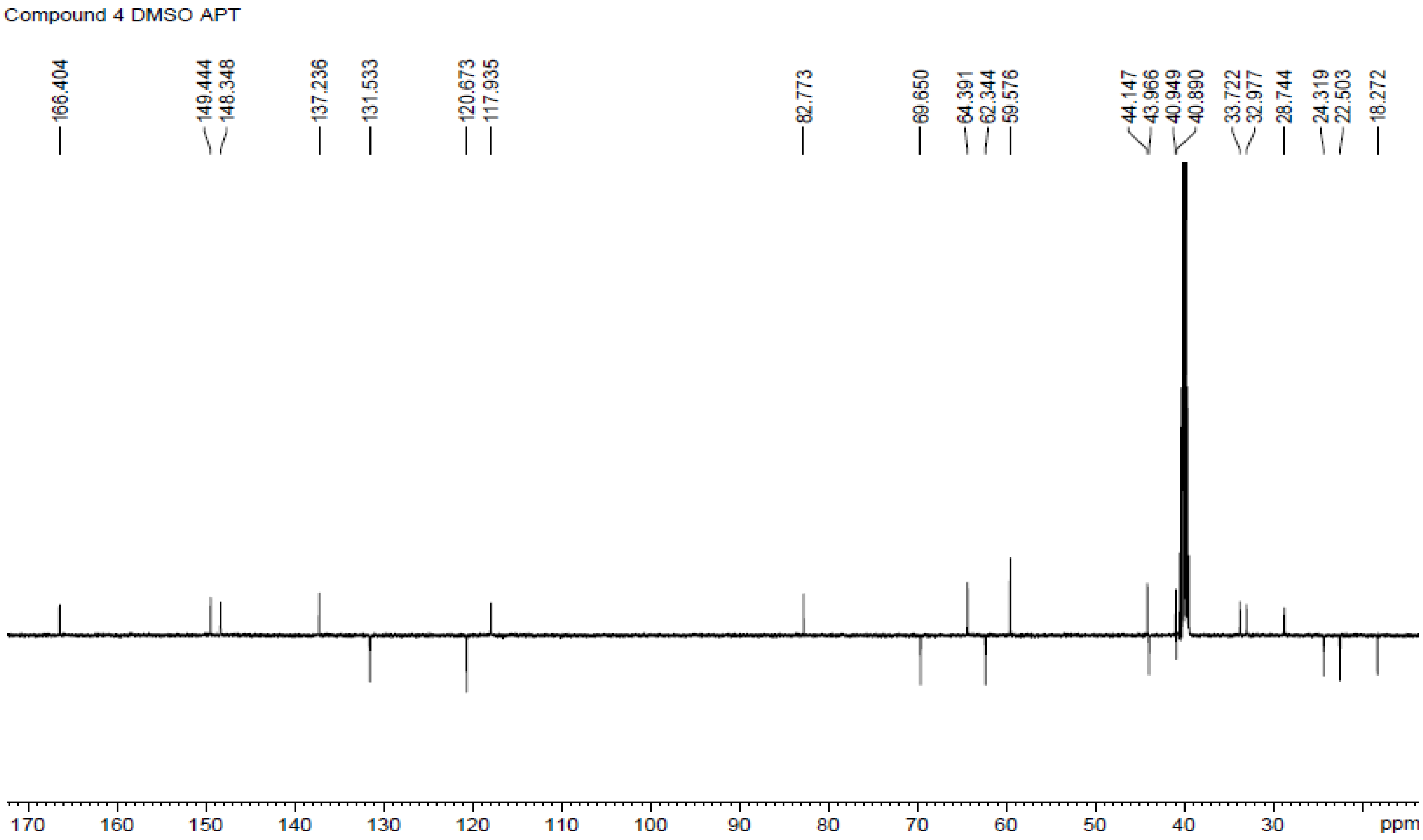

Figure S31. COSY spectrum of punctaporonin K (**4**).

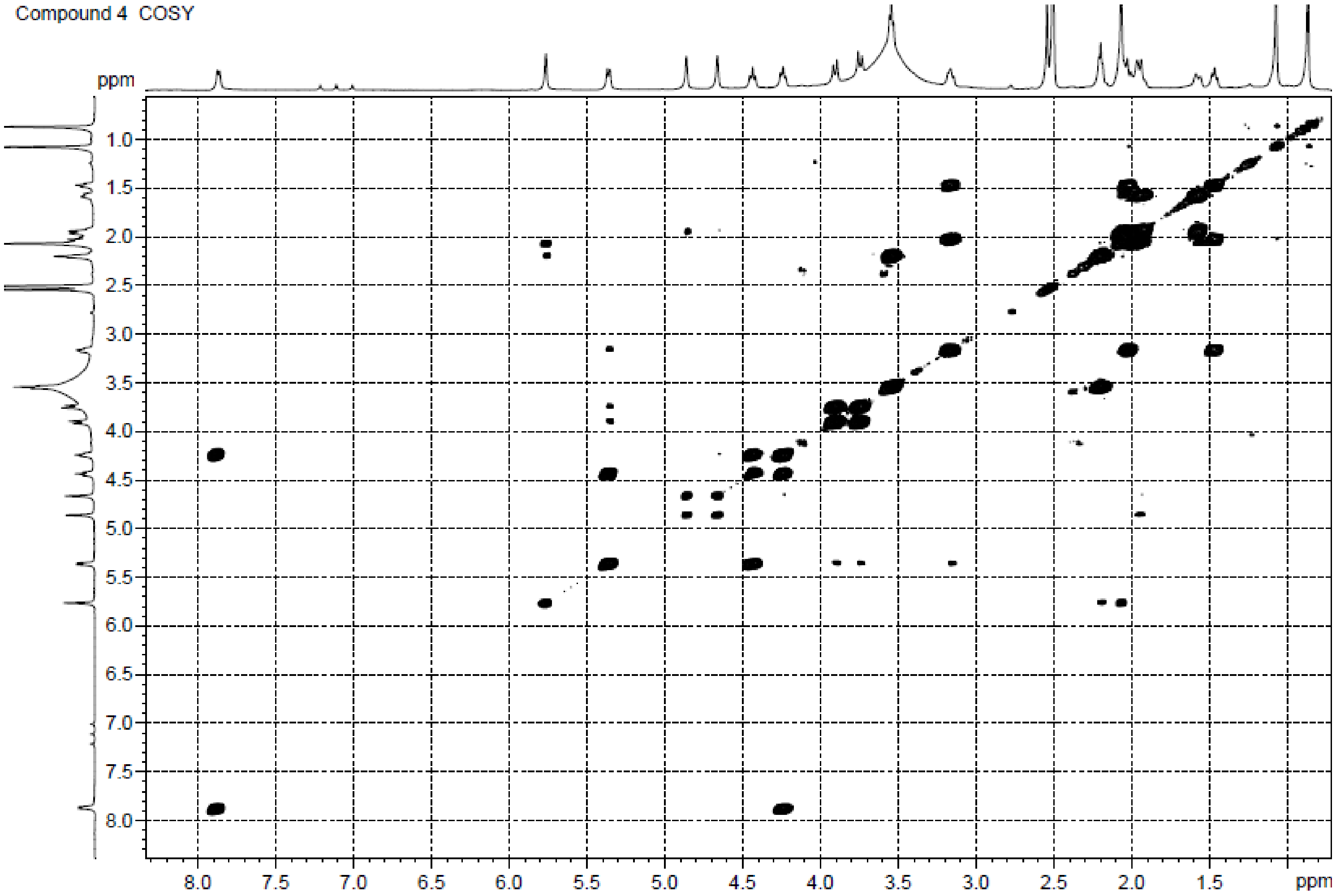

Figure S32. HSQC spectrum of punctaporonin K (**4**).

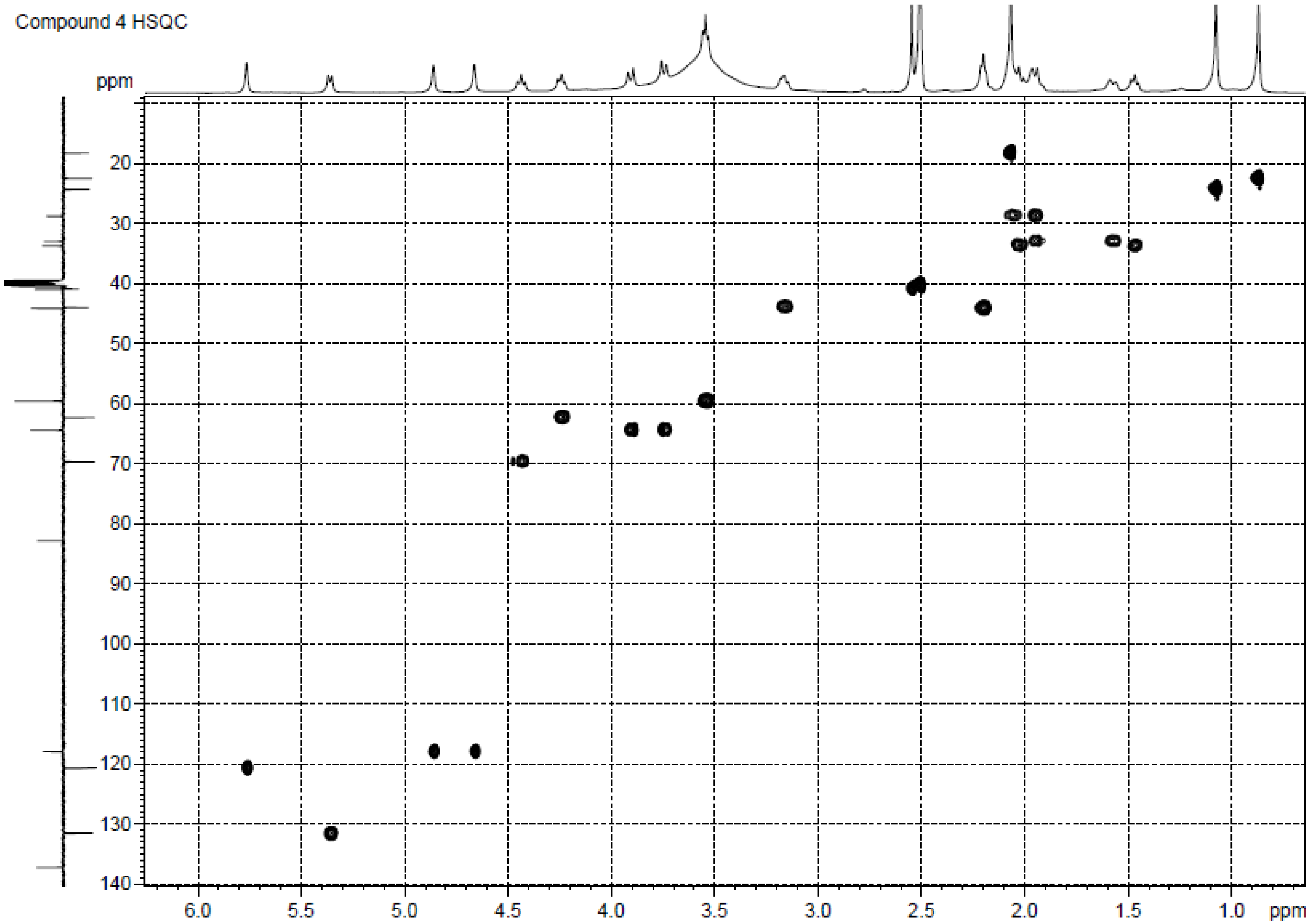

Figure S33. HMBC spectrum of punctaporonin K (**4**).

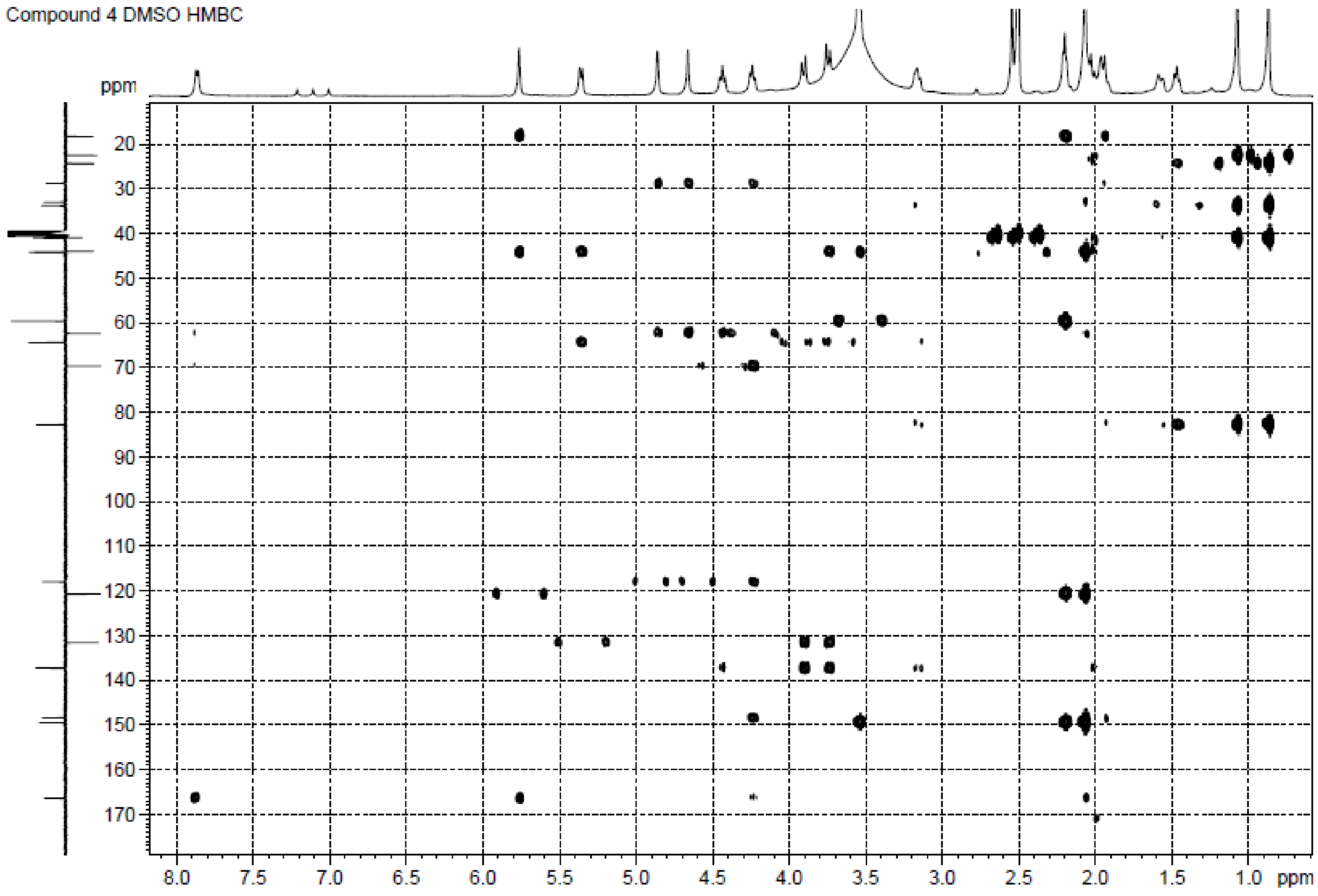

Figure S34. ROESY spectrum of punctaporonin K (**4**).

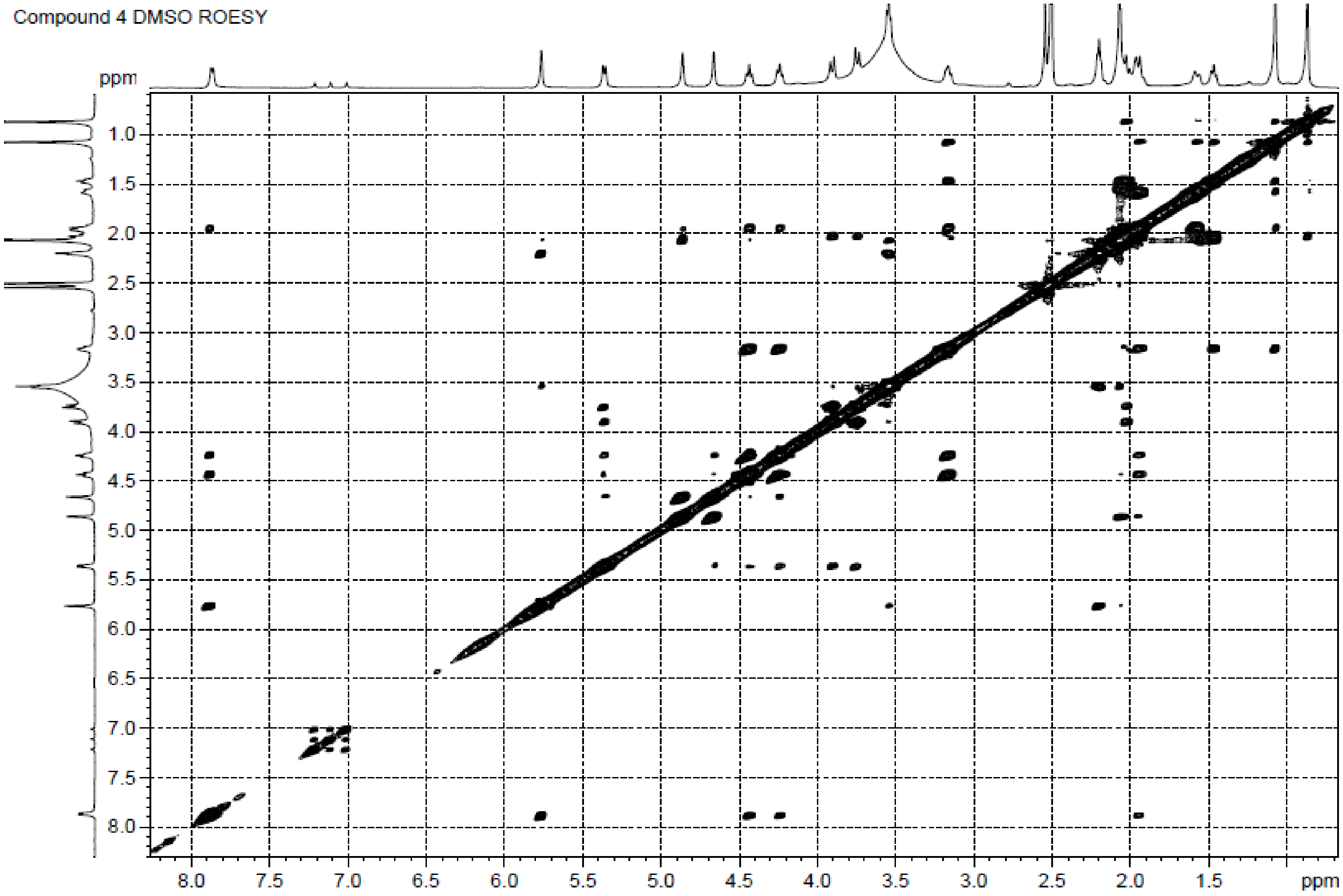

7. The HRESIMS, IR and NMR Data of Punctaporonin L (5)

Figure S35. HRESIMS spectrum of punctaporonin L (**5**).

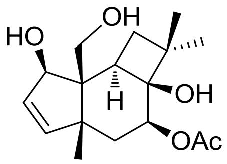

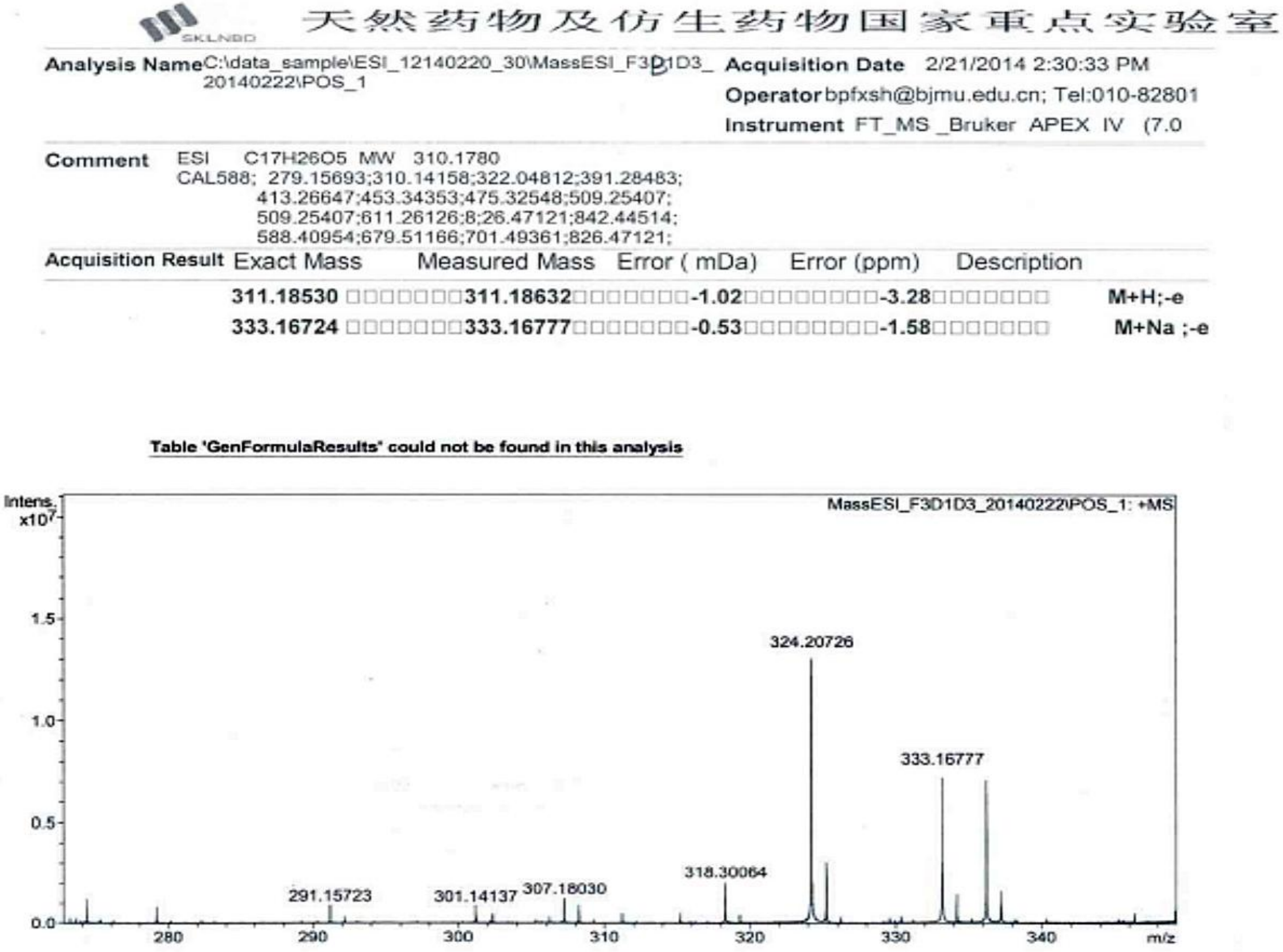

Figure S36. IR spectrum of punctaporonin L (**5**).

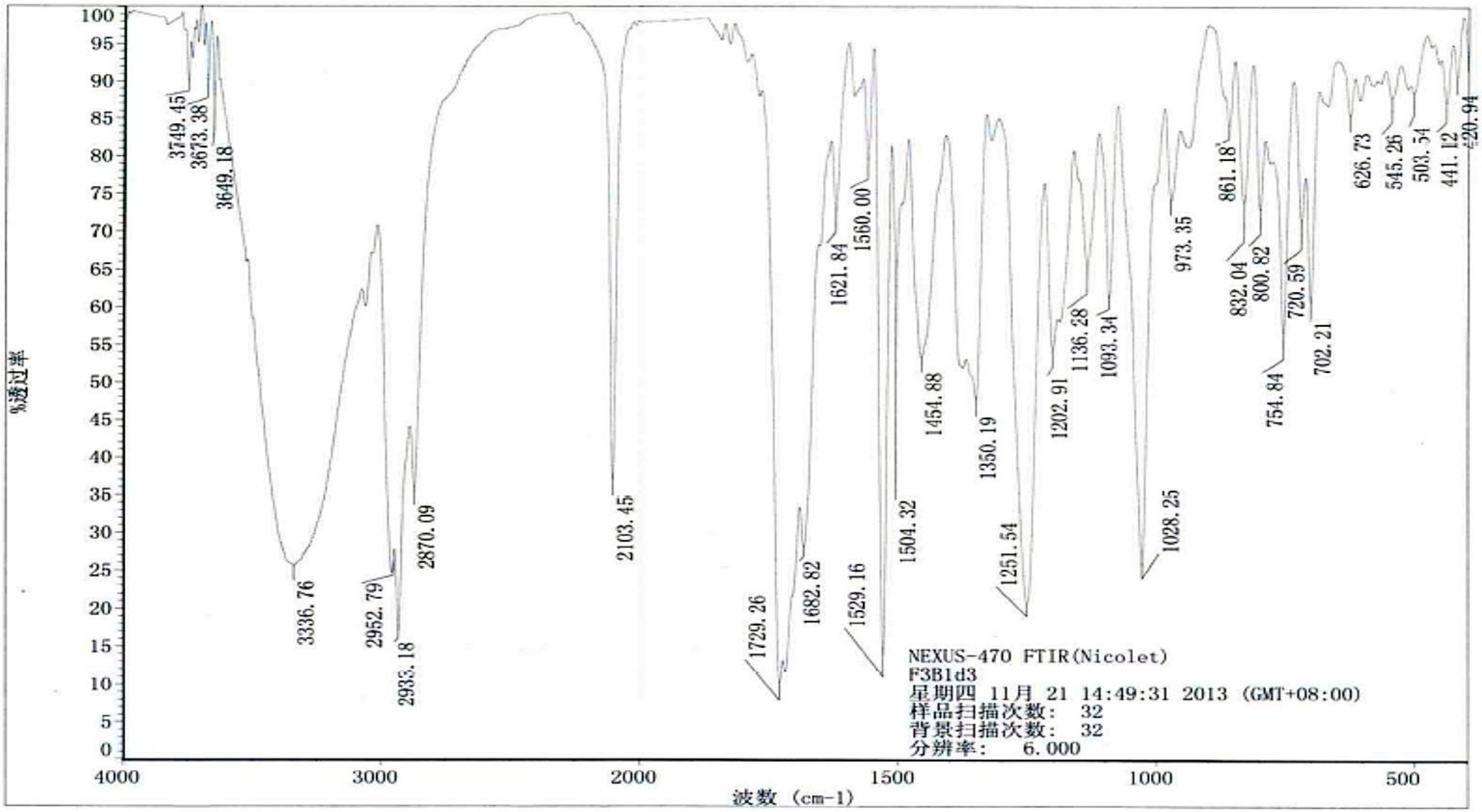

Figure S37. ^{1}H-NMR spectrum of punctaporonin L (**5**).

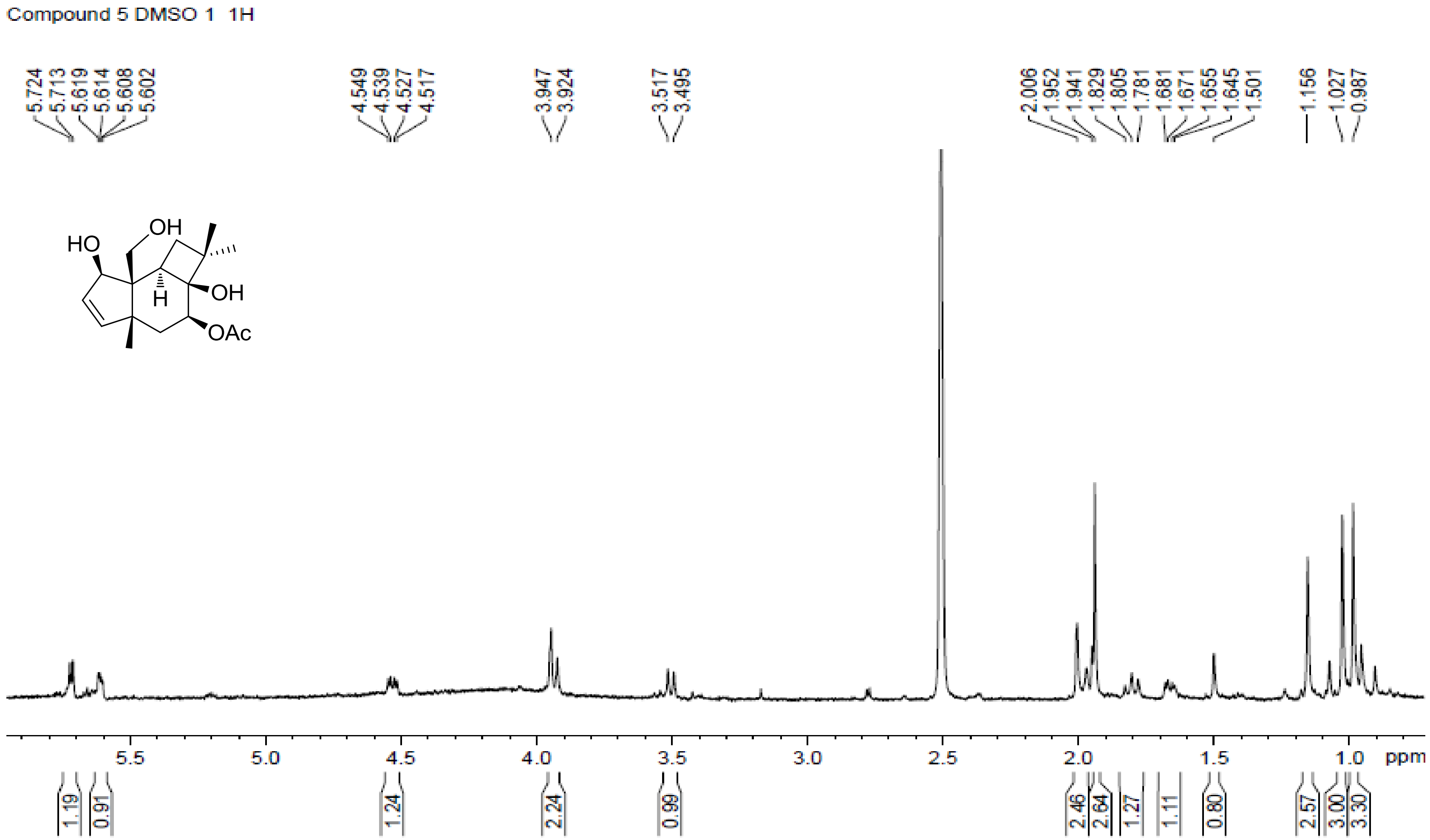

Figure S38. APT spectrum of punctaporonin L (**5**).

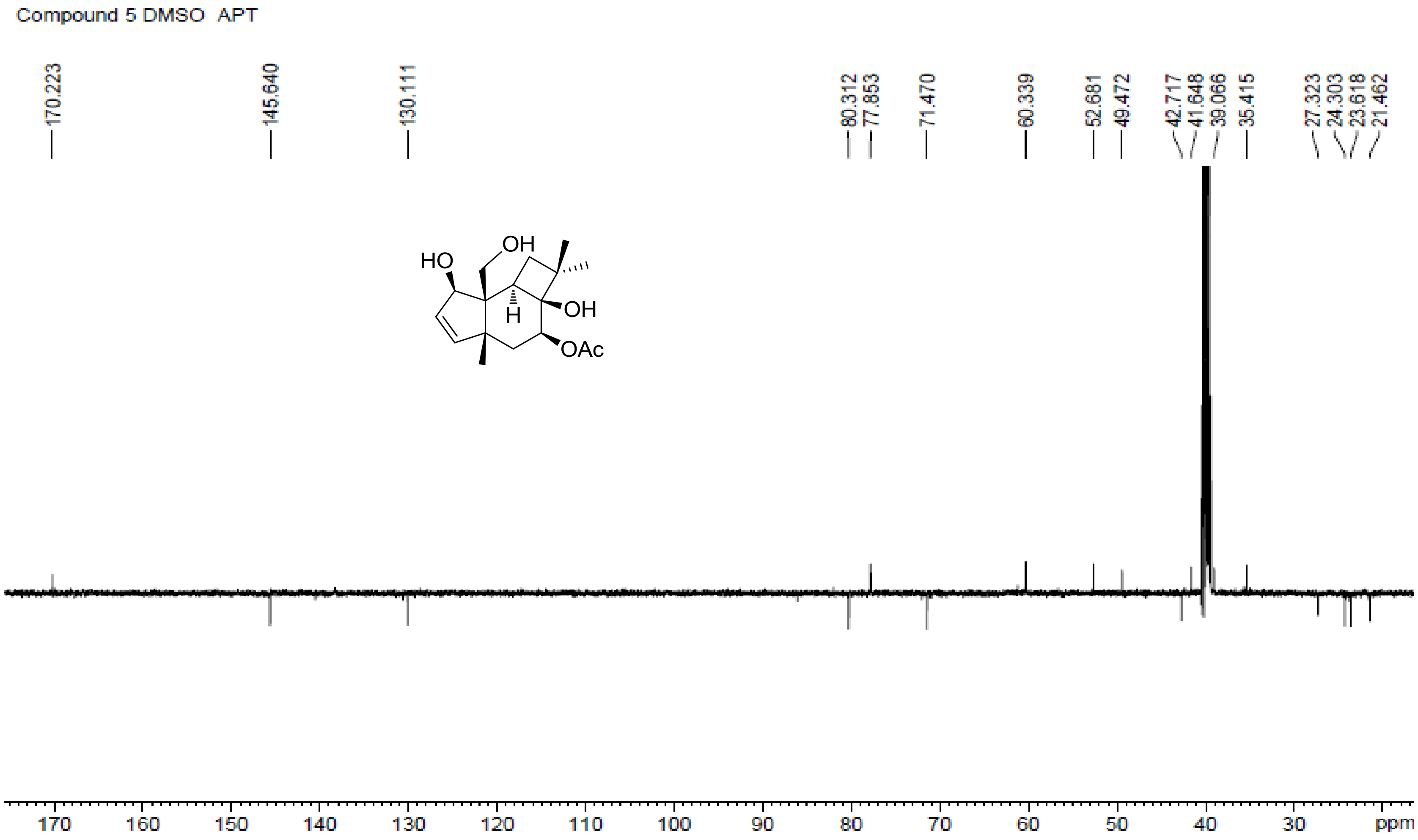

Figure S39. COSY spectrum of punctaporonin L (**5**).

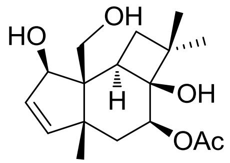

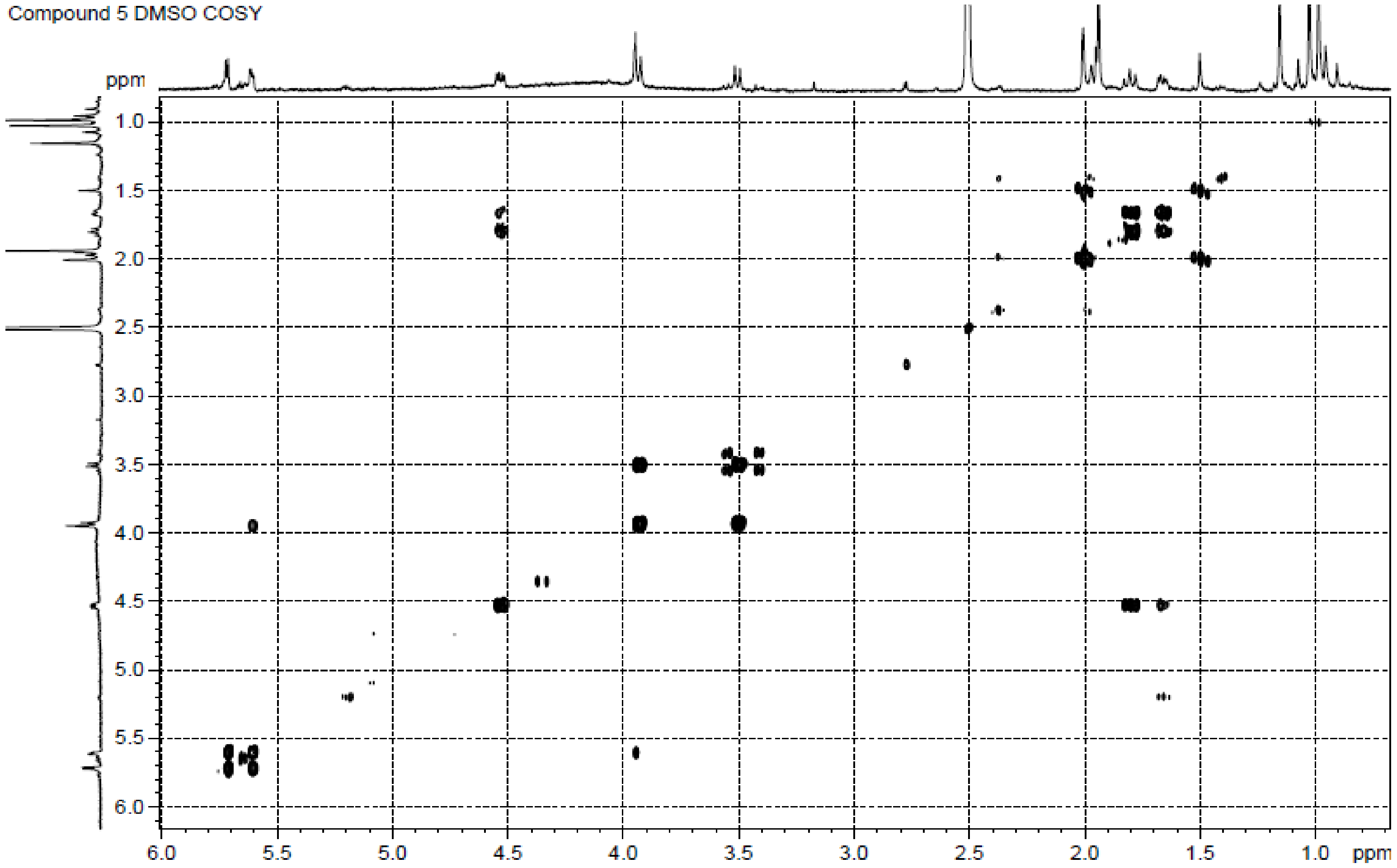

Figure S40. HSQC spectrum of punctaporonin L (**5**).

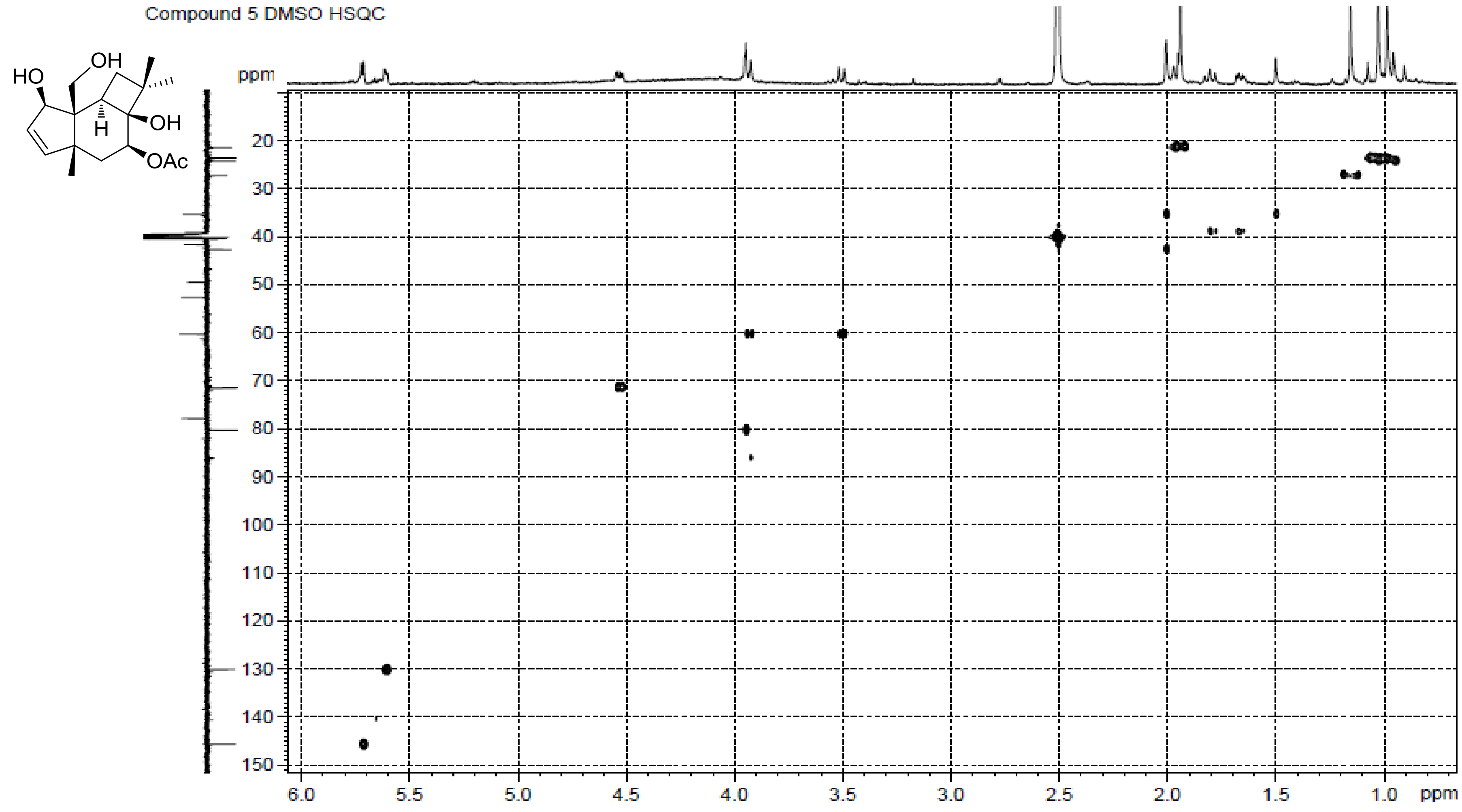

Figure S41. HMBC spectrum of punctaporonin L (**5**).

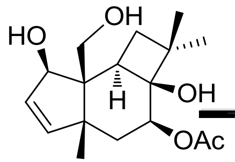

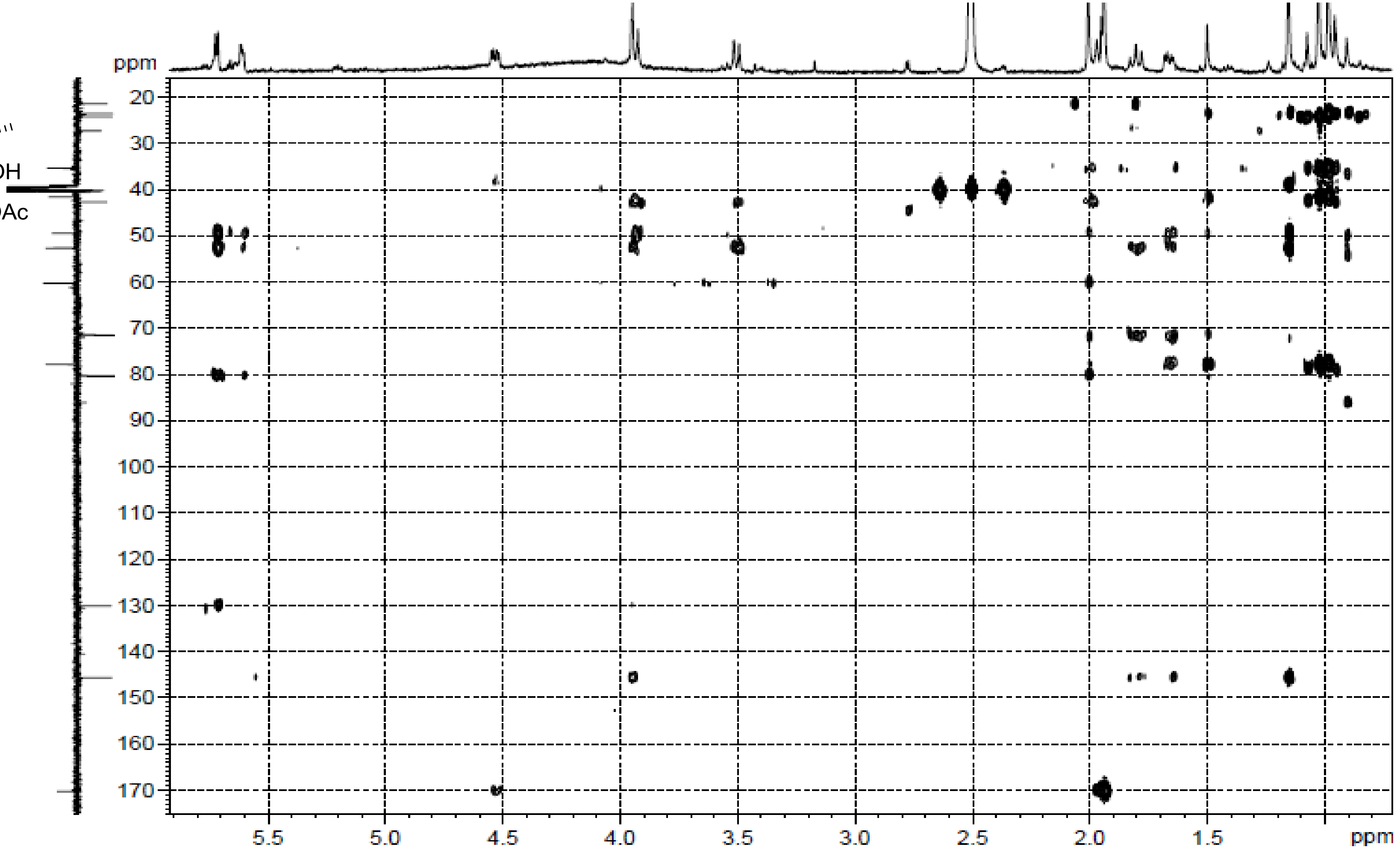

Figure S42. ROESY spectrum of punctaporonin L (**5**).

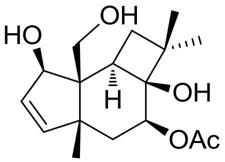

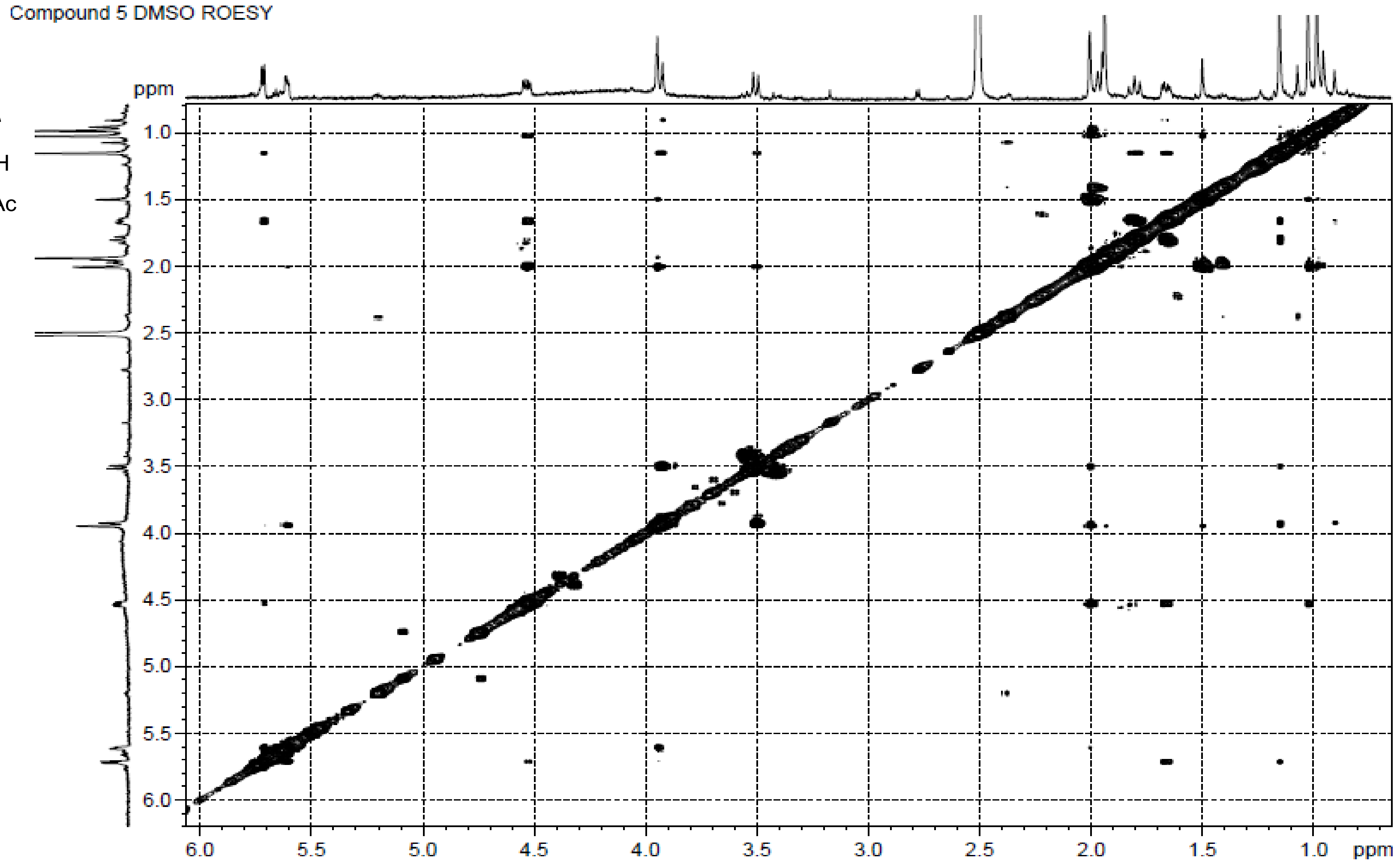

8. The HRESIMS, IR and NMR Data of Punctaporonin M (6)

Figure S43. HRESIMS spectrum of punctaporonin M (**6**).

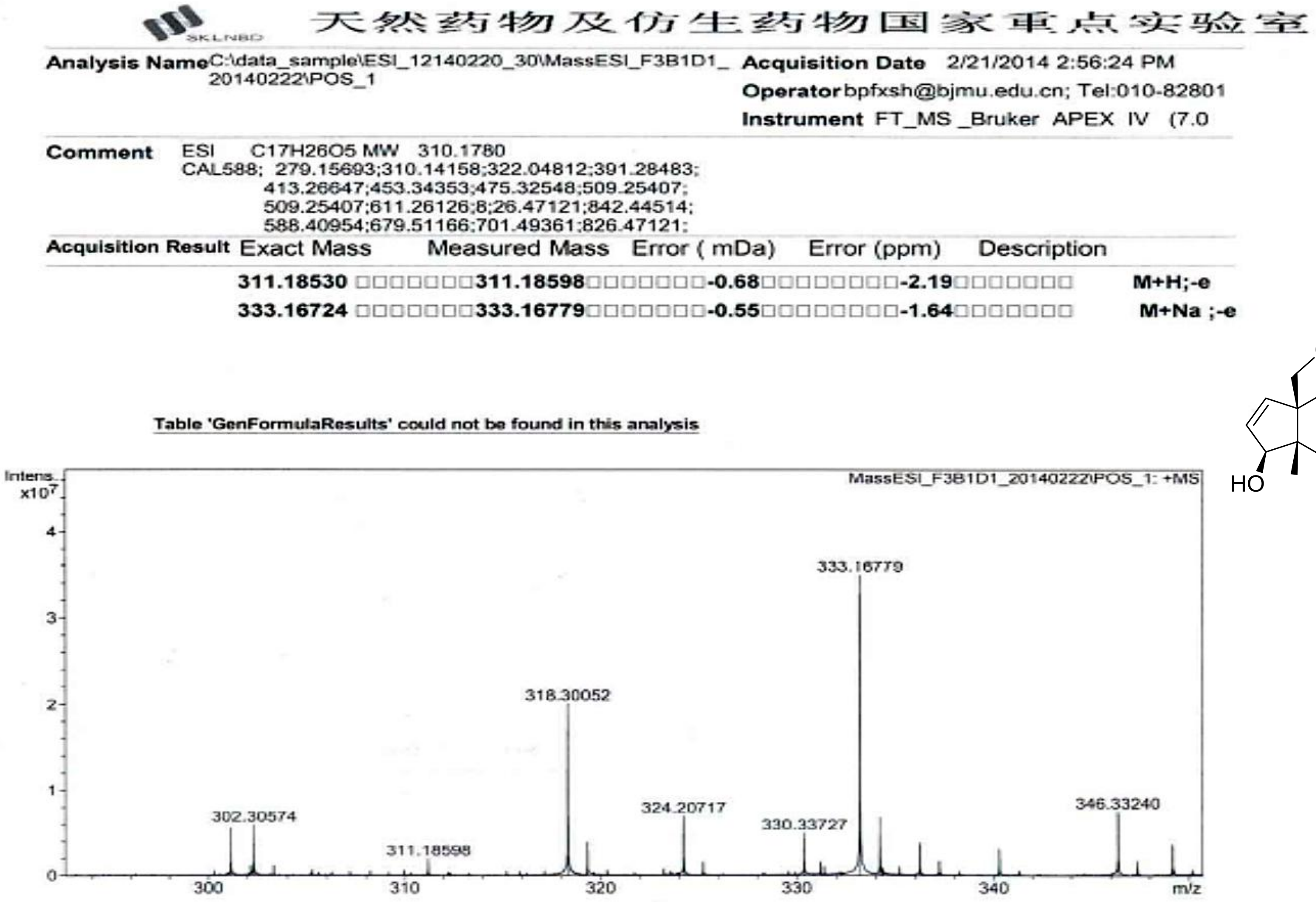

SKLNBD 天然药物及仿生药物国家重点实验室

Analysis Name C:\data_sample\ESI_12140220_30\MassESI_F3B1D1_20140222\POS_1
Acquisition Date 2/21/2014 2:56:24 PM
Operator bpfxsh@bjmu.edu.cn; Tel:010-82801
Instrument FT_MS _Bruker APEX IV (7.0

Comment ESI C17H26O5 MW 310.1780
CAL588; 279.15693;310.14158;322.04812;391.28483;
413.26647;453.34353;475.32548;509.25407;
509.25407;611.26126;8;26.47121;842.44514;
588.40954;679.51166;701.49361;826.47121;

Acquisition Result	Exact Mass	Measured Mass	Error (mDa)	Error (ppm)	Description
	**311.18530**	**311.18598**	**-0.68**	**-2.19**	**M+H;-e**
	**333.16724**	**333.16779**	**-0.55**	**-1.64**	**M+Na ;-e**

Table 'GenFormulaResults' could not be found in this analysis

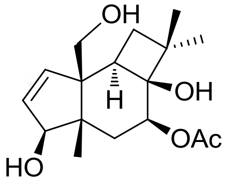

Figure S44. IR spectrum of punctaporonin M (**6**).

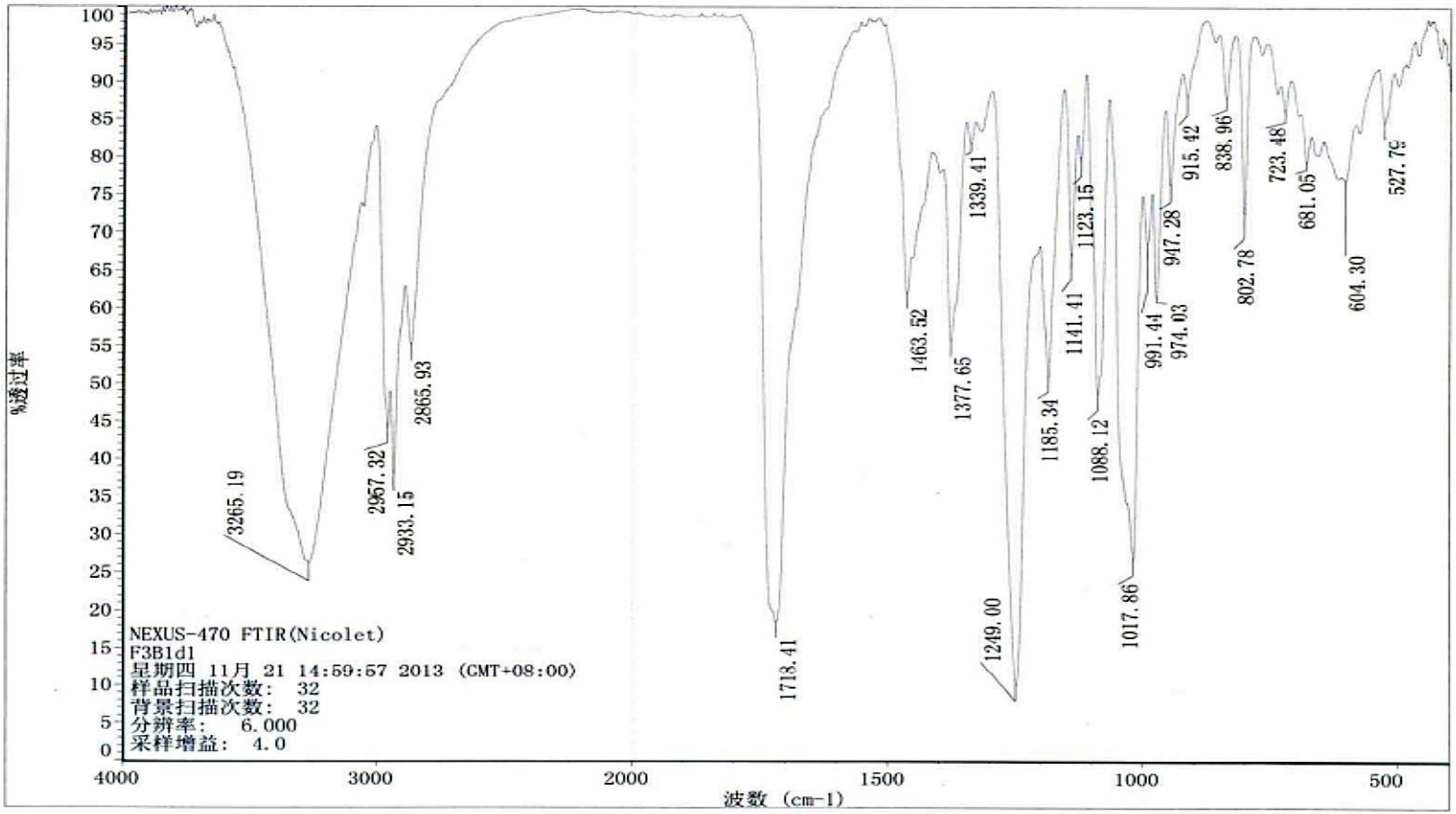

Figure S45. ^{1}H-NMR spectrum of punctaporonin M (**6**).

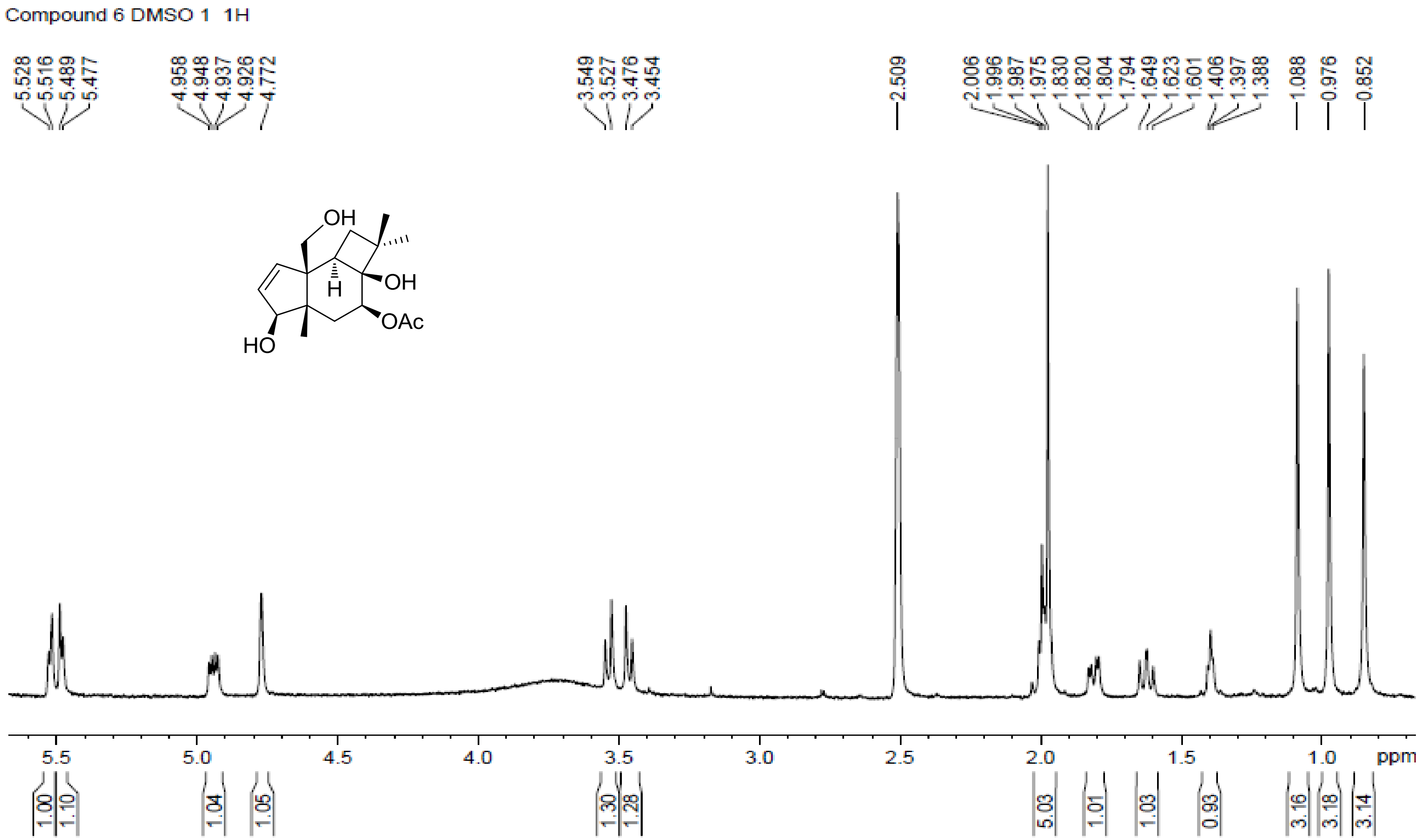

Figure S46. APT spectrum of punctaporonin M (**6**).

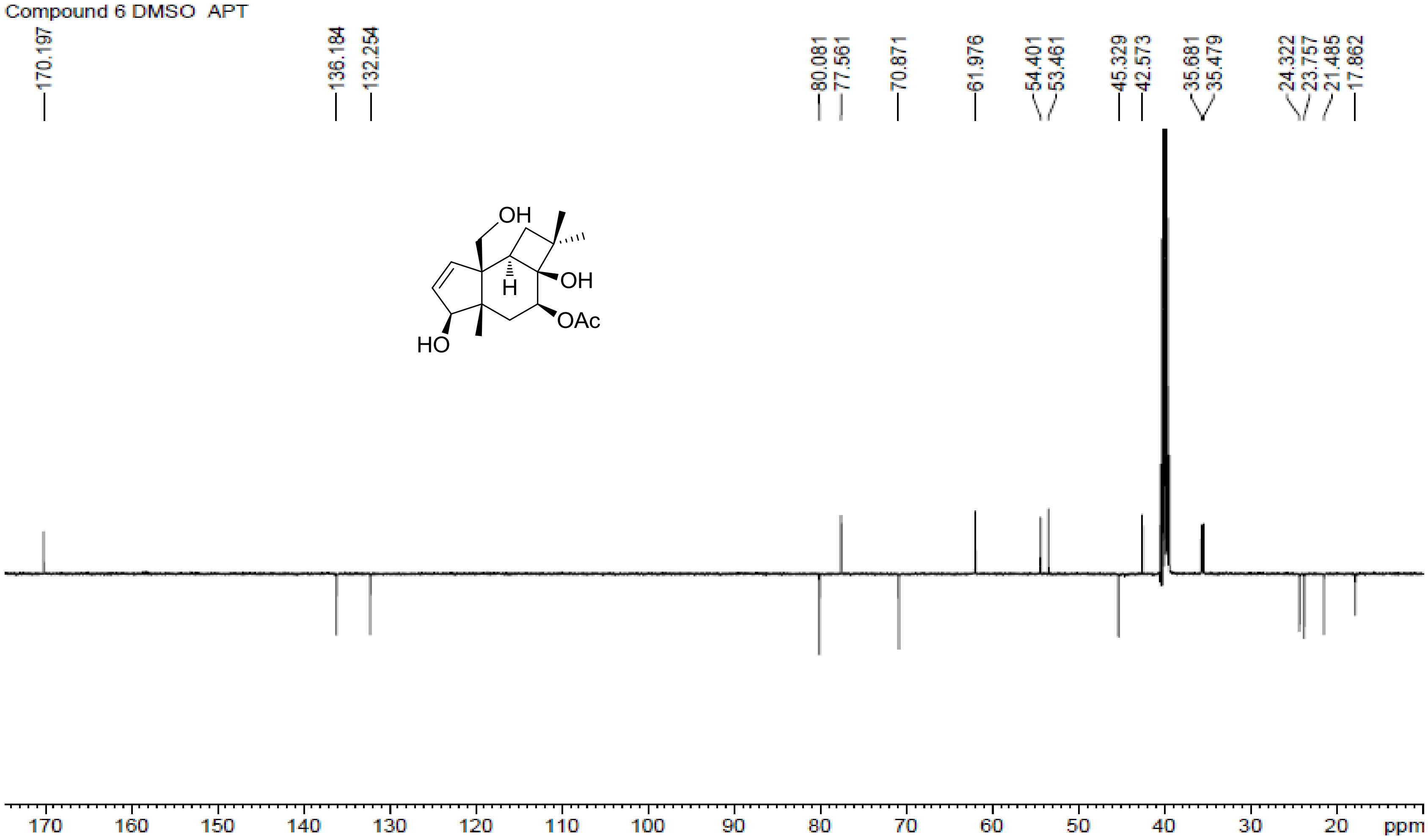

Figure S47. COSY spectrum of punctaporonin M (**6**).

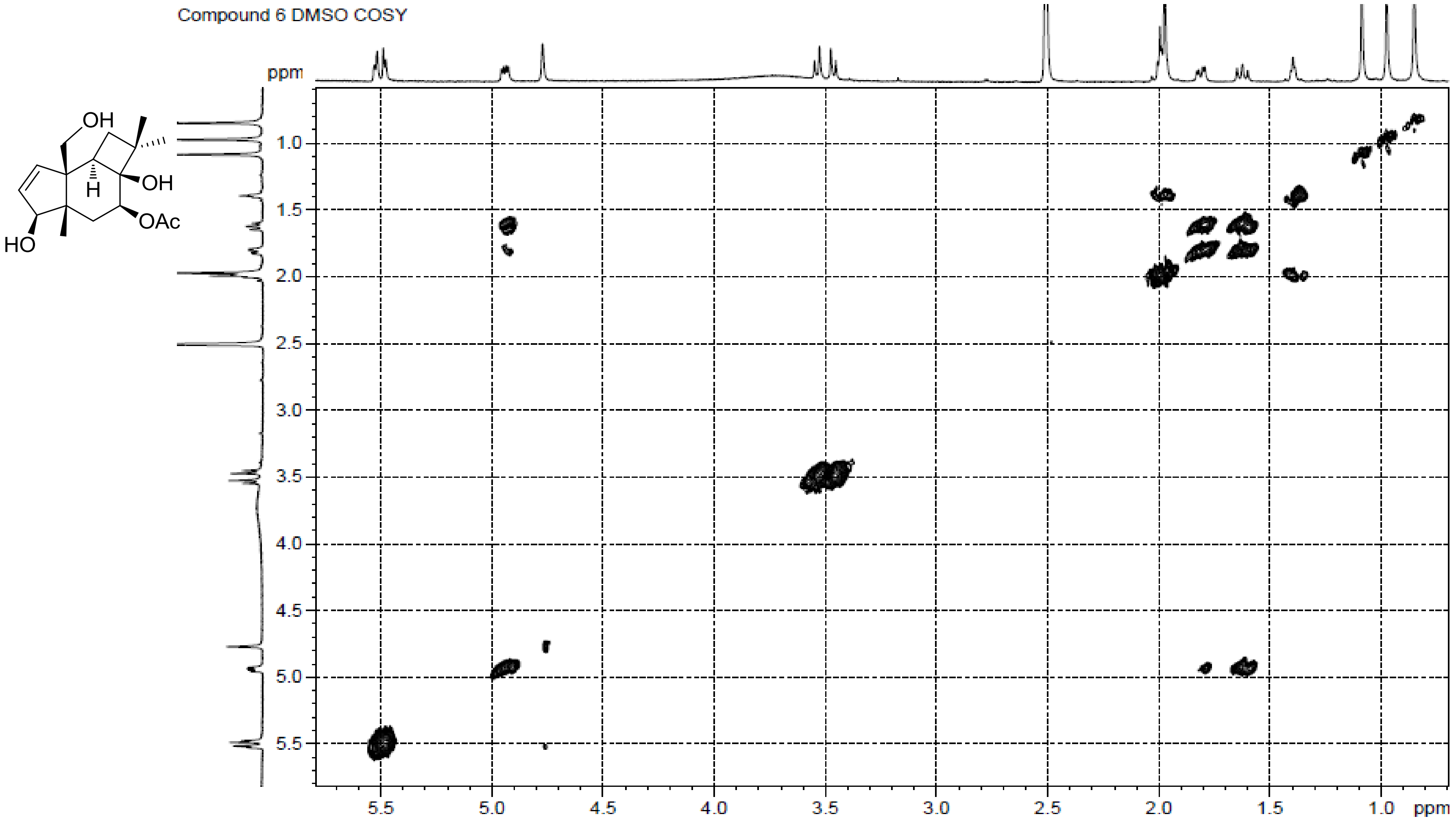

Figure S48. HSQC spectrum of punctaporonin M (**6**).

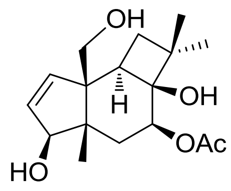

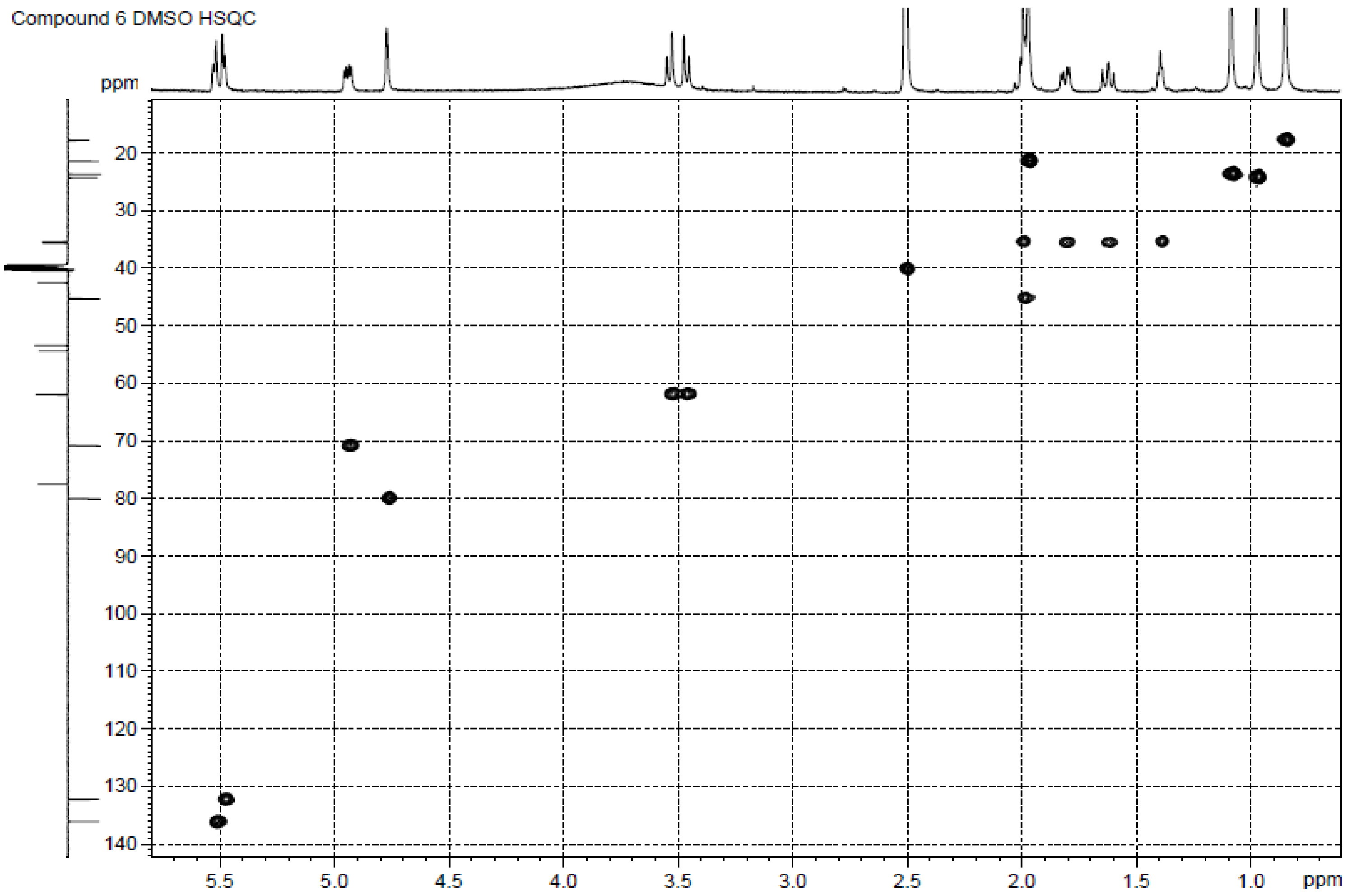

Figure S49. HMBC spectrum of punctaporonin M (**6**).

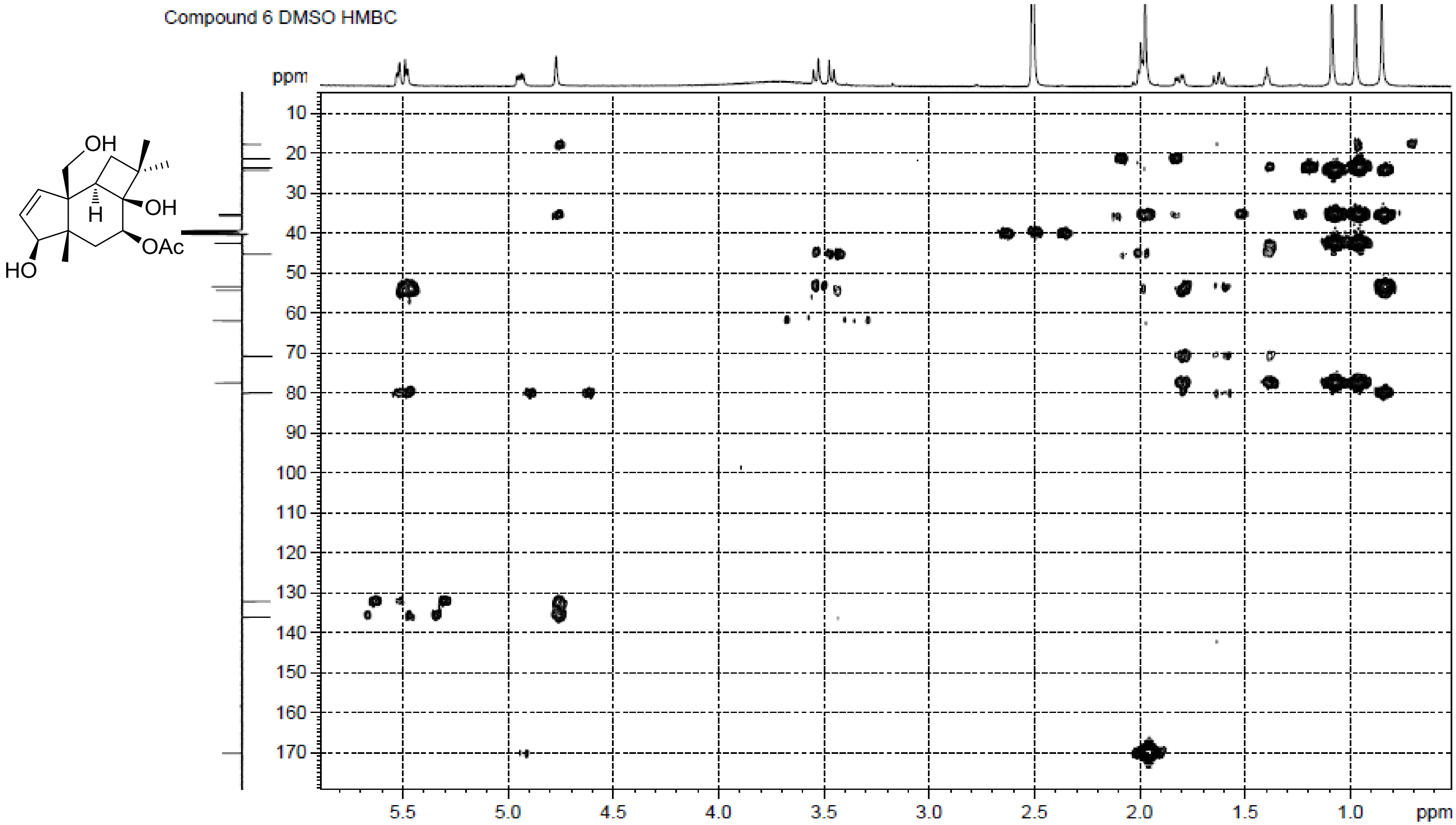

Figure S50. ROESY spectrum of punctaporonin M (**6**).

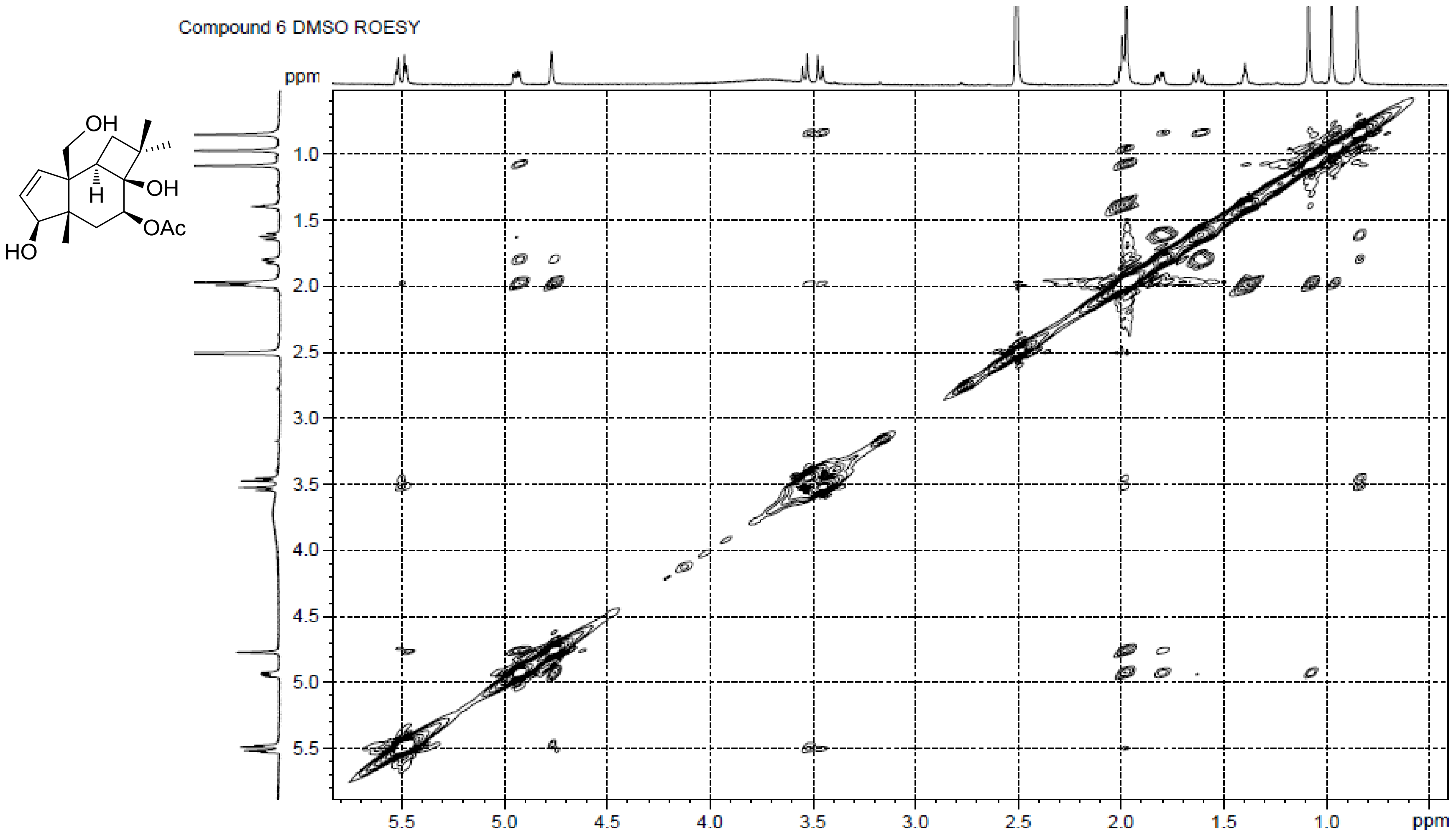

© 2014 by the authors; licensee MDPI, Basel, Switzerland. This article is an open access article distributed under the terms and conditions of the Creative Commons Attribution license (http://creativecommons.org/licenses/by/3.0/).
